# Synthesis and Thermal Studies of Two Phosphonium Tetrahydroxidohexaoxidopentaborate(1-) Salts: Single-Crystal XRD Characterization of [^i^PrPPh_3_][B_5_O_6_(OH)_4_]·3.5H_2_O and [MePPh_3_][B_5_O_6_(OH)_4_]·B(OH)_3_·0.5H_2_O †

**DOI:** 10.3390/molecules28196867

**Published:** 2023-09-29

**Authors:** Michael A. Beckett, Peter N. Horton, Michael B. Hursthouse, James L. Timmis

**Affiliations:** 1 School of Natural Sciences, Bangor University, Bangor LL57 2UW, UK; 2 Chemistry Department, University of Southampton, Southampton SO17 1BJ, UK

**Keywords:** organotriphenylphosphonium salts, π-interactions, pentaborate(1-), phenyl embraces, phosphonium salts, tetrahydroxidohexaoxidopentaborate(1-), X-ray structures

## Abstract

Two substituted phosphonium tetrahydoxidohexaoxidopentaborate(1-) salts, [^i^PrPPh_3_][B_5_O_6_(OH)_4_]·3.5H_2_O (**1**) and [MePPh_3_][B_5_O_6_(OH)_4_]·B(OH)_3_·0.5H_2_O (**2**), were prepared by templated self-assembly processes with good yields by crystallization from basic methanolic aqueous solutions primed with B(OH)_3_ and the appropriate phosphonium cation. Salts **1** and **2** were characterized by spectroscopic (NMR and IR) and thermal (TGA/DSC) analysis. Salts **1** and **2** were thermally decomposed in air at 800 °C to glassy solids via the anhydrous phosphonium polyborates that are formed at lower temperatures (<300 °C). BET analysis of the anhydrous and pyrolysed materials indicated they were non-porous with surface areas of 0.2–2.75 m^2^/g. Rhe recrystallization of **1** and **2** from aqueous solution afforded crystals suitable for single-crystal XRD analyses. The structure of **1** comprises alternating cationic/anionic layers with the H_2_O/pentaborate(1-) planes held together by H-bonds. The cationic planes have offset face-to-face (*off*) and vertex-to-face (*vf*) aromatic ring interactions with the ^i^Pr groups oriented towards the pentaborate(1-)/H_2_O layers. The anionic lattice in **2** is expanded by the inclusion of B(OH)_3_ molecules to accommodate the large cations; this results in the formation of a stacked pentaborate(1-)/B(OH)_3_ structure with channels occupied by the cations. The cations within the channels have *vf*, *ef* (edge-to-face), and *off* phenyl embraces. Both H-bonding and phenyl embrace interactions are important in stabilizing these two solid-state structures.

## 1. Introduction

**Figure 1:**
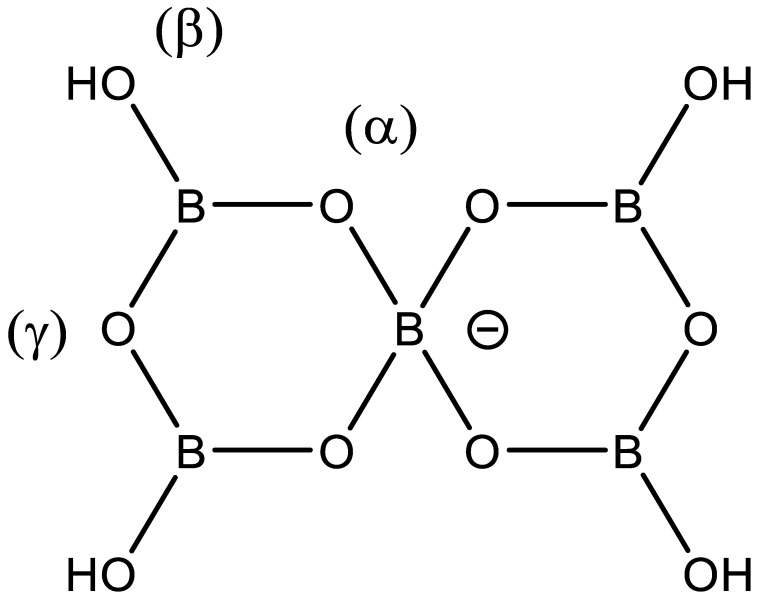
Schematic drawing of tetrahydroxidohexaoxidopentaborate(1-) as found in [^i^PrPPh_3_][B_5_O_6_(OH)_4_]·3.5H_2_O (**1**) and [MePPh_3_][B_5_O_6_(OH)_4_]·B(OH)_3_·0.5H_2_O (**2**). The oxygen H-bond acceptor sites are labelled as in Ref. [24].

Hydrated polyhydroxidooxidoborates and anhydrous polyoxidoborates are a well-known, naturally occurring classes of compounds [1,2,3,4,5,6,7,8,9] with many synthetic analogues [3,9,10,11,12]. Some of these compounds are industrially important bulk chemicals (e.g., Na_2_B_4_O_5_(OH)_4_·3H_2_O, tincalconite and the synthetic borax pentahydrate largely produced from Na_2_B_4_O_6_(OH)_2_·3H_2_O (kernite) and Na_2_B_4_O_5_(OH)_4_·8H_2_O, borax (tincal)) with many applications [13,14,15], whilst others, such as β-BaB_2_O_4_ (BBO), have found more specialist niche applications in NLO materials [9,16]. Structurally, these polyoxidoborates are a diverse class of compounds with the polyoxidoborate moieties as discrete insular anions or as more highly condensed polymeric 1-D, 2-D or 3-D anionic networks, and the associated cations as simple *s*-, *p*-, *d*- or *f*-block element cations, cationic *p*- or *d*-block complexes or non-metal/organic based [1,2,3,4,5,6,7,8,9,10,11,12,13,14,15,16]. With some late transition metals (e.g., Cu^II^, Zn^II^, Cd^II^ and Ni^II^), oxidoborates can also function as *O*-donor ligands [17]. Examples of hydroxidooxidopolyborate salts with phosphorus-containing cations are rare and are currently limited to [Ph_3_PNPPh_3_][B_3_O_3_(OH)_4_]·2.5H_2_O [18], [Ph_3_PNPPh_3_][B_5_O_6_(OH)_4_]·1.5 H_2_O [18] and [PPh_4_][B_5_O_6_(OH)_4_]·1.5H_2_O [19]; the latter compound has been structurally characterized by a single-crystal X-ray diffraction (sc-XRD). We have previously published thermal studies and BET analysis on materials that were thermally obtained from pentaborate(1-) salts containing organic cations [20,21,22]. This manuscript extends our structural studies on phosphonium salts of pentaborate(1-) anions and examines them using BET analysis on materials derived thermally from these salts. A schematic drawing of the tetrahydroxidohexaoxidopentaborate(1-) anion, hereafter generally abbreviated to pentaborate(1-) [23], is shown in Figure 1.

## 2. Results and Discussion

### 2.1. Synthesis

**Scheme 1:**
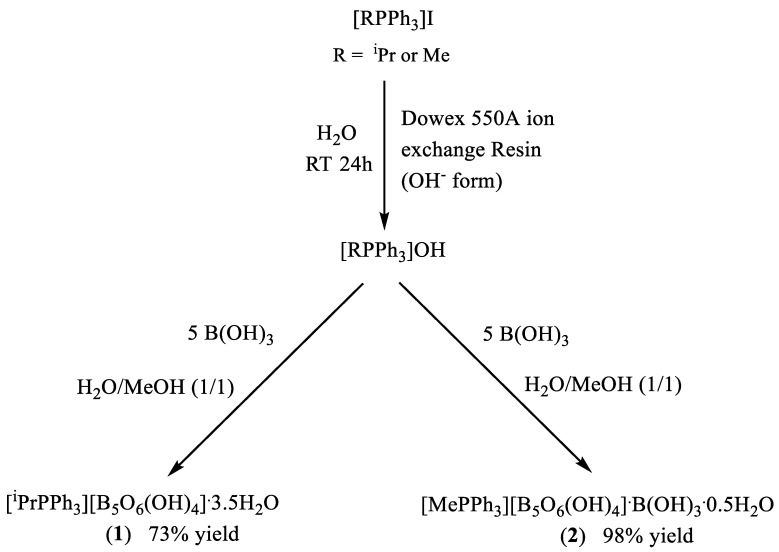
Synthesis of compounds **1** and **2**.

The two new tetraorganophosphonium pentaborate(1-) salts [^i^PrPPh_3_][B_5_O_6_(OH)_4_]·3.5H_2_O (**1**) and [MePPh_3_][B_5_O_6_(OH)_4_]·B(OH)_3_·0.5H_2_O (**2**) were obtained by crystallization from basic aqueous solutions primed with B(OH)_3_ and the appropriate substituted phosphonium cation, as shown in Scheme 1. The phosphonium iodide salts were converted to their hydroxide salts by use of an ion-exchange resin and B(OH)_3_ was used as the boron source for the pentaborate(1-) salts. The boron-containing substrate B(OH)_3_, is only present in aqueous solution as B(OH)_3_ under acidic conditions [25,26,27]. At a higher pH, it is present as rapidly attained equilibrium concentrations of various hydroxidooxidopolyborate anions and [B(OH)_4_]^−^ [25,26,27]. The observed crystalline products arise through the cation templated self-assembly/crystallization processes [28,29,30,31], as are often observed in related systems involving non-metal cations derived from organic amines and B(OH)_3_ [10,32].

Compounds **1** and **2** were prepared in excellent yields and were characterized spectroscopically (multinuclear NMR, IR) and thermally (TGA/DSC). Porosity data (BET) were also obtained on materials derived thermally in air from **1** and **2**. Crystallization of the crude products **1** and **2** from H_2_O afforded crystals suitable for sc-XRD studies (Section 2.4). The co-crystallization of B(OH)_3_ with pentaborate(1-) anions, as in **2**, has occasionally been observed in structures with large organic cations [18,33,34,35,36].

### 2.2. Thermal Studies

Organic cation polyborates are known to thermally decompose in air with the formation of glassy B_2_O_3_ at 800 °C [10,34]. The closely related tetraphenylphosphonium pentaborate salt, [PPh_4_][B_5_O_6_(OH)_4_]·1.5H_2_O, is reported to be thermally decomposed in a similarly manner [19]. In previous studies, water is lost at lower temperatures with the formation of ‘anhydrous’ pentaborates and this is followed at higher temperatures by oxidation of the cation, gaseous evolution, and the formation of darkened intumesced solids. At higher temperatures again, these solids shrink down to form glassy residual materials of B_2_O_3_ [10,24]. The thermal decomposition of **1** and **2** was studied by TGA/DSC analysis in air over the temperature range 20–800 °C.

The data for **1** were consistent with the initial loss of 5.5 × H_2_O in the first stage (<275 °C) in an endothermic process to form anhydrous [^i^PrPPh_3_][B_5_O_8_]. This dehydration stage involved the loss of interstitial H_2_O (3.5 × H_2_O) and condensation/cross-linking of the B-OH groups (2.0 × H_2_O) as one large continuous step (see Supplementary Information, Figure S10 for TGA plots). The TGA plot of **2** had a similar profile, with loss of 4.0 × H_2_O in the first stage (100–275 °C) and the formation of anhydrous [MePPh_3_][B_6_O_9.5_]. Since **2** is a 1:1 B(OH)_3_/pentaborate(1-) co-crystal, this material is formulated as an anhydrous hexaborate [18,33,34]. This endothermic dehydration step for **2** is a two-stage process involving the loss of interstitial H_2_O and partial condensation/cross-linking of the pentaborate B-OH groups (2.0 × H_2_O, 100–150 °C), with further condensation/cross-linking of the pentaborate B-OH groups (2.0 × H_2_O, 150–275 °C). This two-stage water loss is qualitatively very similar to that observed for [PPh_4_][B_5_O_6_(OH)_4_]^.^1.5H_2_O [19]. 

It was anticipated that, upon further heating (275–800 °C), **1** and **2** would leave, after oxidation of the cations during the exothermic second stages, with glassy residues comprised of 2.5 or 3.0 equivalents of B_2_O_3_, respectively. However, the residual masses from **1** and **2** were higher than calculated, indicating that they both contained additional, non-B_2_O_3_ material. It has been noted that phosphonium salts, with simple non-polar substituents, generally decompose cleanly with little residue [37], and our previous studies on the thermal decomposition of phosphonium polyborates are consistent with this [18,19]. However, some phosphonium salts are also known to decompose with residual material [37]. The additional residual material from **1** and **2** possibly arises through the incorporation of phosphorus and/or an organic char slowing down the oxidation process. 

Porosity data (BET analysis [38]) of organic pentaborates salts and their thermally derived anhydrous, pyrolysed and residual glasses have been reported and the results indicated that they were non-porous [20,21,22]. Compounds **1** and **2** both possess unusual solid-state pentaborate structures (see sc-XRD studies, Section 2.4), with **1** layered and **2** having its cations stacked in channels. We were, therefore, interested in obtaining porosity data on the thermally derived intermediate materials from these phosphonium pentaborates to see if these structural modifications are influential. Thus, samples of ‘anhydrous’ and ‘pyrolysed’ materials were obtained from **1** and **2** by heating ca. 0.5 g samples in a furnace in air for 24 h at 300 °C and 625 °C, respectively. These materials had surface areas of 0.2–2.75 m^2^/g and were essentially non-porous, with similar values to those obtained for materials derived thermally from [PPh_4_][B_5_O_6_(OH)_4_]·1.5H_2_O [39] and organic pentaborates [20,21,22], again suggesting that the intumesced solids have ‘foamlike’ gas-encapsulated macroporous structures [40].

### 2.3. Spectroscopic Studies

IR and NMR (^1^H, ^13^C, ^11^B and ^31^P) data for **1** and **2** are reported in the experimental section. These spectroscopic data are in agreement with the expected data for the anions and cations found in **1** and **2**.

The IR spectra, obtained as KBr discs, show the expected broad H-bonded O-H stretches (ca. 3300 cm^−1^) and strong B-O stretches/bends (1450–620 cm^−1^) [41] associated with the pentaborate(1-) anions. Specifically, a diagnostic strong band (B_trig_-O (sym.) at ca. 925 cm^−1^ [34]) for the anion was observed at 918 and 924 cm^−1^ for **1** and **2,** respectively, helping to confirm their identities.

Compounds **1** and **2** are insoluble in organic solvents but ‘dissolve’ in H_2_O with decomposition of the pentaborate(1-) anion by the borate equilbria processess that are also involved in their formation [25,26,27]. The cations in **1** and **2** are not affected by this, and their presence was confirmed as substituted phosphonium cations by ^1^H, ^13^C and ^31^P spectroscopic analysis. Thus, ^31^P spectra of **1** and **2** both show only one signal at the expected chemical shift for their phosphonium cations [42]. The ^11^B spectra of **1** and **2** (in D_2_O) show three signals, corresponding to the tetrahedral boron centre of [B_5_O_6_(OH)_4_]^−^ (*ca*. +1 ppm), [B_3_O_3_(OH)_4_]^−^ (ca. +13 ppm) and (B(OH)_3_/[[B(OH)_4_]^−^ (*ca*. +18 ppm) in the form of ‘signature spectra’, as was previously observed [43]. These signals arise from the equilbrium concentrations of the borate anions present from the ‘disolution’ of the original pentaborate(1-) anion. 

### 2.4. X-ray Crystallography

**Figure 2:**
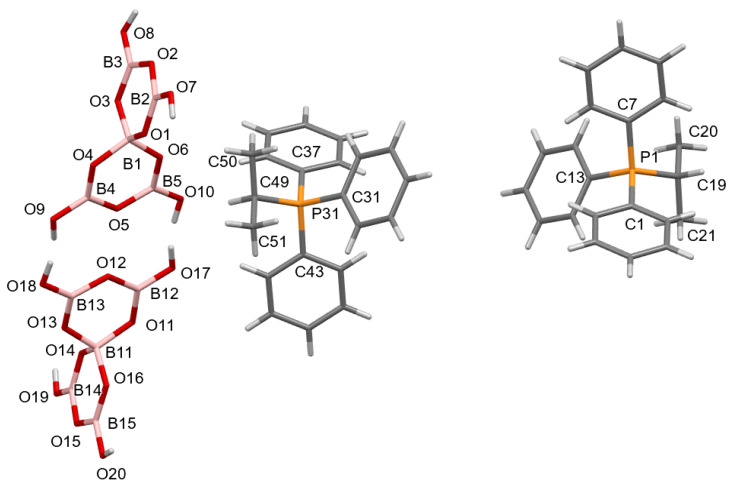
Drawing of the structure of [^i^PrPPh_3_][B_5_O_6_(OH)_4_]·3.5H_2_O (**1**), showing selected crystallographic atomic numbering schemes. The seven waters of crystallization have been omitted for clarity. The O atoms of these H_2_O molecules are numbered O61–O67. Only the lowest numbered carbon in each aryl ring is labelled; the other five carbons are numbered sequentially. H atoms take the same label number as the heavy atoms to which they are attached.

**Figure 3:**
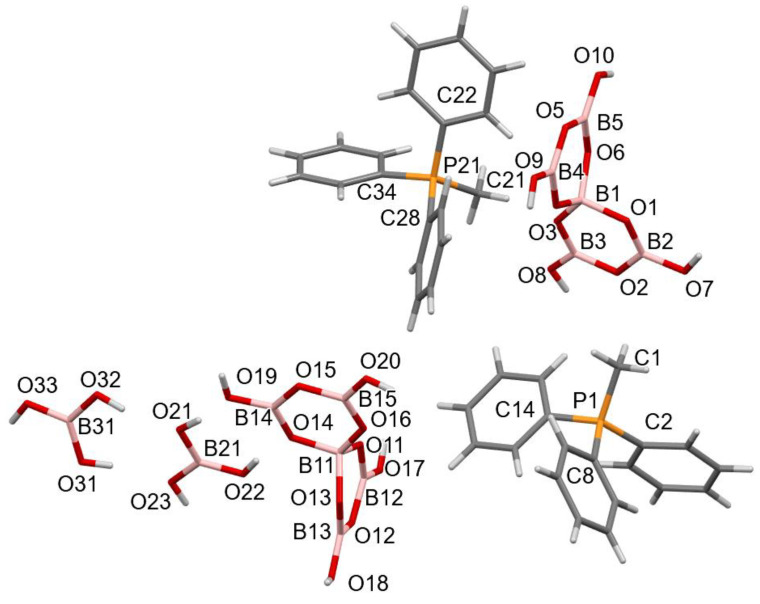
Drawing of the structure of [MePPh_3_][B_5_O_6_(OH)_4_]·B(OH)_3_·0.5H_2_O (**2**) showing the crystallographic atomic numbering scheme. The waters of crystallization have been omitted for clarity and are disordered over four sites. The O atoms of these waters are labelled O41–O43. Only the lowest numbered carbon in each phenyl ring is labelled; the other five carbons are numbered sequentially around the ring. H atoms take the same label number as the heavy atoms to which they are attached.

There are two independent isopropyltriphenylphosphonium(1+) cations, two independent tetrahydroxidohexaoxidopentaborate(1-) anions, and seven waters of crystallization within the unit cell of **1**. The asymmetric unit cell of **2** contains two independent tetrahydroxidohexaoxidopentaborate(1-) anions and two independent methyltriphenylphosphonium(1+) cations. Additionally, **2** also contains two independent B(OH)_3_ molecules and a single disordered H_2_O of crystallization. These crystallographic studies are in agreement with the formulation of **1** and **2** as ionic phosphonium(1+)/pentaborate(1-) salts, as indicated by their spectroscopic and thermal analysis. The co-crystallization of B(OH)_3_ is not uncommon in recrystallized samples of pentaborate(1-) salts containing bulky cations [18,33,34,35,36]. Drawings of the structures of **1** and **2** showing atomic numbering are shown in Figure 2 and Figure 3, respectively. Selected crystallographic information is available in the experimental section and full details can be found in the Supplementary Information.

The tetrahydroxidohexaoxidopentaborate(1-) anion is crystallographically well-known [10] and has the gross structure of two fused, slightly puckered (‘planar’) boroxole (B_3_O_3_) rings sharing a spiro 4-coordinate boron centre; all other boron atoms are 3-coordinate, and are bound solely to oxygen atoms within the rings, or to *exo* hydroxido groups (see Figure 1). B-O bonds lengths and OBO and BOB angles within these compounds are within normal limits [10,20,24]. The B-O distances involving 4-coordinate boron centres range from 1.455(3) to 1.487(3) Å (*av*. 1.473(3) Å) and 1.434(4) to 1.481(4) Å (*av*. 1.462(4) Å), whilst B-O distances involving 3-coordinate boron centres are shorter and range from 1.344(2) to 1.401(3) Å (*av*. 1.369(3) Å) and 1.334(4) to 1.395(4) Å (*av*. 1.359(4) Å) in **1** and **2**, respectively. The OBO angles involving 4-coordinate (*sp^3^* hybridized, tetrahedral) boron centres range from 106.24(17) to 111.19(16)^o^ (*av*. 109.5(2)^o^) and 107.5(2) to 110.9(2)^o^ (*av*. 109.5(2)^o^), whilst OBO angles involving 3-coordinate (*sp^2^* hybridized, trigonal planar) borons are larger and range from 116.21(19) to 122.2(2)^o^ (*av*. 120.0(2)^o^) and 114.3(3) to 123.7(3) (*av.* 120.0(3)^o^) for **1** and **2**, respectively. The BOB angles within the boroxole rings range from 118.40(17) to 123.39(18)^o^ (*av*. 121.46(18)^o^) and 116.7(2) to 124.2(2)^o^ (*av*. 121.4(3)^o^) for **1** and **2** respectively, indicative of these oxygens being *sp^2^* hybridised [44]. The B-O distances and OBO angles within the B(OH)_3_ molecules of **2** are normal for B(OH)_3_ and are also within the ranges found for the trigonal borons of the pentaborate(1-) rings in **1** and **2**.

The [^i^PrPPh_3_]^+^ and [MePPh_3_]^+^ cations in **1** and **2** are also well-known crystallographically [45,46] with P-C distances ranging from 1.795(2) to 1.882(2) Å (av. 1.803(2) Å) and 1.757(4) to 1.785(3) (*av*. 1.776(5)A), respectively. Likewise, the CPC angles about the (*sp^3^*) phosphorus centres range from 107.84(9) to 111.31(10)^o^ (*av*. 109.47(10)^o^) and 106.82(17) to 110.8(2)^o^ (*av.*109.47(19)^o^). These values are within previously observed ranges for these cations [45,46].

The closely related hydrated tetraphenylphosphonium pentaborate salt, [PPh_4_][B_5_O_6_(OH)_4_]·1.5H_2_O, has an interesting supramolecular giant structure composed of interpenetrating networks of complex H-bonded anion–anion interactions and cation–cation interactions involving multiple embraces of their aromatic rings [19]. Aromatic embraces are known to be strong stabilizing interactions [47,48] and are likely to be responsible (together with H-bonding interactions) for the crystallized self-assembly [28,29,30,31] of this compound. We examined the structures of **1** and **2** to see if similar aromatic interactions occur in these compounds, and details, together with their H-bonding interactions, are described below.

**Figure 4:**
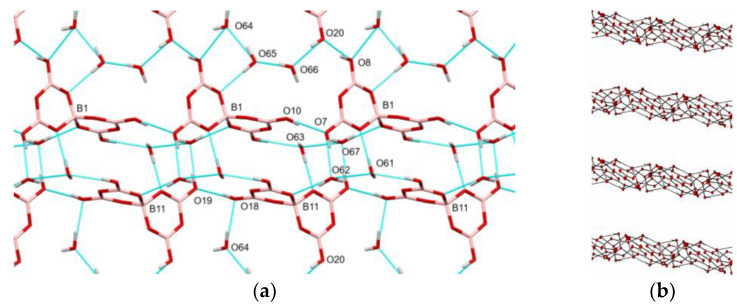
(**a**) A view along the *c* axis in compound **1** (perpendicular to a anionic pentaborate/H_2_O layer) with selected atomic labels, illustrating the two C(8) chains associated with the pentaborate(1-) anions containing B5 and B11. A third C_5_^5^(10) chain, involving three H_2_O molecules (containing O64, O65, and O66) and pentaborate(1-) oxygens (O8 and O20), can also be seen. (**b**) The pentaborate(1-) anion/H_2_O molecules in **1** can be viewed along the *b* axis, illustrating their layered structure.

Compound **1** is a co-crystallised phosphonium pentaborate salt with 3.5 H_2_O per cation/anion. These water molecules H-bond with the pentaborate(1-) anions and form a unique H_2_O/pentaborate H-bonded anionic network. Unusually for pentaborate salts, this anionic network is arranged in layers (Figure 4). Each pentaborate(1-) has four H-bond donor sites and the pentaborate anions containing B1 forms donor H-bonds to two β-sites (see Figure 1 for acceptor site labels [24]) of two neighbouring pentaborate anions (O10H10^…^O7′ and O8H8^…^O20″) and two H_2_O molecules (O7H7^…^O62 and O9H9^…^O63). The repeating O10-H-10^…^O7′ interaction is part of an infinite chain that links the B1 containing pentaborates(1-) anions. This interaction is C(8) using Etter terminology [49]. Likewise, the pentaborates containing B11 are similarly linked into infinite chains by C(8) interactions involving O18-H18^…^O19′. The other three H-bond acceptor sites for the pentaborate containing B11 are water molecules: O17H17^…^O61, O19H19^…^O67, and O20H20^…^O66. These two pentaborate chains are linked through a complex series of H-bonds involving four H_2_O molecules (containing O61, O62, O63 and O67) and a C_5_^5^(10) chain of three H_2_O molecules (containing O66, O65, O64), linking with O8H8-O20′ interborate interaction into layers (Figure 4).

**Figure 5:**
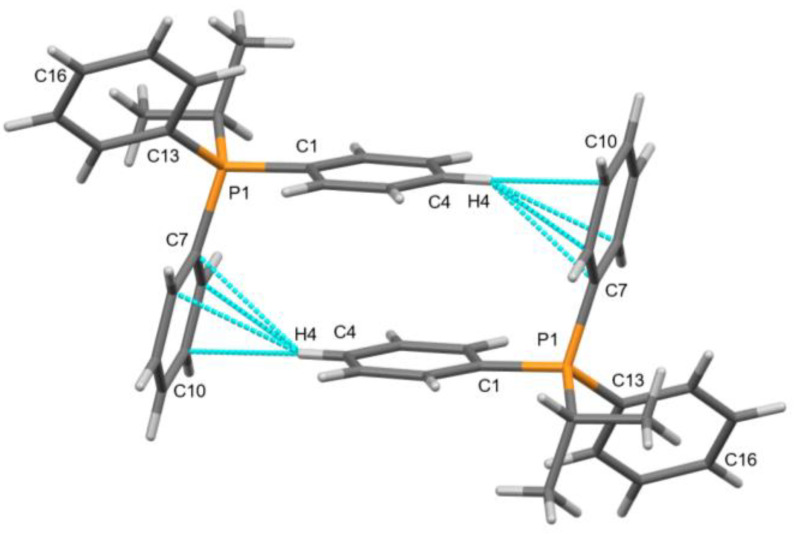
The centrosymmetric paired [^i^PrPPh_3_]^+^ cations (containing P1) in **1** display vertex-to-face interactions phenyl ring interactions, in addition to an offset face-to-face interactions (not highlighted). Similar interactions also occur in centrosymmetric paired cations containing P31.

The [^i^PrPPh_3_]^+^ cations in **1** are also arranged in layers, and these layers alternate with the anionic/H_2_O layers. Within these cationic layers, there are several cation–cation interactions involving their aromatic rings [47,48]. The two independent cations are both arranged as centrosymmetric pairs, with aromatic (phenyl) ring embrace interactions between each pair. Thus, the cation containing P1 forms an offset face-to-face (*off*) interaction between a phenyl ring (containing C1–C6) and the C1′–C6′ ring in its pair, with a centroid–centroid distance of 3.748(2) Å and a centroid-to-plane distance of 3.634(2) Å with a shift of 0.917(4) Å. These distances are indicative of a strong interaction and are considerably shorter than that found in [PPh_4_][B_5_O_6_(OH)_4_]·3/2H_2_O (ca. 4.3 Å) [19]. These phenyl rings are also involved in vertex-to-face (*vf*) interactions between C4H4 and its paired C7′–C12′ phenyl ring, with the H4-to-plane distance at 2.538(1) Å (Figure 5). Dance and Scudder refer to this type of interaction in tetraphenylphosphonium salts as a parallel quadruple phenyl embrace (PQPE) and calculate this interaction energy as −41 kJ.mol^−1^ [48]. Similarly, the cation containing P31 in **1** is also involved in a PQPE interaction with paired *off* interactions involving the C37–C42/C37′–C42′ phenyl rings and paired *vf* interactions from C40H40 to the phenyl ring containing C43′–C48′. For these rings, the centroid–centroid distance is 3.712(2) Å; the centroid-to-plane distance is 3.528(2) Å with a shift of 1.155(4) Å, and the H40-to-plane distance is 2.636(1) Å. The P1^…^P1 and P31^…^P31 distances are 8.004(1) Å and 8.170(1) Å, with P1^…^P31 of 11.129(1) Å. The [^i^PrPPh_3_]^+^ cations in **1** are oriented within the cationic layers with the ^i^Pr groups towards the pentaborate(1-)/H_2_O layers.

Compound **2** is a further example of a co-crystallized phosphonium pentaborate salt with one B(OH)_3_ and 0.5 (disordered) H_2_O per cation/anion. The supramolecular structure of **2** also displays anion–anion H-bond interactions and cation–cation aromatic embraces, but the details of these stabilizing interactions differ from those observed in **1** and [PPh_4_][B_5_O_6_(OH)_4_]·1.5H_2_O and are described below.

**Figure 6:**
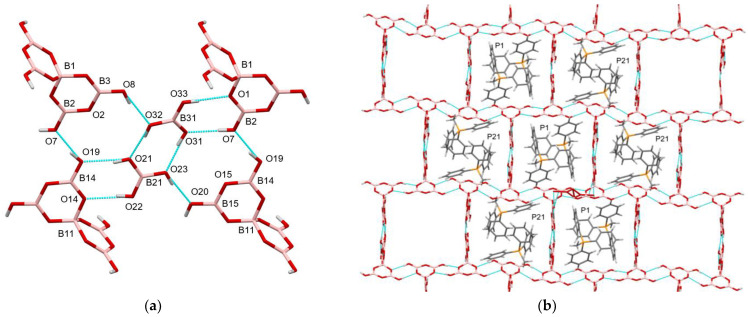
(**a**) View along the *c* axis in [MePPh_3_][B_5_O_6_(OH)_4_]·B(OH)_3_·0.5H_2_O (**2**) showing H-bond interactions involving the two B(OH)_3_ moieties and their three R_2_^2^(8) and the two R_4_^4^(12) ring motifs. (**b**) View along the *a* axis in **2,** illustrating the stacking pattern of the anionic network. The [MePPh_3_]^+^ cations (for clarity, only some are shown) occupy the channels as shown forming stacks (chains) with cations in the stacks only containing P atoms, as labelled. From this perspective, the B(OH)_3_ units are side-on and are in the ‘vertical’ section of the borate channels.

All hydroxyl groups of the two independent B(OH)_3_ and the two independent pentaborate(1-) anions are used as H-bond donor centres. The anion containing B1 forms two donor H-bonds to two α-sites (O9H9^…^O11′ and O10H10^…^O6′) of two adjacent pentaborates (one containing B11 and one containing B1) and both these interactions are R_2_^2^(8) [49] with the ring involving the O10H10 donor centrosymmetric (reciprocal). The anion containing B1 also forms two donor H-bonds to two adjacent B(OH)_3_ molecules: O8H8^…^O32, and O7′H7′^…^O31. The anion containing B11 forms three donor H-bonds to three adjacent anions at two α-sites (O17H17^…^O4′ and O18H18^…^O13′, reciprocal) and one β-site, (O19H19^…^O7′). This O10H19^…^O7′ interaction is part of a two larger R_4_^4^(12) ring interactions with both these rings including both B(OH)_3_ molecules (Figure 6). The fourth pentaborate donor interaction is to the disordered H_2_O (O20H20^…^O44), and overall the anion can be represented as α,α,β,ω [21]. The hydroxido groups of the two B(OH)_3_ molecules are arranged asymmetrically to maximise their acceptor/donor H-bond interactions. The B(OH)_3_ containing B31 forms a R_2_^2^(8) ‘pincer’ ring with the B(OH)_3_ containing B21, and likewise this B(OH)_3_ forms a ‘pincer’ R_2_^2^(8) interaction with the pentaborate containing B11 (Figure 6a). These interactions allow for the two co-crystallised B(OH)_3_ molecules to function as ‘spacer’ units to expand the lattice and replace what would otherwise be a simpler pentaborate/pentaborate R_2_^2^(8) interaction [18,33,34,35,36]. 

A view along the *a* axis of **2** (along the plane shown in Figure 6a) is shown in Figure 6b. This view reveals a stacked anionic lattice (rectangular and honeycomb-like) with channels that are occupied by the cations; interestingly, each cationic stack is occupied by either cations containing solely P1 or P21 and adjacent cationic stacks in the arrangement, as shown in Figure 6b. Cations are arranged as centrosymmetric pairs within the stacks with P1^…^P1 and P21^…^P21 distances of 6.253(2) Å and 6.255(2) Å, respectively. The repeating P⋯P distances in both stacks are 10.1076(10) Å, but the interpair dimer interactions differ. These interactions involve aromatic embraces and are *vf* (C19H19^…^C6′) and an edge-to face (*ef*) (C6H6^…^C11′, C7H7A^…^C10′) for P1-containing cations and *vf* (C39H39^…^C23′/C24′ and C24H24^…^C31′/C32′) for P21-containing cations. The closest contacts between centrosymmetric pairs arise from Me^…^Ph (C1H1B^…^C10′/C11′) and Ph^…^Ph (C3H3⋯C12′) interactions for the P1-containing stack whilst the P21-containing stack has an *off* phenyl ring interaction (between C34-C39 and C34′-C39′) with a centroid–centroid distance of 4.889(3) Å, a centroid-to-plane distance of 3.326(6) Å and a plane-to-plane shift of 3.583(7) Å. The C38-to-plane distance is 3.323(8) Å.

## 3. Materials and Experimental Methods

### 3.1. General

Reagents were all obtained commercially. FTIR spectra were obtained as KBr pellets on a Perkin-Elmer 100FTIR spectrometer (Perkin-Elmer, Seer Green, UK). ^1^H, ^11^B and ^13^C and ^31^P NMR spectra were obtained on a Bruker Avance-500 spectrometer (Bruker, Coventry, UK) on samples dissolved in D_2_O at 500, 160, 125 and 202 MHz, respectively. Chemical shifts are in ppm, with positive values to high frequency (downfield) of TMS (^1^H, ^13^C), BF_3_^.^OEt_2_ (^11^B) or H_3_PO_4_ (^31^P). TGA and DSC were performed on an SDT Q600 instrument (TA Instruments, New Castle, DE, USA) using Al_2_O_3_ crucibles with a temperature ramp-rate of 10 °C per minute (25 °C to 800 °C in air). BET measurements were performed on a Gemini 2375 analyser (Norcross, GA, USA) with N_2_ gas as the adsorbent. Samples were analysed between partial pressures (P/P_o_) of 0.05 and 0.30. X-ray crystallography was performed at the EPSRC national crystallography service centre at Southampton University. CHN analyses were obtained from OEA Laboratories (Callingham, Cornwall, UK).

### 3.2. X-ray Crystallography

Crystallographic data for **1** and **2** are given in the experimental section and in the Supplementary Data. Data collection for **1** and **2** was performed on a *Nonius KappaCCD* area detector (*ϕ* scans and *ω* scans to fill *asymmetric unit* sphere) diffractometer at 120(2)K. Unit cell parameters were determined using DirAx [50], with data collection using Collect [51]. Denzo [52] was used for data reduction and cell refinement and *SORTAV* [53,54] was used for absorption correction. *SHELXS97* [55] was used to solve the structure and was refined using *SHELXL 2018/3 97* [56]. *Olex2* [57] was used for graphics in the Supplementary Information.

### 3.3. Preparation of [^i^PrPPh_3_][B_5_O_6_(OH)_4_]^.^3.5H_2_O (**1**)

[^i^PrPPh_3_]I (3.0 g, 6.9 mmol) was dissolved in H_2_O (50 mL). To this solution, excess Dowex 550A monosphere (OH^−^ form) was added and the suspension was stirred for 24 h. The ion-exchange resin was removed by filtration and MeOH (50 mL) was added to the filtrate. B(OH)_3_ (2.14 g, 34.6 mmol) was added to the resulting solution, which was then heated for 1 h. The solvent was removed by rotary evaporation to yield a solid, which was dried at 110 °C for 24 h to give a white crude product (2.94 g, 73%). NMR. (δ^1^H/ppm): 1.30 (6*H*, dd, ^3^*J*(HH) 6.9, ^2^*J* (PH) 18.6 Hz), 3.73 (1*H*, dq, ^3^*J*(HH) 6.9 Hz), 7.50 *(*6*H*, m), 7.63 (9H, m); (δ(^13^C/ppm): 15.42 (2 × CH_3_), 20.09 (CH, d, ^1^*J*(CP) 48.9 Hz), 117.21 (3 × C, d, ^1^*J*(CP) 83.9 Hz), 130.04 (6 × CH, d, ^2^*J*(CP) 12.1 Hz), 133.62 (6 × CH, d, ^3^*J*(CP) 9.2 Hz), 134.78 (3 × CH, d, ^4^*J*(CP) 2.1 Hz); (δ ^11^B/ppm): 1.1, 12.9, 18.5; (δ ^31^P/ppm): 30.15. IR (KBr/cm^−1^); 3292 (vs), 3149 (vs), 1425 (vs), 1404 (vs), 1311 (vs), 1148 (m), 1106 (s), 1095 (s), 1061 (s), 1015 (s), 924 (s), 897 (s), 811 (m), 709 (s). Elemental analysis, C_21_H_33_B_5_O_13.5_P req. C 43.0%, H 5.7%, found C, 42.9%, H, 4.9%. TGA: 1st stage-loss of 5.5H_2_O (<275 °C): 17% expt., 17% calc., 2nd stage-oxidation (275–800 °C) to glassy residue: 36% expt., 30% calc. for 2.5 × B_2_O_3_. BET: multi-point surface area (m^2^/g) 0.7979 (**1**); 2.7599 (anhydrous); 0.2723 (pyrolysed). Crystals suitable for sc-XRD studies were obtained by crystallization from H_2_O. sc-XRD: C_21_H_33_B_5_O_13.5_P, *M_r_* = 586.49, triclinic, *P*−1, *a* = 9.1810(3) Å, *b* = 13.4812(5) Å, *c* = 23.8357(8) Å, *α* = 75.3130(10)°, *β* = 83.095(2)°, *γ* = 86.919(2)° *V* = 2832.24(17) Å^3^, *Z* = 4, *T* = 120(2) K, λ = 0.71073 Å, *D*_(calc.)_ 1.375 Mg/m, absorption coefficient 0.162 mm^−^^1^, *F(000)* 1228, 50,870 reflections measured, 99.7% complete to *θ* = 27.48°, 12,947 unique [*R_int_* = 0.0578], which were used in all calculations. The final *R1* = 0.0563 (*I* > 2σ(*I*)) and *wR2* = 0.1571 (all data).

### 3.4. Synthesis of [MePPh_3_][B_5_O_6_(OH)_4_]·B(OH)_3_·0.5H_2_O (**2**)

[MePPh_3_]I (2.50 g, 6.2 mmol) was dissolved in H_2_O (50 mL). To this solution, excess Dowex 550A monosphere (OH^−^ form) was added and the suspension was stirred for 24 h. The ion-exchange resin was removed by filtration and MeOH (50 mL) was added to the filtrate. B(OH)_3_ (1.91 g, 30.9 mmol) was added to the resulting solution, which was then heated for 1 h. The solvent was removed by rotary evaporation to yield an orange solid as the crude product, which was dried at 110 °C for 24 h (2.84 g, 98%). NMR. (δ^1^H/ppm): 2.69 (3H, d, ^2^*J*(HP) 13.8 Hz), 7.50 (12H, m), 7.68 (3H, m); (δ^13^C/ppm): 7.96 (CH_3_, d, ^1^*J(*CP) 58.5 Hz), 119.06 (C, d, ^1^*J(*CP) 89.2 Hz); (δ ^11^B/ppm) ppm: 1.2, 13.1, 18.6; (δ^31^P/ppm): 21.05. IR (KBr/cm^−1^): 3307 (s), 1417 (vs), 1296 (vs), 1118 (s), 1019 (m), 918 (s), 775 (m), 748 (m), 720 (m), 688 (m). Elemental analysis, C_19_H_26_B_6_O_13.5_P req. C 40.3%, H 4.6%, found C, 42.8%, H, 4.6%. TGA: 1st stage-loss of 4H_2_O (<275 °C): 12% expt., 13% calc., 2^nd^-stage oxidation (275–800 °C) to glassy residue: 42% expt., 37% calc. for 3 × B_2_O_3_. BET: multi-point surface area (m^2^/g) 0.5098 (**2**); 0.4134 (anhydrous); 0.2226 (pyrolysed). Crystals suitable for sc-XRD were obtained by crystallization from H_2_O. sc-XRD: C_38_H_52_B_12_O_27_P_2_, *M_r_* = 1132.46, triclinic, *P*−1, *a* = 10.1076(10) Å, *b* = 13.0403(10) Å, *c* = 20.6260(15) Å, *α* = 84.364(5)°, *β* = 82.811(4)°, *γ* = 76.492(4)°, *V* = 2615.9(4) Å^3^, *Z* = 2, *T* = 120(2) K, λ = 0.71073 Å, *D*_(calc.)_ 1.438 Mg /m, absorption coefficient 0.172 mm^−^^1^, *F(000)* 1172, 35,616 reflections measured, 98.8% complete to *θ* = 27.48°, 12,083 unique [*R_int_* = 0.0649], which were used in all calculations. The final *R1* = 0.0742 (*I* > 2σ(*I*)) and *wR2* = 0.1676 (all data).

## 4. Conclusions

Two substituted aryl phosphonium pentaborate salts were synthesized by templated crystallization from aqueous solution primed with B(OH_)3_ and appropriate aryl phosphonium cation and characterized by sc-XRD. Their structures are unusual for pentaborates in that [^i^PrPPh_3_][B_5_O_6_(OH)_4_]·3.5H_2_O has alternating layers of cations and anions whilst [MePPh_3_][B_5_O_6_(OH)_4_]·B(OH)_3_·0.5H_2_O has a rectangular honeycomb-like structure with cations stacked within channels. Despite their unusual structures, the materials derived by thermal oxidation of the cations are non-porous. The solid-state structures of both compounds are stabilized by multiple H-bonding and phenyl embrace interactions. 

## Data Availability

Crystallographic data available from CCDC, UK; see Supplementary Material for details.

## Supplementary Materials

The following supporting information can be downloaded at: https://www.mdpi.com/article/10.3390/molecules28196867/s1, Crystallographic data for **1** and **2** are available as Supplementary Materials. CCDC 2291682 (**1**) and 2291683 (**2**) also contain the supplementary crystallographic data for this paper. These CCDC data can be obtained free of charge via http://www.ccdc.cam.ac.uk/conts/retrieving.html (or from CCDC, 12 Union Road, Cambridge, CB2 1EZ. Fax: +44-1223-336033; E-mail: deposit@ccdc.cam.ac.uk).

## Sample Availability

Samples of the compounds are not available from the authors.
