# Supplementary material for: Synthesis and Thermal Studies of Two Phosphonium Tetrahydroxidohexaoxidopentaborate(1-) Salts: Single-Crystal XRD Characterization of [iPrPPh3][B5O6(OH)4]·3.5H2O and [MePPh3][B5O6(OH)4]·B(OH)3·0.5H2O
Source: Molecules. 2023 Sep 29;28(19):6867. doi: 10.3390/molecules28196867 (PMC10574587; doi:10.3390/molecules28196867)
Supplement: Supplementary file 1 [file molecules-28-06867-s001.zip › Electronic Supplementary data.pdf]

Electronic Supplementary data

Synthesis and thermal studies of two phosphonium tetrahydroxidohexaoxidopentaborate(1-) salts: single-crystal XRD characterization of $[^{i}PrPPh_3][B_5O_6(OH)_4]\cdot 3.5H_2O$ and $[MePPh_3][B_5O_6(OH)_4]\cdot B(OH)_3\cdot 0.5H_2O$

Michael A. Beckett [1*], Peter N. Horton[2], Michael B. Hursthouse,[b] and James L Timmis,[a]

[1] School of Natural Sciences, Bangor University, Bangor, Gwynedd, LL57 2UW, UK;
[2] Chemistry Department, University of Southampton, Southampton, SO17 1BJ, UK;
* Correspondence: m.a.beckett@bangor.ac..uk

Contains sc-XRD data and TGA data for $[^{i}PrPPh_3][B_5O_6(OH)_4]\cdot 3.5H_2O$ (**1**) and $[MePPh_3][B_5O_6(OH)_4]\cdot B(OH)_3\cdot 0.5H_2O$ (**2**)

[^{i}PrPPh$_3$][$B_5O_6(OH)_4$]·3.5H_2O Compound 1

R_1=7.42%

Submitted by: **Mike A Beckett**
Bangor University
Solved by: **Peter N Horton**
Sample ID: **MAB/JT/23**

Crystal Data and Experimental

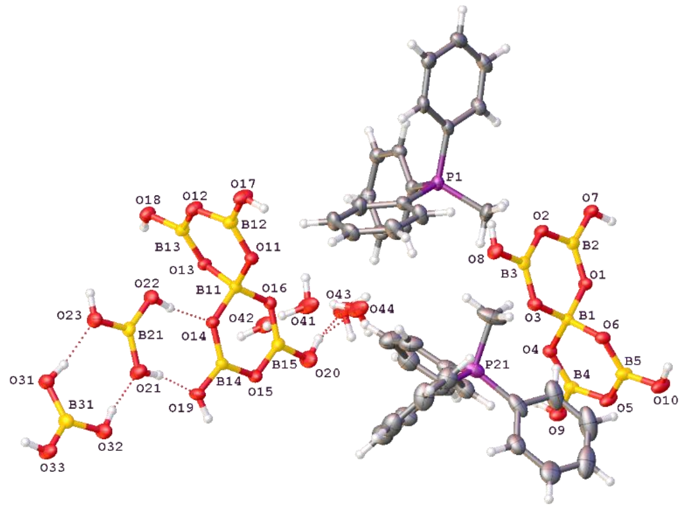

Experimental. Single colourless cut block-shaped crystals of **2009src0115** (**1**) were used as supplied. A suitable crystal with dimensions 0.200 × 0.130 × 0.070 mm^3 was selected and mounted on a Bruker-Nonius APEX II CCD camera on κ-goniostat diffractometer. The crystal was kept at a steady T = 120(2) K during data collection. The structure was solved with the ShelXS-97 (Sheldrick, 1997a) solution program using direct methods and by using Olex2 1.5-alpha (Dolomanov et al., 2009) as the graphical interface. The model was refined with ShelXL 2018/3 (Sheldrick, 2015) using full matrix least squares minimisation on $\boldsymbol{F}^2$.

Crystal Data. $C_{38}H_{52}B_{12}O_{27}P_2$, M_r = 1132.45, triclinic, P-1 (No. 2), a = 10.1076(10) Å, b = 13.0403(10) Å, c = 20.6260(15) Å, α = 84.364(5)°, β = 82.811(4)°, γ = 76.492(4)°, V = 2615.9(4) Å^3, T = 120(2) K, Z = 2, Z' = 1, μ(MoK$_\alpha$) = 0.172 mm^{-1}, 35616 reflections measured, 12083 unique (R_{int} = 0.0649) which were used in all calculations. The final wR_2 was 0.1676 (all data) and R_1 was 0.0742 (I≥2 σ(I)).

Compound 1 **2009src0115**

Formula	$C_{38}H_{52}B_{12}O_{27}P_2$
$D_{calc.}$/ g cm^{-3}	1.438
μ/mm^{-1}	0.172
Formula Weight	1132.45
Colour	colourless
Shape	cut block-shaped
Size/mm^3	0.200×0.130×0.070
T/K	120(2)
Crystal System	triclinic
Space Group	P-1
a/Å	10.1076(10)
b/Å	13.0403(10)
c/Å	20.6260(15)
α/°	84.364(5)
β/°	82.811(4)
γ/°	76.492(4)
V/Å^3	2615.9(4)
Z	2
Z'	1
Wavelength/Å	0.71073
Radiation type	MoK$_\alpha$
Θ_{min}/°	2.994
Θ_{max}/°	27.938
Measured Refl's.	35616
Indep't Refl's	12083
Refl's I≥2 σ(I)	9025
R_{int}	0.0649
Parameters	735
Restraints	1
Largest Peak	1.103
Deepest Hole	-1.031
GooF	1.040
wR_2 (all data)	0.1676
wR_2	0.1485
R_1 (all data)	0.1044
R_1	0.0742

Structure Quality Indicators

**Reflections:**	d min (MoKα) 2Θ=55.9° 0.76	I/σ(I) 15.0	Rint m=2.95 6.49%	Full 50.5° 96% to 55.9° 98.8
**Refinement:**	Shift 0.000	Max Peak 1.1	Min Peak -1.0	GooF 1.040

A colourless cut block-shaped-shaped crystal with dimensions 0.200 × 0.130 × 0.070 mm^3 was mounted. Data were collected using a Bruker-Nonius APEX II CCD camera on κ-goniostat diffractometer equipped with an Oxford Cryosystems low-temperature device operating at T = 120(2) K.

Data were measured using ϕ & ω scans with MoK$_\alpha$ radiation. The diffraction pattern was indexed and the total number of runs and images was based on the strategy calculation from the program COLLECT (Hooft, R.W.W., 1998). The maximum resolution that was achieved was Θ = 27.938° (0.76 Å).

The unit cell was refined using DENZO (Otwinowski & Minor, 1997) & COLLECT (Hooft, R.W.W., 1998) on 1919 reflections, 5% of the observed reflections.

Data reduction, scaling and absorption corrections were performed using DENZO (Otwinowski & Minor, 1997) & COLLECT (Hooft, R.W.W., 1998). The final completeness is 98.80 % out to 27.938° in Θ. SADABS

2007/2 (Sheldrick, G.M., 2007). The absorption coefficient μ of this material is 0.172 mm^{-1} at this wavelength (λ = 0.71073Å) and the minimum and maximum transmissions are 0.967 and 0.988.

The structure was solved and the space group *P*-1 (# 2) determined by the ShelXS-97 (Sheldrick, 1997a) structure solution program using using direct methods and refined by full matrix least squares minimisation on $\boldsymbol{F^2}$ using version 2018/3 of ShelXL 2018/3 (Sheldrick, 2015). All non-hydrogen atoms were refined anisotropically. Hydrogen atom positions were calculated geometrically and refined using the riding model.

_refine_special_details: There was a disordered water molecule over 4 sites (and symmetry related) for which some constraints were applied.

_exptl_absorpt_process_details: SADABS 2007/2 (Sheldrick, G.M., 2007)

There is a single formula unit in the asymmetric unit, which is represented by the reported sum formula. In other words: Z is 2 and Z' is 1. The moiety formula is 2(B5 H4 O10), 2(C19 H18 P), 2(B H3 O3), H2 O.

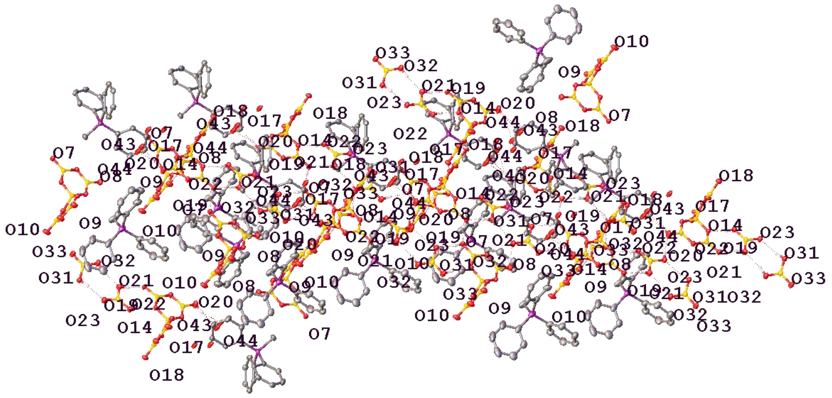

Figure S1 The following hydrogen bonding interactions with a maximum D-D distance of 2.9 Å and a minimum angle of 120 ° are present in **2009src0115**: O7–O31_1: 2.714 Å, O8–O32_2: 2.748 Å, O9–O11_3: 2.828 Å, O10–O6_4: 2.803 Å, O17–O4_5: 2.779 Å, O18–O13_6: 2.764 Å, O19–O7_7: 2.673 Å, O20–O44: 2.622 Å, O20–O43: 2.676 Å, O20–O42_8: 3.057 Å, O21–O19: 2.676 Å, O22–O14: 2.695 Å, O23–O20_5: 2.71 Å, O31–O23: 2.699 Å, O32–O21: 2.671 Å, O33–O1_9: 2.696 Å.

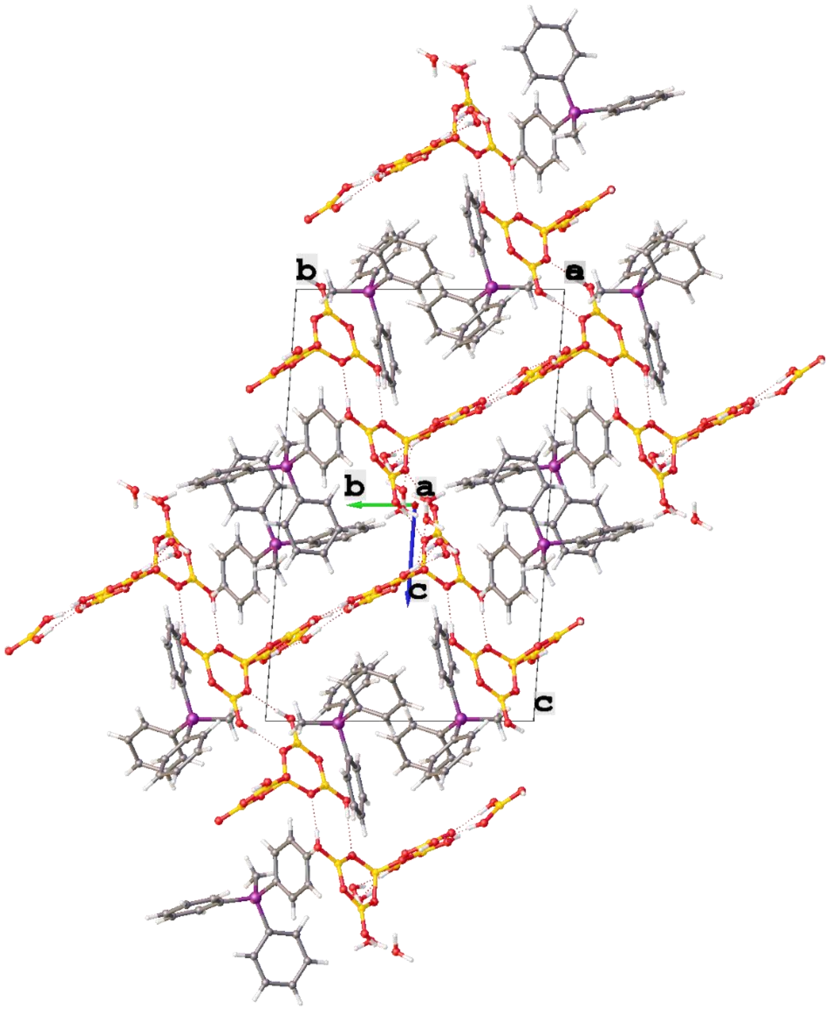

Figure S2 Packing diagram of 2009src0115 viewed along the a axis

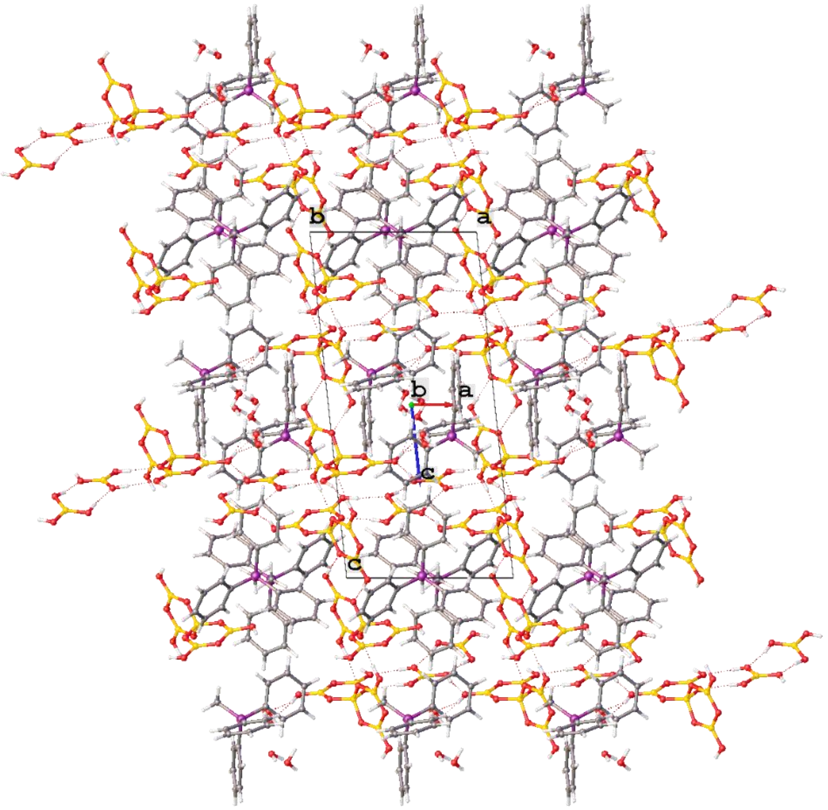

Figure S3 Packing diagram of 2009src0115 viewed along the b axis

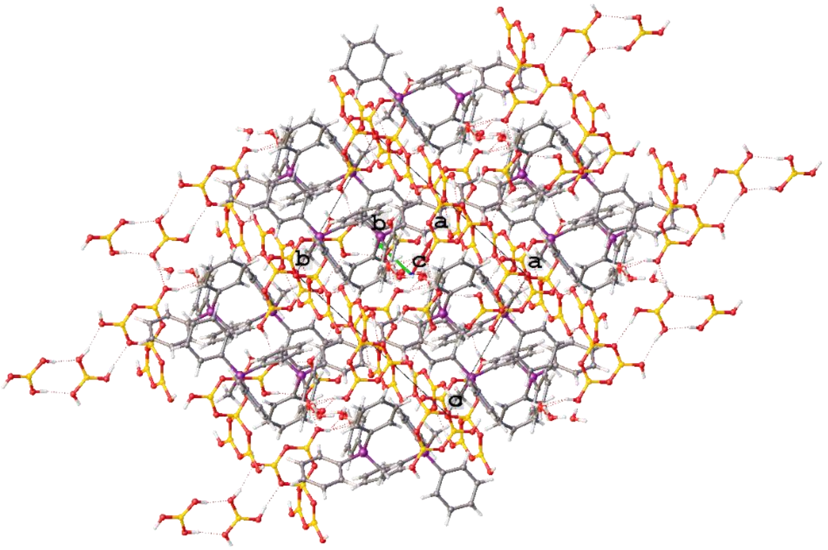

Figure S4 Packing diagram of 2009src0115 viewed along the c axis

Table S1: Fractional Atomic Coordinates (×10[4]) and Equivalent Isotropic Displacement Parameters (Å^2×10^3) for **2009src0115**. U_{eq} is defined as 1/3 of the trace of the orthogonalised U_{ij}.

Atom	x	y	z	U_{eq}
B1	724(3)	9085(2)	1353.6(16)	18.1(6)
B2	496(3)	10658(3)	1971.2(15)	18.8(6)
B3	2684(3)	9788(3)	1527.8(15)	18.7(6)
B4	-437(4)	7598(3)	1487.6(16)	20.6(7)
B5	-654(4)	8746(3)	515.1(17)	24.0(7)
O1	-71(2)	9965.8(15)	1734.1(10)	19.8(4)
O2	1879(2)	10587.2(16)	1901.3(10)	21.3(4)
O3	2159(2)	9067.2(16)	1296.2(10)	20.5(4)
O4	449(2)	8100.0(15)	1688.8(10)	20.1(4)
O5	-1000(2)	7903.4(18)	899.8(10)	28.4(5)
O6	255(2)	9249.4(16)	696.4(9)	20.9(4)
O7	-280(2)	11473.3(17)	2308.9(11)	24.3(5)
O8	4030(2)	9755.8(18)	1413.1(11)	26.6(5)
O9	-835(2)	6768.2(17)	1843.6(11)	27.7(5)
O10	-1242(3)	9043.2(19)	-48.7(11)	32.2(5)
B11	9639(3)	5489(3)	3504.2(16)	18.5(6)
B12	10908(4)	6910(3)	3301.9(16)	21.7(7)
B13	10940(3)	5921(3)	4339.0(16)	20.2(7)
B14	9933(3)	3771(2)	3023.0(15)	17.5(6)
B15	7713(4)	4750(3)	3319.3(17)	22.6(7)
O11	10005(2)	6405.1(15)	3119.3(10)	20.1(4)
O12	11450(2)	6637.1(17)	3899.9(10)	25.8(5)
O13	9963(2)	5459.6(15)	4187.4(9)	18.9(4)
O14	10474(2)	4526.3(15)	3216.5(10)	18.6(4)
O15	8558(2)	3867.3(16)	3051.5(11)	23.1(4)
O16	8209(2)	5524.2(15)	3509.5(10)	19.4(4)
O17	11338(3)	7704.6(18)	2929.6(11)	30.8(5)
O18	11464(2)	5726.8(17)	4927.1(10)	24.4(5)
O19	10787(2)	2883.1(16)	2801.5(11)	23.1(4)
O20	6372(2)	4753.9(19)	3372.3(14)	38.8(6)
C1	1443(3)	9935(3)	3597.2(16)	31.0(7)
C2	2938(3)	11214(2)	4188.1(14)	21.3(6)
C3	1791(3)	11967(2)	4383.6(16)	27.8(7)
C4	1920(4)	12954(3)	4517.2(16)	32.9(8)
C5	3181(4)	13189(3)	4458.9(16)	30.5(7)
C6	4316(3)	12459(3)	4255.3(15)	28.2(7)
C7	4201(3)	11462(3)	4117.4(15)	25.8(7)
C8	2416(3)	9300(2)	4887.8(14)	21.6(6)
C9	2448(3)	9808(2)	5448.3(15)	22.4(6)
C10	2229(3)	9302(2)	6060.4(15)	24.8(6)
C11	1996(3)	8290(3)	6112.9(15)	24.9(6)
C12	1956(3)	7787(3)	5558.7(16)	26.4(7)
C13	2159(3)	8287(2)	4942.0(15)	25.8(6)
C14	4329(3)	9167(2)	3719.8(15)	23.2(6)
C15	4619(4)	9291(3)	3045.9(16)	32.9(8)
C16	5833(4)	8745(3)	2753.0(17)	37.8(9)
C17	6762(4)	8091(3)	3132.7(17)	33.1(8)
C18	6479(3)	7969(3)	3792.9(17)	32.9(8)
C19	5266(3)	8504(3)	4091.0(16)	29.4(7)
P1	2782.9(8)	9903.2(6)	4091.9(4)	21.23(17)
C21	5479(5)	8645(3)	43(2)	53.4(12)
C22	4182(4)	7103(3)	-416.9(18)	39.3(9)
C23	4480(4)	6349(3)	-883.1(18)	37.1(8)
C24	3499(4)	6210(3)	-1252(2)	46.2(10)
C25	2200(5)	6820(4)	-1160(2)	63.1(15)
C26	1896(5)	7569(4)	-700(2)	67.0(15)
C27	2868(4)	7719(4)	-329(2)	54.4(12)
C28	5150(4)	6788(3)	878.3(17)	33.1(8)
C29	3940(4)	6475(3)	1094.6(18)	34.7(8)

Atom	x	y	z	U_{eq}
C30	3696(4)	6175(3)	1750.2(19)	40.7(9)
C31	4630(4)	6184(3)	2178(2)	43.9(9)
C32	5839(4)	6477(3)	1959.1(19)	43.9(9)
C33	6090(4)	6797(3)	1305.9(19)	40.7(9)
C34	7034(4)	6566(3)	-307.2(17)	31.9(7)
C35	7682(4)	7027(3)	-852.6(18)	40.5(9)
C36	8805(4)	6438(4)	-1178.9(19)	49.8(11)
C37	9298(4)	5397(4)	-977.0(19)	45.8(10)
C38	8674(4)	4934(3)	-434(2)	42.7(9)
C39	7537(4)	5518(3)	-98.7(19)	38.5(9)
P21	5461.1(10)	7279.1(7)	50.5(5)	33.9(2)
B21	13973(3)	3538(3)	2828.6(17)	21.4(7)
O21	13483(2)	2766.1(18)	2592.5(12)	30.1(5)
O22	13187(2)	4426.4(16)	3070.1(11)	24.2(5)
O23	15345(2)	3365.8(17)	2800.9(11)	26.9(5)
B31	16446(4)	1037(3)	1954.3(18)	25.0(7)
O31	16996(2)	1576.6(17)	2357.6(11)	26.5(5)
O32	15094(2)	1259.1(18)	1881.6(12)	30.3(5)
O33	17238(2)	252.4(19)	1616.4(12)	33.8(6)
O42	5273(10)	4515(7)	5343(3)	42.6(17)
O41	4373(11)	5477(8)	5093(3)	42.6(17)
O43	4513(4)	6026(4)	4149(3)	42.6(17)
O44	4456(5)	6289(4)	3863(3)	42.6(17)

Table S2: Anisotropic Displacement Parameters (×10[4]) for **2009src0115**. The anisotropic displacement factor exponent takes the form: $-2\pi^2[h^2a^{*2} \times U_{11} + ... + 2hka^* \times b^* \times U_{12}]$

Atom	U_{11}	U_{22}	U_{33}	U_{23}	U_{13}	U_{12}
B1	19.8(16)	15.8(15)	19.2(15)	-2.2(12)	-2.1(12)	-4.5(12)
B2	20.0(16)	19.5(15)	16.8(15)	-1.4(12)	-1.5(12)	-4.3(12)
B3	19.4(16)	20.6(15)	15.8(14)	1.7(12)	-1.0(12)	-5.7(12)
B4	24.6(17)	19.5(16)	17.8(15)	-2.1(13)	1.8(12)	-7.0(13)
B5	31.6(19)	23.2(17)	19.8(16)	-2.0(14)	-1.7(14)	-11.9(14)
O1	16.7(10)	20.2(10)	22.8(10)	-6.3(8)	0.0(8)	-4.0(8)
O2	17.6(10)	23.9(10)	24.6(10)	-6.4(9)	-2.8(8)	-6.9(8)
O3	17.9(10)	20.5(10)	23.2(10)	-5.1(8)	1.9(8)	-5.2(8)
O4	21.9(10)	18.8(10)	20.2(10)	-1.3(8)	-1.0(8)	-6.4(8)
O5	37.3(13)	30.5(12)	24.7(11)	3.6(9)	-9.1(9)	-21.8(10)
O6	25.5(11)	19.9(10)	18.5(10)	-2.1(8)	-0.5(8)	-8.5(8)
O7	17.9(10)	26.6(11)	30.0(11)	-11.2(9)	-3.0(9)	-4.4(9)
O8	17.9(11)	33.6(12)	29.9(12)	-6.5(10)	0.5(9)	-9.0(9)
O9	35.9(13)	26.8(11)	25.1(11)	2.2(9)	-4.7(10)	-17.2(10)
O10	47.0(15)	33.5(13)	24.2(11)	4.6(10)	-12.5(10)	-23.7(11)
B11	19.9(16)	17.2(15)	18.4(15)	-0.1(12)	-3.0(12)	-4.3(12)
B12	24.2(17)	24.1(17)	18.3(15)	-3.2(13)	-1.3(13)	-8.3(13)
B13	21.3(16)	21.6(16)	18.4(15)	-3.8(13)	-0.6(12)	-6.2(13)
B14	18.6(15)	16.9(15)	16.9(14)	-1.8(12)	-1.5(12)	-3.5(12)
B15	20.9(17)	22.5(16)	25.8(17)	-0.7(14)	-5.3(13)	-6.2(13)
O11	22.0(11)	18.2(10)	20.7(10)	-0.1(8)	-3.0(8)	-5.9(8)
O12	28.2(12)	32.8(12)	21.1(10)	-1.1(9)	-3.1(9)	-16.4(10)
O13	22.5(10)	18.0(10)	17.7(9)	-1.5(8)	-2.7(8)	-7.3(8)
O14	16.1(10)	18.1(10)	22.5(10)	-4.9(8)	-1.4(8)	-4.6(8)
O15	16.7(10)	19.6(10)	34.5(12)	-5.7(9)	-4.1(9)	-4.6(8)
O16	15.9(10)	17.7(10)	24.4(10)	-3.6(8)	-1.7(8)	-2.5(8)
O17	43.3(14)	33.8(12)	22.3(11)	1.2(10)	-4.3(10)	-23.8(11)
O18	27.4(12)	27.8(12)	21.1(10)	-0.4(9)	-4.6(9)	-12.1(9)
O19	20.6(11)	19.5(10)	30.2(11)	-9.9(9)	1.2(9)	-5.1(8)
O20	16.6(11)	34.5(14)	66.6(18)	-14.6(13)	-4.3(11)	-3.8(10)
C1	24.2(16)	38.5(19)	30.3(17)	-2.4(15)	-10.5(13)	-3.1(14)
C2	21.6(15)	21.0(14)	18.1(13)	1.0(11)	-2.2(11)	0.6(11)

Atom	U_{11}	U_{22}	U_{33}	U_{23}	U_{13}	U_{12}
C3	25.7(16)	24.6(16)	29.7(17)	2.4(13)	-0.4(13)	-1.6(13)
C4	34.8(19)	25.7(16)	29.6(17)	-0.2(14)	3.2(14)	6.5(14)
C5	42(2)	21.9(16)	25.9(16)	-0.5(13)	-4.7(14)	-3.8(14)
C6	30.2(17)	30.3(17)	24.7(16)	3.7(13)	-6.9(13)	-8.3(14)
C7	20.7(15)	28.4(16)	24.5(15)	-0.7(13)	-3.9(12)	2.5(12)
C8	17.7(14)	23.3(15)	22.2(14)	-1.0(12)	-1.3(11)	-2.1(11)
C9	21.0(15)	20.2(14)	25.6(15)	-4.1(12)	0.8(12)	-4.3(11)
C10	23.7(15)	26.9(16)	23.5(15)	-3.7(13)	-0.2(12)	-5.4(12)
C11	19.9(15)	30.3(16)	23.3(15)	0.8(13)	1.7(12)	-6.6(12)
C12	23.1(16)	24.5(15)	31.9(17)	-1.1(13)	1.0(13)	-8.3(12)
C13	24.7(16)	28.5(16)	24.6(15)	-8.1(13)	0.8(12)	-6.1(13)
C14	23.8(15)	21.3(14)	23.2(15)	-3.7(12)	-0.5(12)	-2.7(12)
C15	33.5(18)	31.9(18)	25.3(16)	1.5(14)	1.8(14)	4.6(14)
C16	41(2)	35.9(19)	27.3(17)	-2.3(15)	7.2(15)	3.9(16)
C17	30.4(18)	27.7(17)	35.4(18)	-7.7(14)	6.1(14)	2.4(14)
C18	27.2(17)	33.3(18)	32.9(18)	-4.0(15)	-4.9(14)	5.6(14)
C19	26.4(17)	36.1(18)	22.7(15)	-4.0(14)	-3.3(13)	0.0(14)
P1	18.8(4)	23.6(4)	19.7(4)	-1.8(3)	-2.1(3)	-1.5(3)
C21	80(3)	25.0(18)	51(2)	-8.4(18)	26(2)	-19(2)
C22	40(2)	28.1(18)	37.0(19)	12.3(15)	14.6(16)	2.5(15)
C23	33.5(19)	31.4(18)	42(2)	8.6(16)	3.8(16)	-7.6(15)
C24	50(2)	45(2)	43(2)	18.6(19)	-2.0(18)	-19.1(19)
C25	41(2)	90(4)	51(3)	39(3)	-8(2)	-18(2)
C26	39(3)	84(4)	55(3)	25(3)	10(2)	13(2)
C27	45(2)	62(3)	38(2)	16(2)	7.5(18)	9(2)
C28	40(2)	24.6(16)	31.7(18)	-2.4(14)	12.0(15)	-10.0(14)
C29	31.0(18)	32.4(18)	36.2(19)	1.2(15)	7.3(15)	-5.5(14)
C30	34(2)	40(2)	41(2)	8.8(17)	11.9(16)	-7.5(16)
C31	45(2)	44(2)	39(2)	0.2(18)	7.4(17)	-9.7(18)
C32	55(3)	46(2)	37(2)	-4.8(18)	-0.9(18)	-24.7(19)
C33	49(2)	36(2)	41(2)	-10.3(17)	10.9(17)	-21.9(17)
C34	35.8(19)	30.4(17)	30.6(17)	-6.9(14)	8.1(14)	-13.8(15)
C35	38(2)	50(2)	31.4(19)	4.8(17)	3.7(15)	-14.7(17)
C36	36(2)	77(3)	30.3(19)	9(2)	8.1(16)	-11(2)
C37	34(2)	66(3)	34(2)	-11.5(19)	4.0(16)	-6.8(19)
C38	35(2)	39(2)	53(2)	-7.0(18)	2.4(17)	-7.9(16)
C39	40(2)	33.1(19)	40(2)	-0.2(16)	10.3(16)	-13.0(16)
P21	42.3(5)	22.6(4)	32.7(5)	-1.0(4)	12.9(4)	-8.4(4)
B21	14.5(15)	23.9(16)	25.7(17)	-0.1(14)	-1.2(12)	-5.2(13)
O21	20.8(11)	34.5(13)	37.8(13)	-12.3(10)	-1.8(10)	-8.3(10)
O22	15.7(10)	25.2(11)	32.0(12)	-5.0(9)	0.5(9)	-5.8(8)
O23	16.0(10)	29.3(12)	35.2(13)	-7.5(10)	0.2(9)	-3.8(9)
B31	19.3(17)	26.2(18)	29.2(18)	-3.6(15)	0.8(14)	-5.5(13)
O31	20.7(11)	27.0(12)	31.7(12)	-8.8(10)	-0.8(9)	-3.3(9)
O32	17.5(11)	29.0(12)	44.3(14)	-11.8(10)	1.0(10)	-3.7(9)
O33	17.3(11)	38.0(13)	48.8(15)	-21.2(11)	-0.5(10)	-5.4(10)
O42	16(2)	51(3)	63(5)	-36(3)	4(3)	-3(2)
O41	16(2)	51(3)	63(5)	-36(3)	4(3)	-3(2)
O43	16(2)	51(3)	63(5)	-36(3)	4(3)	-3(2)
O44	16(2)	51(3)	63(5)	-36(3)	4(3)	-3(2)

Table S3: Bond Lengths in Å for **2009src0115**.

Atom	Atom	Length/Å
B1	O1	1.473(4)
B1	O3	1.435(4)
B1	O4	1.466(4)
B1	O6	1.471(4)
B2	O1	1.335(4)
B2	O2	1.369(4)

Atom	Atom	Length/Å
B2	O7	1.360(4)
B3	O2	1.395(4)
B3	O3	1.334(4)
B3	O8	1.342(4)
B4	O4	1.350(4)
B4	O5	1.387(4)

Atom	Atom	Length/Å
B4	O9	1.359(4)
B5	O5	1.378(4)
B5	O6	1.353(4)
B5	O10	1.353(4)
B11	O11	1.463(4)
B11	O13	1.481(4)
B11	O14	1.472(4)
B11	O16	1.434(4)
B12	O11	1.351(4)
B12	O12	1.393(4)
B12	O17	1.350(4)
B13	O12	1.378(4)
B13	O13	1.351(4)
B13	O18	1.361(4)
B14	O14	1.346(4)
B14	O15	1.361(4)
B14	O19	1.354(4)
B15	O15	1.381(4)
B15	O16	1.337(4)
B15	O20	1.346(4)
C1	P1	1.785(3)
C2	C3	1.379(4)
C2	C7	1.375(4)
C2	P1	1.785(3)
C3	C4	1.380(5)
C4	C5	1.368(5)
C5	C6	1.359(5)
C6	C7	1.392(5)
C8	C9	1.395(4)
C8	C13	1.396(4)
C8	P1	1.784(3)
C9	C10	1.383(4)
C10	C11	1.386(4)
C11	C12	1.382(4)
C12	C13	1.386(4)

Atom	Atom	Length/Å
C14	C15	1.386(4)
C14	C19	1.378(4)
C14	P1	1.760(3)
C15	C16	1.367(5)
C16	C17	1.378(5)
C17	C18	1.357(5)
C18	C19	1.365(4)
C21	P21	1.784(4)
C22	C23	1.399(5)
C22	C27	1.382(5)
C22	P21	1.774(5)
C23	C24	1.376(6)
C24	C25	1.368(6)
C25	C26	1.385(8)
C26	C27	1.377(7)
C28	C29	1.386(5)
C28	C33	1.378(5)
C28	P21	1.780(3)
C29	C30	1.379(5)
C30	C31	1.373(6)
C31	C32	1.375(5)
C32	C33	1.381(5)
C34	C35	1.387(5)
C34	C39	1.387(5)
C34	P21	1.757(4)
C35	C36	1.355(5)
C36	C37	1.374(6)
C37	C38	1.373(5)
C38	C39	1.369(5)
B21	O21	1.373(4)
B21	O22	1.344(4)
B21	O23	1.347(4)
B31	O31	1.382(4)
B31	O32	1.354(4)
B31	O33	1.339(4)

Table S4: Bond Angles in ° for **2009src0115**.

Atom	Atom	Atom	Angle/°
O3	B1	O1	110.9(2)
O3	B1	O4	110.7(2)
O3	B1	O6	109.2(2)
O4	B1	O1	107.8(2)
O4	B1	O6	110.7(2)
O6	B1	O1	107.5(2)
O1	B2	O2	122.7(3)
O1	B2	O7	121.3(3)
O7	B2	O2	116.0(3)
O3	B3	O2	122.1(3)
O3	B3	O8	120.3(3)
O8	B3	O2	117.6(3)
O4	B4	O5	121.7(3)
O4	B4	O9	122.6(3)
O9	B4	O5	115.8(3)
O6	B5	O5	120.6(3)
O6	B5	O10	121.5(3)
O10	B5	O5	117.9(3)
B2	O1	B1	123.2(2)
B2	O2	B3	116.7(2)
B3	O3	B1	124.1(2)

Atom	Atom	Atom	Angle/°
B4	O4	B1	122.3(2)
B5	O5	B4	119.0(3)
B5	O6	B1	123.1(2)
O11	B11	O13	110.4(2)
O11	B11	O14	108.2(2)
O14	B11	O13	107.7(2)
O16	B11	O11	110.8(2)
O16	B11	O13	109.0(2)
O16	B11	O14	110.7(2)
O11	B12	O12	121.1(3)
O17	B12	O11	123.2(3)
O17	B12	O12	115.7(3)
O13	B13	O12	121.0(3)
O13	B13	O18	122.9(3)
O18	B13	O12	116.1(3)
O14	B14	O15	122.1(3)
O14	B14	O19	118.8(3)
O19	B14	O15	119.1(3)
O16	B15	O15	121.9(3)
O16	B15	O20	123.7(3)
O20	B15	O15	114.3(3)

Atom	Atom	Atom	Angle/°
B12	O11	B11	122.3(2)
B13	O12	B12	119.0(2)
B13	O13	B11	121.6(2)
B14	O14	B11	123.1(2)
B14	O15	B15	117.8(2)
B15	O16	B11	124.2(2)
C3	C2	P1	119.6(2)
C7	C2	C3	119.4(3)
C7	C2	P1	120.8(2)
C2	C3	C4	119.9(3)
C5	C4	C3	120.4(3)
C6	C5	C4	120.1(3)
C5	C6	C7	120.0(3)
C2	C7	C6	120.0(3)
C9	C8	C13	120.3(3)
C9	C8	P1	120.8(2)
C13	C8	P1	118.8(2)
C10	C9	C8	119.8(3)
C9	C10	C11	119.8(3)
C12	C11	C10	120.6(3)
C11	C12	C13	120.3(3)
C12	C13	C8	119.2(3)
C15	C14	P1	119.0(2)
C19	C14	C15	120.1(3)
C19	C14	P1	120.9(2)
C16	C15	C14	119.5(3)
C15	C16	C17	119.6(3)
C18	C17	C16	120.9(3)
C17	C18	C19	120.1(3)
C18	C19	C14	119.8(3)
C2	P1	C1	110.34(15)
C8	P1	C1	110.33(15)
C8	P1	C2	107.74(14)
C14	P1	C1	109.16(16)
C14	P1	C2	110.16(14)
C14	P1	C8	109.09(14)

Atom	Atom	Atom	Angle/°
C23	C22	P21	121.0(3)
C27	C22	C23	118.7(4)
C27	C22	P21	120.3(3)
C24	C23	C22	121.5(4)
C25	C24	C23	119.3(5)
C24	C25	C26	119.5(5)
C27	C26	C25	121.8(4)
C26	C27	C22	119.1(5)
C29	C28	P21	120.3(3)
C33	C28	C29	121.0(3)
C33	C28	P21	118.5(3)
C30	C29	C28	118.4(4)
C31	C30	C29	120.8(4)
C30	C31	C32	120.7(4)
C31	C32	C33	119.3(4)
C28	C33	C32	119.8(4)
C35	C34	C39	120.3(3)
C35	C34	P21	118.6(3)
C39	C34	P21	120.6(3)
C36	C35	C34	119.0(4)
C35	C36	C37	120.8(4)
C38	C37	C36	120.7(4)
C39	C38	C37	119.2(4)
C38	C39	C34	120.0(3)
C22	P21	C21	110.8(2)
C22	P21	C28	110.34(17)
C28	P21	C21	108.37(18)
C34	P21	C21	110.39(19)
C34	P21	C22	106.82(17)
C34	P21	C28	110.10(17)
O22	B21	O21	124.7(3)
O22	B21	O23	119.7(3)
O23	B21	O21	115.6(3)
O32	B31	O31	122.6(3)
O33	B31	O31	121.0(3)
O33	B31	O32	116.4(3)

Table S5: Torsion Angles in ° for **2009src0115**.

Atom	Atom	Atom	Atom	Angle/°
O1	B1	O3	B3	-3.3(4)
O1	B1	O4	B4	100.5(3)
O1	B1	O6	B5	-99.0(3)
O1	B2	O2	B3	4.2(4)
O2	B2	O1	B1	-2.4(4)
O2	B3	O3	B1	5.7(4)
O3	B1	O1	B2	1.7(4)
O3	B1	O4	B4	-138.0(3)
O3	B1	O6	B5	140.6(3)
O3	B3	O2	B2	-5.7(4)
O4	B1	O1	B2	123.1(3)
O4	B1	O3	B3	-123.0(3)
O4	B1	O6	B5	18.5(4)
O4	B4	O5	B5	-0.6(5)
O5	B4	O4	B1	9.0(4)
O5	B5	O6	B1	-12.2(5)
O6	B1	O1	B2	-117.6(3)
O6	B1	O3	B3	114.9(3)
O6	B1	O4	B4	-16.8(4)
O6	B5	O5	B4	2.1(5)

Atom	Atom	Atom	Atom	Angle/˚
O7	B2	O1	B1	178.6(3)
O7	B2	O2	B3	-176.8(2)
O8	B3	O2	B2	175.2(3)
O8	B3	O3	B1	-175.3(3)
O9	B4	O4	B1	-171.0(3)
O9	B4	O5	B5	179.4(3)
O10	B5	O5	B4	-178.5(3)
O10	B5	O6	B1	168.5(3)
O11	B11	O13	B13	-25.2(4)
O11	B11	O14	B14	-125.0(3)
O11	B11	O16	B15	124.3(3)
O11	B12	O12	B13	-7.1(4)
O12	B12	O11	B11	-5.6(4)
O12	B13	O13	B11	15.1(4)
O13	B11	O11	B12	20.4(4)
O13	B11	O14	B14	115.8(3)
O13	B11	O16	B15	-114.1(3)
O13	B13	O12	B12	2.3(4)
O14	B11	O11	B12	-97.1(3)
O14	B11	O13	B13	92.7(3)
O14	B11	O16	B15	4.2(4)
O14	B14	O15	B15	-3.3(4)
O15	B14	O14	B11	3.2(4)
O15	B15	O16	B11	-4.9(5)
O16	B11	O11	B12	141.3(3)
O16	B11	O13	B13	-147.1(3)
O16	B11	O14	B14	-3.3(4)
O16	B15	O15	B14	4.1(4)
O17	B12	O11	B11	174.8(3)
O17	B12	O12	B13	172.5(3)
O18	B13	O12	B12	-176.8(3)
O18	B13	O13	B11	-165.8(3)
O19	B14	O14	B11	-176.0(2)
O19	B14	O15	B15	175.8(3)
O20	B15	O15	B14	-175.6(3)
O20	B15	O16	B11	174.8(3)
C2	C3	C4	C5	-0.2(5)
C3	C2	C7	C6	1.3(5)
C3	C2	P1	C1	50.2(3)
C3	C2	P1	C8	-70.3(3)
C3	C2	P1	C14	170.8(2)
C3	C4	C5	C6	1.4(5)
C4	C5	C6	C7	-1.2(5)
C5	C6	C7	C2	-0.1(5)
C7	C2	C3	C4	-1.1(5)
C7	C2	P1	C1	-134.7(3)
C7	C2	P1	C8	104.8(3)
C7	C2	P1	C14	-14.1(3)
C8	C9	C10	C11	0.7(5)
C9	C8	C13	C12	-1.0(5)
C9	C8	P1	C1	-127.6(3)
C9	C8	P1	C2	-7.0(3)
C9	C8	P1	C14	112.5(3)
C9	C10	C11	C12	-1.1(5)
C10	C11	C12	C13	0.4(5)
C11	C12	C13	C8	0.6(5)
C13	C8	C9	C10	0.3(4)
C13	C8	P1	C1	55.4(3)
C13	C8	P1	C2	175.9(2)
C13	C8	P1	C14	-64.5(3)
C14	C15	C16	C17	-1.0(6)
C15	C14	C19	C18	0.0(5)

Atom	Atom	Atom	Atom	Angle/˚
C15	C14	P1	C1	40.7(3)
C15	C14	P1	C2	-80.6(3)
C15	C14	P1	C8	161.3(3)
C15	C16	C17	C18	1.1(6)
C16	C17	C18	C19	-0.6(6)
C17	C18	C19	C14	0.1(5)
C19	C14	C15	C16	0.5(5)
C19	C14	P1	C1	-141.6(3)
C19	C14	P1	C2	97.1(3)
C19	C14	P1	C8	-21.0(3)
P1	C2	C3	C4	174.1(2)
P1	C2	C7	C6	-173.9(2)
P1	C8	C9	C10	-176.7(2)
P1	C8	C13	C12	176.1(2)
P1	C14	C15	C16	178.2(3)
P1	C14	C19	C18	-177.7(3)
C22	C23	C24	C25	-0.3(5)
C23	C22	C27	C26	0.2(6)
C23	C22	P21	C21	131.4(3)
C23	C22	P21	C28	-108.5(3)
C23	C22	P21	C34	11.2(3)
C23	C24	C25	C26	0.3(6)
C24	C25	C26	C27	0.0(7)
C25	C26	C27	C22	-0.2(7)
C27	C22	C23	C24	0.1(5)
C27	C22	P21	C21	-48.1(3)
C27	C22	P21	C28	71.9(3)
C27	C22	P21	C34	-168.4(3)
C28	C29	C30	C31	-0.1(6)
C29	C28	C33	C32	0.9(6)
C29	C28	P21	C21	113.0(3)
C29	C28	P21	C22	-8.5(3)
C29	C28	P21	C34	-126.2(3)
C29	C30	C31	C32	-1.0(6)
C30	C31	C32	C33	2.1(6)
C31	C32	C33	C28	-2.0(6)
C33	C28	C29	C30	0.2(5)
C33	C28	P21	C21	-62.5(4)
C33	C28	P21	C22	176.0(3)
C33	C28	P21	C34	58.3(3)
C34	C35	C36	C37	0.3(6)
C35	C34	C39	C38	-0.3(6)
C35	C34	P21	C21	-38.8(4)
C35	C34	P21	C22	81.8(3)
C35	C34	P21	C28	-158.4(3)
C35	C36	C37	C38	-1.0(7)
C36	C37	C38	C39	1.0(6)
C37	C38	C39	C34	-0.4(6)
C39	C34	C35	C36	0.3(6)
C39	C34	P21	C21	148.8(3)
C39	C34	P21	C22	-90.6(3)
C39	C34	P21	C28	29.2(4)
P21	C22	C23	C24	-179.5(3)
P21	C22	C27	C26	179.7(3)
P21	C28	C29	C30	-175.2(3)
P21	C28	C33	C32	176.4(3)
P21	C34	C35	C36	-172.1(3)
P21	C34	C39	C38	172.0(3)

Table S6: Hydrogen Fractional Atomic Coordinates (×10[4]) and Equivalent Isotropic Displacement Parameters (Å^2×10^3) for **2009src0115**. U_{eq} is defined as 1/3 of the trace of the orthogonalised U_{ij}.

Atom	x	y	z	U_{eq}
H7A	-1112.79	11488.25	2298.23	36
H8	4210.07	10248.46	1594.89	40
H9A	-519.68	6680.75	2207.91	42
H10A	-995.22	9584.16	-229.1	48
H17A	11015.91	7783.89	2567.19	46
H18A	11038.96	5333.93	5175.51	37
H19A	10329.56	2504.16	2663.73	35
H20	5927.23	5261.56	3587.34	58
H1A	1607.21	10335.29	3178.91	47
H1B	564.2	10276.11	3825.33	47
H1C	1422.76	9211.34	3515.84	47
H3	912.79	11807.62	4426.29	33
H4	1127.52	13472.16	4650.41	39
H5	3263.55	13865.04	4561.05	37
H6	5189.11	12629.59	4207.04	34
H7	4995	10951.94	3974.35	31
H9	2618.47	10498.5	5409.75	27
H10	2239.35	9647.88	6443.73	30
H11	1861.75	7939.11	6533.67	30
H12	1788.54	7094.92	5600.74	32
H13	2124.09	7945.34	4560.62	31
H15	3978.18	9751.42	2789.24	39
H16	6035.52	8814.98	2290.61	45
H17	7612.26	7721.59	2929.65	40
H18	7125.68	7511.46	4047.86	39
H19	5067.96	8419.55	4553.05	35
H21A	6268.64	8717.24	252.33	80
H21B	4634.7	9019.69	284.09	80
H21C	5544.1	8948.8	-410.21	80
H23	5380.05	5921.94	-946.22	44
H24	3722.1	5696.06	-1567.88	55
H25	1512.42	6731.37	-1410.19	76
H26	992.51	7989.81	-637.82	80
H27	2639.4	8238.01	-16.22	65
H29	3294.75	6468.29	798.22	42
H30	2871.45	5959.01	1907.84	49
H31	4438.54	5985.48	2629.34	53
H32	6494.95	6460.3	2254.41	53
H33	6909.33	7023.15	1151.83	49
H35	7342.66	7745.04	-995.34	49
H36	9257.81	6748	-1551.97	60
H37	10078.4	4993.24	-1215.88	55
H38	9027.03	4217.89	-292.32	51
H39	7092.72	5205.8	276.42	46
H21	12624.43	2909.15	2655.25	45
H22	12360.03	4399.29	3095.89	36
H23A	15555.99	3850.15	2977.92	40
H31A	16409.22	2114.66	2474.72	40
H32A	14678.06	1739.48	2124.35	45
H33A	18058.15	188.11	1681.92	51
H42A	5708.8	3932.47	5535.41	64
H42B	5778.84	4594.33	4976.19	64
H41A	4541.8	6008.2	5266.78	64
H41B	4969.58	4931.09	5232.7	64
H43A	4175.24	5569.79	3979.9	64
H43B	4223.46	6620.77	3926.08	64
H44A	4825.93	6021.65	4218.28	64
H44B	3863.36	5914.3	3828.7	64

Table S7: Hydrogen Bond information for **2009src0115**.

D	H	A	d(D-H)/Å	d(H-A)/Å	d(D-A)/Å	D-H-A/deg
O7	H7A	O31[1]	0.84	1.88	2.714(3)	174.2
O8	H8	O32[2]	0.84	1.93	2.748(3)	164.7
O9	H9A	O11[3]	0.84	1.99	2.828(3)	172.3
O10	H10A	O6[4]	0.84	1.97	2.803(3)	174.1
O17	H17A	O4[5]	0.84	1.95	2.779(3)	171.8
O18	H18A	O13[6]	0.84	1.93	2.764(3)	174.0
O19	H19A	O7[7]	0.84	1.85	2.673(3)	166.7
O20	H20	O42[8]	0.84	2.39	3.057(9)	136.7
O20	H20	O43	0.84	1.88	2.676(5)	158.2
O20	H20	O44	0.84	1.83	2.622(5)	157.5
O21	H21	O19	0.84	1.85	2.676(3)	166.5
O22	H22	O14	0.84	1.86	2.695(3)	171.4
O23	H23A	O20[5]	0.84	1.88	2.710(3)	168.7
O31	H31A	O23	0.84	1.87	2.699(3)	170.2
O32	H32A	O21	0.84	1.84	2.671(3)	169.4
O33	H33A	O1[9]	0.84	1.86	2.696(3)	173.2

––––

[1]-2+x,1+y,+z; [2]-1+x,1+y,+z; [3]-1+x,+y,+z; [4]-x,2-y,-z; [5]1+x,+y,+z; [6]2-x,1-y,1-z; [7]1+x,-1+y,+z; [8]1-x,1-y,1-z; [9]2+x,-1+y,+z

Table S8: Atomic Occupancies for all atoms that are not fully occupied in **2009src0115**.

Atom	Occupancy
O42	0.217(3)
H42A	0.217(3)
H42B	0.217(3)
O41	0.158(3)
H41A	0.158(3)
H41B	0.158(3)
O43	0.281(3)
H43A	0.281(3)
H43B	0.281(3)
O44	0.344(3)
H44A	0.344(3)
H44B	0.344(3)

Citations for 1

COLLECT (Hooft, R.W.W., 1998)

DENZO (Otwinowski & Minor, 1997)

O.V. Dolomanov and L.J. Bourhis and R.J. Gildea and J.A.K. Howard and H. Puschmann, Olex2: A complete structure solution, refinement and analysis program, *J. Appl. Cryst.*, (2009), **42**, 339-341.

Sheldrick, G.M., A short history of ShelX, *Acta Cryst.*, (2008), **A64**, 339-341.

Sheldrick, G.M., Crystal structure refinement with ShelXL, *Acta Cryst.*, (2015), **C71**, 3-8.

[$MePPh_3$][$B_5O_6(OH)_4$]·$B(OH)_3$·$0.5H_2O$ Compound 2

Submitted by: **Mike A Beckett**
Bangor University
Solved by: **Peter N Horton**
Sample ID: **MAB/JT/33**

R_1=5.63%

Crystal Data and Experimental

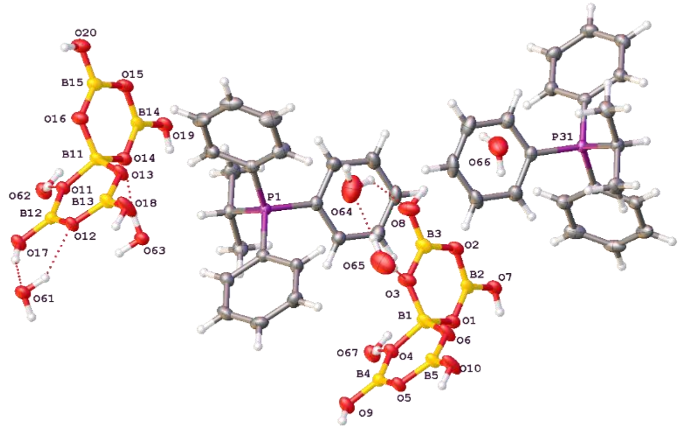

Experimental. Single colourless block-shaped crystals of **2010src0454b** (**2**) were used as supplied. A suitable crystal with dimensions 0.260 × 0.180 × 0.120 mm^3 was selected and mounted on a Damien diffractometer. The crystal was kept at a steady T = 120(2) K during data collection. The structure was solved with the ShelXS-97 (Sheldrick, 2008) solution program using direct methods and by using Olex2 1.5-alpha (Dolomanov et al., 2009) as the graphical interface. The model was refined with ShelXL 2018/3 (Sheldrick, 2015) using full matrix least squares minimisation on $\boldsymbol{F}^2$.

Crystal Data. $C_{21}H_{33}B_5O_{13.5}P$, M_r = 586.49, triclinic, P-1 (No. 2), a = 9.1810(3) Å, b = 13.4812(5) Å, c = 23.8357(8) Å, α = 75.3130(10)°, β = 83.095(2)°, γ = 86.919(2)°, V = 2832.24(17) Å^3, T = 120(2) K, Z = 4, Z' = 2, μ(MoK$_\alpha$) = 0.162 mm^{-1}, 50870 reflections measured, 12947 unique (R_{int} = 0.0578) which were used in all calculations. The final wR_2 was 0.1571 (all data) and R_1 was 0.0563 (I≥2 σ(I)).

Compound 2 **2010src0454b**

Formula	$C_{21}H_{33}B_5O_{13.5}P$
$D_{calc.}$/ g cm^{-3}	1.375
μ/mm^{-1}	0.162
Formula Weight	586.49
Colour	colourless
Shape	block-shaped
Size/mm^3	0.260×0.180×0.120
T/K	120(2)
Crystal System	triclinic
Space Group	*P*-1
a/Å	9.1810(3)
b/Å	13.4812(5)
c/Å	23.8357(8)
α/°	75.3130(10)
β/°	83.095(2)
γ/°	86.919(2)
V/Å^3	2832.24(17)
Z	4
Z'	2
Wavelength/Å	0.71073
Radiation type	MoK$_\alpha$
Θ_{min}/°	2.941
Θ_{max}/°	27.605
Measured Refl's.	50870
Indep't Refl's	12947
Refl's I≥2 σ(I)	9308
R_{int}	0.0578
Parameters	763
Restraints	1
Largest Peak	0.940
Deepest Hole	-0.540
GooF	1.038
wR_2 (all data)	0.1571
wR_2	0.1416
R_1 (all data)	0.0831
R_1	0.0563

Structure Quality Indicators

**Reflections:**	d min (MoKα) 2Θ=55.2° 0.77	I/σ(I) 18.4	Rint m=3.92 5.78%	Full 50.5° 99% to 55.2° 99.7
**Refinement:**	Shift 0.001	Max Peak 0.9	Min Peak -0.5	GooF 1.038

A colourless block-shaped-shaped crystal with dimensions 0.260 × 0.180 × 0.120 mm^3 was mounted. Data were collected using a Damien diffractometer equipped with an Oxford Cryosystems low-temperature device operating at T = 120(2) K.

Data were measured using ϕ & ω scans with MoK$_\alpha$ radiation. The diffraction pattern was indexed and the total number of runs and images was based on the strategy calculation from the program COLLECT (Hooft, R.W.W., 1998). The maximum resolution that was achieved was Θ = 27.605° (0.77 Å).

The unit cell was refined using DENZO (Otwinowski & Minor, 1997) & COLLECT (Hooft, R.W.W., 1998) on 12555 reflections, 25% of the observed reflections.

Data reduction, scaling and absorption corrections were performed using DENZO (Otwinowski & Minor, 1997) & COLLECT (Hooft, R.W.W., 1998). The final completeness is 99.70 % out to 27.605° in Θ. SADABS

2007/2 (Sheldrick, G.M., 2007). The absorption coefficient μ of this material is 0.162 mm^{-1} at this wavelength (λ = 0.71073Å) and the minimum and maximum transmissions are 0.959 and 0.981.

The structure was solved and the space group *P*-1 (# 2) determined by the ShelXS-97 (Sheldrick, 2008) structure solution program using using direct methods and refined by full matrix least squares minimisation on $\boldsymbol{F^2}$ using version 2018/3 of ShelXL 2018/3 (Sheldrick, 2015). All non-hydrogen atoms were refined anisotropically. Hydrogen atom positions were calculated geometrically and refined using the riding model. Hydrogen atom positions were calculated geometrically and refined using the riding model.

_refine_special_details: There is a pseudo body-centring, but multiple reflections are observed which should be absent if it truly existed

_exptl_absorpt_process_details: SADABS 2007/2 (Sheldrick, G.M., 2007)

The value of Z' is 2. This means that there are two independent molecules in the asymmetric unit. The moiety formula is B5 H4 O10, C21 H22 P, 3.5(H2 O).

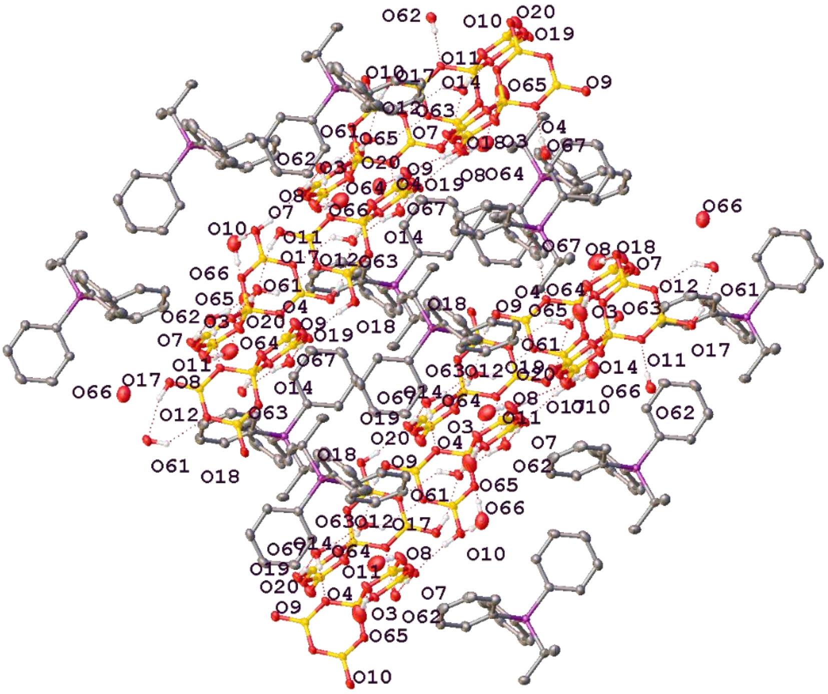

Figure S5 The following hydrogen bonding interactions with a maximum D-D distance of 2.9 Å and a minimum angle of 120 ° are present in **2010src0454b**: O7–O62_*4: 2.695 Å, O8–O20*_5: 2.685 Å, O9–O63_*6: 2.874 Å, O10–O7*_1: 2.734 Å, O17–O61: 2.871 Å, O18–O19_*1: 2.703 Å, O20–O66*_5: 2.703 Å, O61–O12: 2.878 Å, O61–O1_*6: 2.711 Å, O62–O11: 2.788 Å, O62–O61*_2: 2.84 Å, O63–O14: 2.698 Å, O63–O5_*6: 2.887 Å, O64–O18*_3: 2.936 Å, O64–O8: 2.829 Å, O65–O3: 2.797 Å, O65–O64: 2.714 Å, O66–O10_*2: 3.069 Å, O66–O65*_2: 2.679 Å, O67–O4: 2.777 Å, O67–O63_*4: 2.856 Å, O19–O67*_4: 2.699 Å.

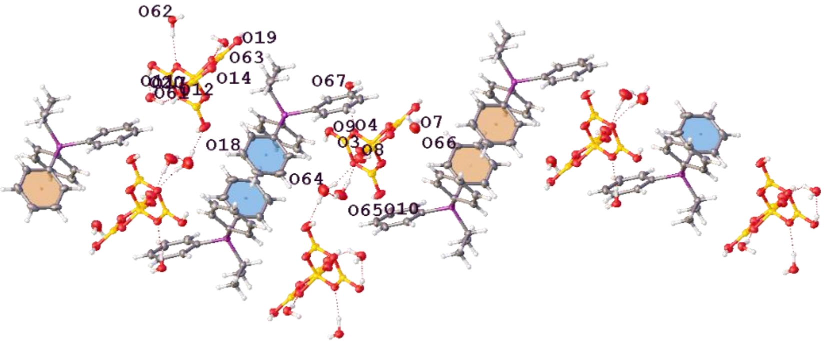

Figure S6 The π-π interactions of 2010src0454b. **Plane 1 *to* #1@2_575 (-X,2-Y,-Z):** The angle between these two planes is 0.000 °, the Centroid-Centroid distance is 3.748 Å and the Shift Distance is 0.917 Å **Plane 5 *to* #5@2_666 (1-X,1-Y,1-Z):** The angle between these two planes is 0.000 °, the Centroid-Centroid distance is 3.712 Å and the Shift Distance is 1.155 Å

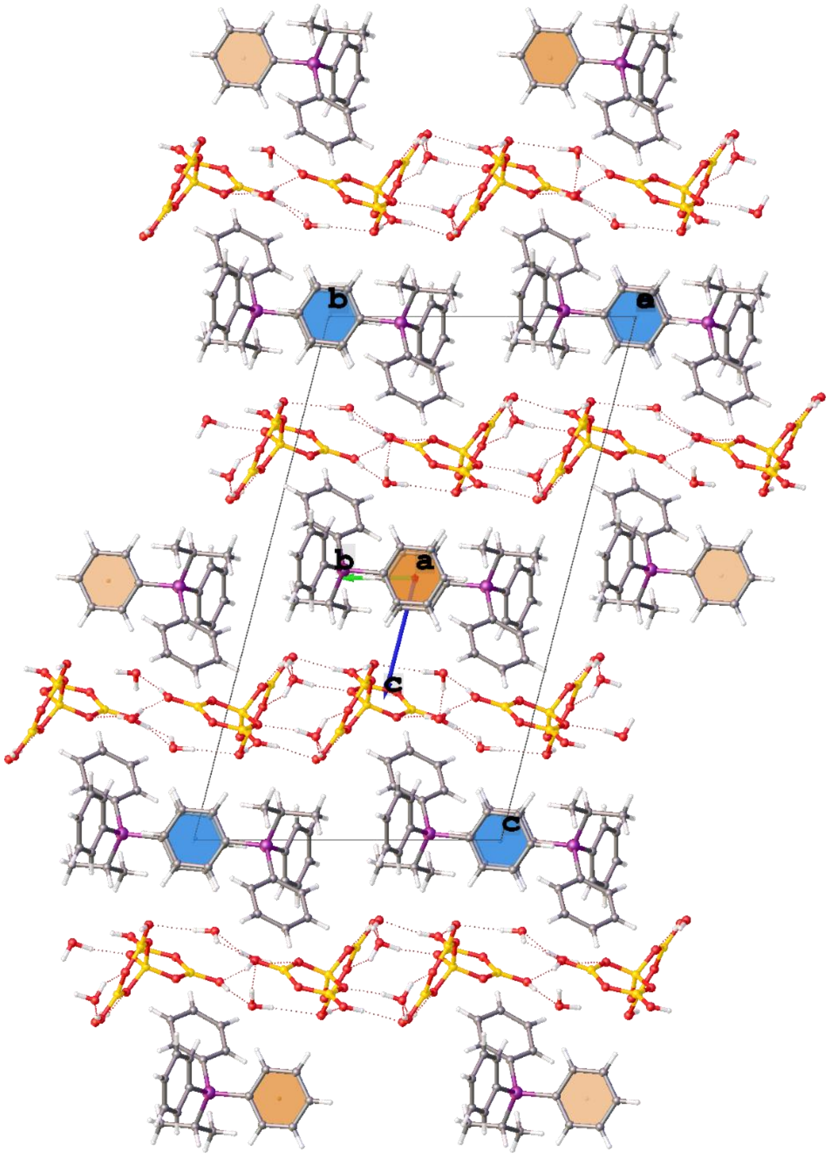

Figure S7 Packing diagram of 2010src0454b viewed along the a axis

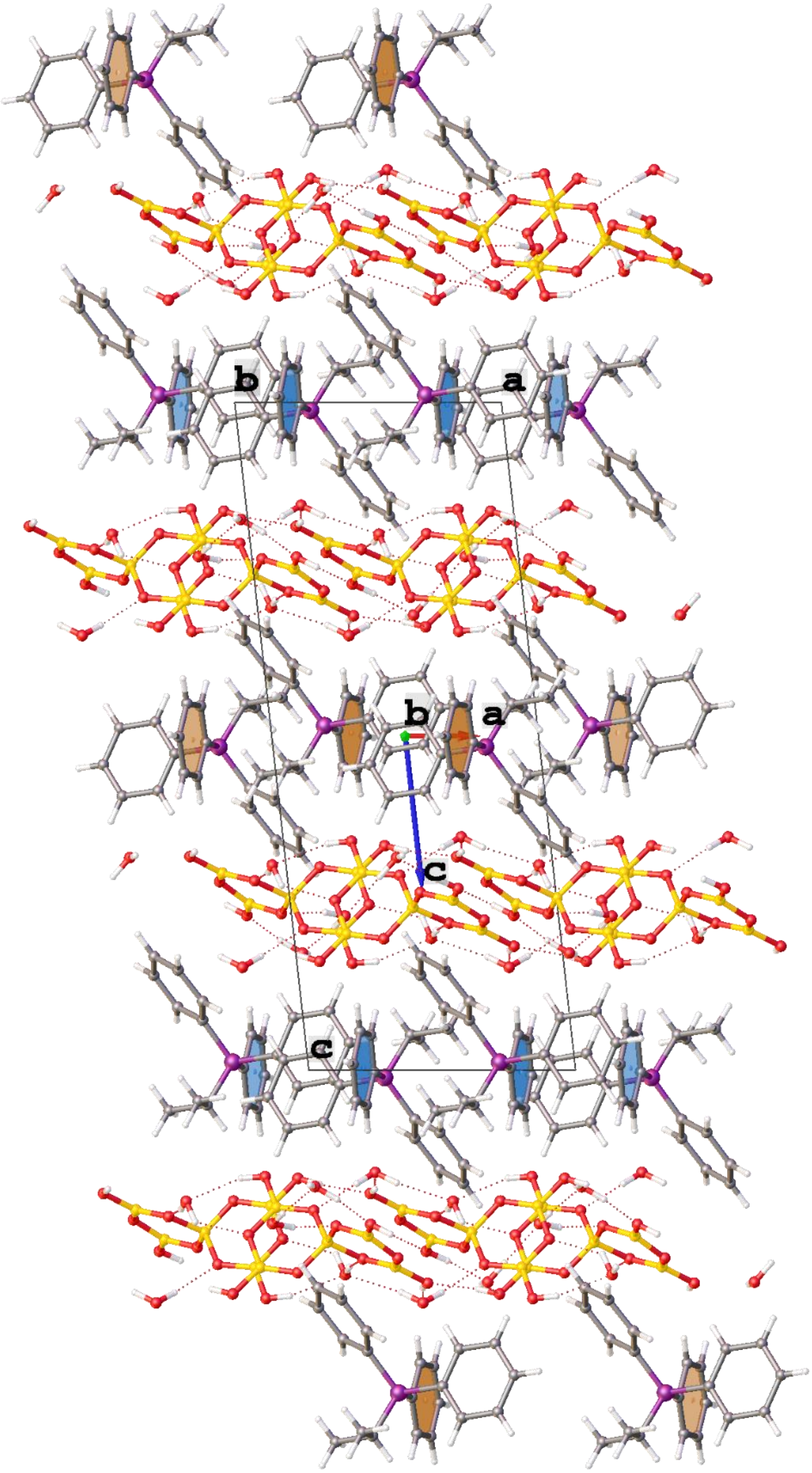

Figure S8 Packing diagram of 2010src0454b viewed along the b axis

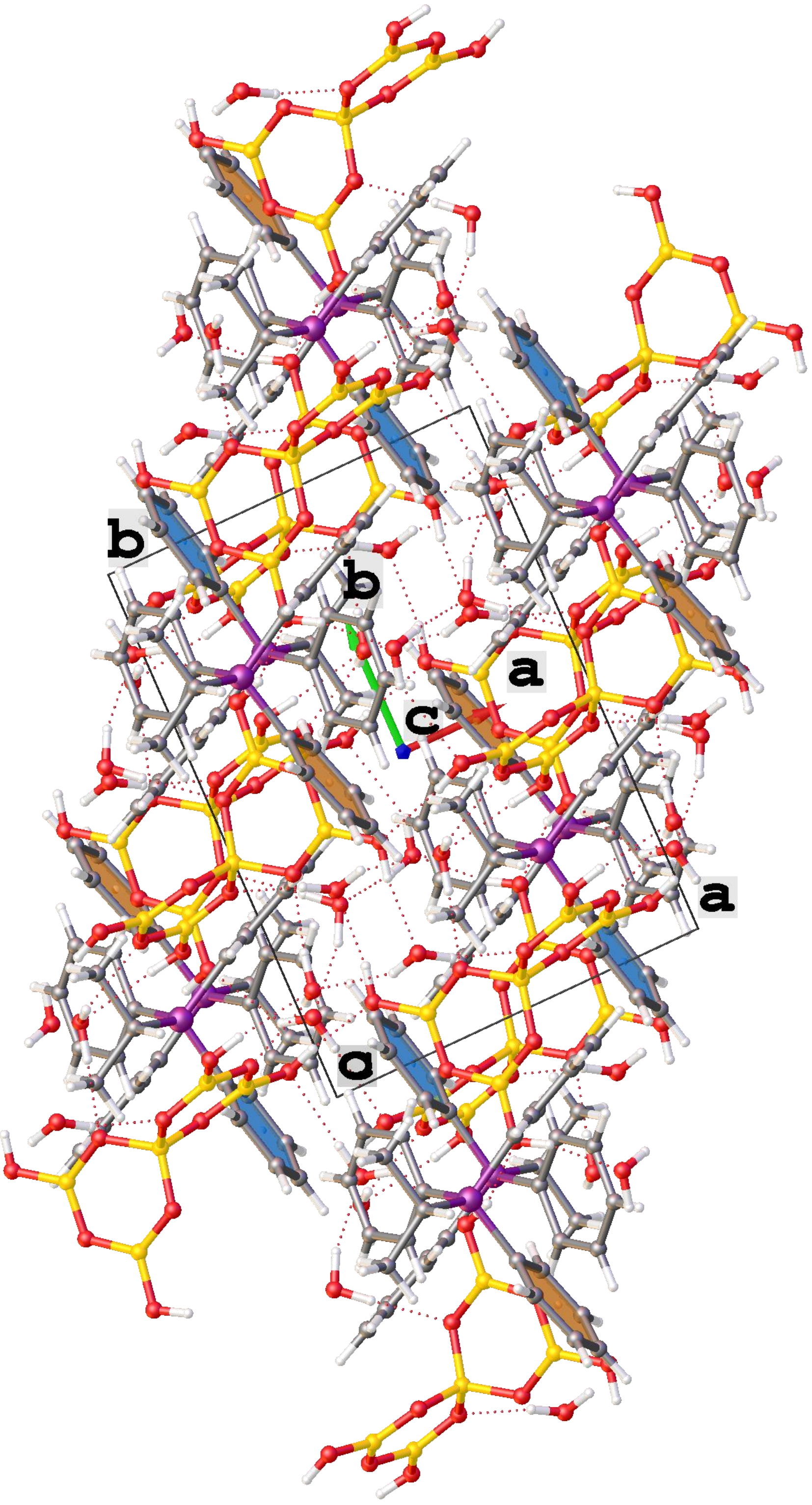

Figure S9 Packing diagram of 2010src0454b viewed along the c axis

Table S9: Fractional Atomic Coordinates (×10[4]) and Equivalent Isotropic Displacement Parameters (Å[2]×10[3]) for **2010src0454b**. U_{eq} is defined as 1/3 of the trace of the orthogonalised U_{ij}.

Atom	x	y	z	U_{eq}
B1	-21(2)	4492(2)	2582.9(11)	23.8(5)
B2	2426(2)	4321(2)	2949.3(10)	21.7(5)
B3	1534(3)	5989(2)	2490.3(12)	25.9(5)
B4	-1784(2)	3819.3(18)	2059.7(10)	19.6(5)

Atom	x	y	z	U_{eq}
B5	-2637(2)	4251(2)	2961.8(11)	23.9(5)
O1	1240.3(15)	3881.2(12)	2833.4(7)	23.9(3)
O2	2557.8(15)	5367.7(12)	2820.4(7)	25.8(3)
O3	373.4(15)	5565.8(12)	2342.6(7)	27.8(4)
O4	-398.4(14)	4054.7(11)	2109.3(6)	22.3(3)
O5	-2940.2(14)	3939.2(11)	2474.6(6)	21.7(3)
O6	-1259.7(15)	4434.7(13)	3039.1(7)	29.4(4)
O7	3565.5(15)	3738.7(12)	3196.1(7)	25.0(3)
O8	1675.3(17)	7017.6(12)	2314.1(8)	36.5(4)
O9	-2078.8(15)	3462.9(13)	1599.6(7)	27.1(4)
O10	-3780.5(16)	4385.0(15)	3359.7(7)	37.8(4)
B11	4574(2)	9493.9(19)	-2432.6(11)	21.8(5)
B12	2838(2)	8806.6(18)	-2960.6(10)	18.8(5)
B13	1984(2)	9160(2)	-2037.7(11)	23.9(5)
B14	7063(2)	9341(2)	-2098.9(10)	21.8(5)
B15	6112(3)	10990(2)	-2543.3(11)	24.2(5)
O11	4214.9(14)	9069.7(11)	-2916.5(6)	20.8(3)
O12	1687.9(14)	8877.8(11)	-2535.6(6)	21.9(3)
O13	3361.2(15)	9369.1(12)	-1965.5(6)	26.0(3)
O14	5865.8(15)	8889.4(11)	-2199.0(7)	23.6(3)
O15	7195.4(15)	10386.4(12)	-2245.8(7)	27.9(4)
O16	4913.3(15)	10573.5(11)	-2657.7(7)	26.5(3)
O17	2550.5(15)	8466.4(13)	-3427.9(7)	26.4(3)
O18	857.6(16)	9241.4(16)	-1625.9(8)	39.5(5)
O19	8208.0(15)	8757.3(12)	-1853.4(7)	27.1(4)
O20	6348.2(17)	12022.4(12)	-2719.1(8)	33.1(4)
C1	2430(2)	8949.3(16)	33.4(9)	20.5(4)
C2	2460(2)	9610.3(17)	-522.9(10)	27.9(5)
C3	2076(3)	10637.2(18)	-576.4(12)	35.5(6)
C4	1686(2)	10997.6(19)	-84.0(13)	35.5(6)
C5	1643(3)	10344(2)	460.8(13)	39.6(6)
C6	2004(3)	9312.9(18)	526.7(11)	32.3(5)
C7	1105(2)	6946.0(15)	226.1(9)	19.8(4)
C8	481(2)	6762.0(17)	-245.9(10)	23.7(5)
C9	-896(2)	6327.8(18)	-149.7(11)	29.2(5)
C10	-1636(2)	6079.3(17)	407.6(11)	29.6(5)
C11	-1019(2)	6258.8(18)	875.1(11)	29.9(5)
C12	352(2)	6695.1(17)	786.8(10)	25.3(5)
C13	3772(2)	7099.3(16)	745.2(9)	20.9(4)
C14	4656(2)	7726.6(18)	939.9(10)	27.6(5)
C15	5416(2)	7315(2)	1418.6(11)	33.3(6)
C16	5322(2)	6280(2)	1697.5(10)	31.6(5)
C17	4480(2)	5651.7(19)	1496.0(10)	29.1(5)
C18	3692(2)	6052.4(17)	1025.8(9)	24.2(5)
C19	3962(2)	7398.8(16)	-525.7(9)	22.0(4)
C20	4270(2)	6249.4(17)	-466.8(10)	27.7(5)
C21	5404(2)	7973.6(19)	-610.5(11)	31.1(5)
P1	2813.8(5)	7602.6(4)	115.7(2)	17.53(13)
C31	1041(2)	7861.8(17)	4213.1(9)	21.6(4)
C32	1085(2)	8895.6(18)	3916.6(10)	27.3(5)
C33	281(3)	9259(2)	3445.4(11)	35.0(6)
C34	-556(3)	8592(2)	3267.2(10)	37.3(6)
C35	-616(3)	7570(2)	3562.5(11)	34.4(6)
C36	170(2)	7202.9(19)	4037.7(10)	28.4(5)
C37	2498(2)	6061.1(16)	4905.0(9)	20.4(4)
C38	2544(2)	5384.6(17)	5453.6(10)	26.7(5)
C39	3044(3)	4385.5(18)	5492.2(12)	33.5(6)
C40	3482(2)	4057.1(18)	4992.2(12)	33.9(6)
C41	3438(2)	4723.8(19)	4448.6(12)	34.0(6)
C42	2962(2)	5731.0(17)	4399.0(10)	26.8(5)
C43	3701(2)	8076.6(15)	4739.0(9)	19.6(4)
C44	4297(2)	8271.8(16)	5215.3(10)	23.3(5)

Atom	x	y	z	U_{eq}
C45	5664(2)	8715.3(17)	5126.3(10)	27.8(5)
C46	6432(2)	8962.6(18)	4574.6(11)	30.2(5)
C47	5846(2)	8768.2(18)	4103.4(10)	28.4(5)
C48	4482(2)	8321.9(16)	4184.5(9)	23.5(5)
C49	865(2)	7563.2(16)	5486.8(9)	21.8(4)
C50	-542(2)	6950.1(19)	5577.5(10)	31.0(5)
C51	494(2)	8705.9(18)	5428.7(10)	28.5(5)
P31	2023.8(5)	7391.3(4)	4840.3(2)	17.59(13)
O61	-517.3(16)	8117.1(12)	-3070.2(7)	27.2(3)
O62	6688.2(16)	8316.1(13)	-3490.9(7)	28.3(4)
O63	5140.9(16)	6902.2(12)	-1970.7(7)	24.4(3)
O64	-252(3)	8557.0(18)	1750.5(10)	62.1(6)
O65	-2000(2)	6962.5(17)	2305.4(11)	56.4(6)
O66	5825(2)	6715.0(15)	3181.5(9)	47.5(5)
O67	2061.9(16)	3299.2(13)	1537.7(7)	27.9(4)

Table S10: Anisotropic Displacement Parameters (×10[4]) for **2010src0454b.** The anisotropic displacement factor exponent takes the form: $-2\pi^2[h^2a^{*2} \times U_{11} + ... + 2hka^* \times b^* \times U_{12}]$

Atom	U_{11}	U_{22}	U_{33}	U_{23}	U_{13}	U_{12}
B1	11.9(10)	33.9(14)	28.1(14)	-12.1(11)	-1.6(9)	-2.7(9)
B2	14.1(10)	34.3(14)	17.7(12)	-9.1(10)	2.2(9)	-5.6(9)
B3	15.3(11)	32.8(15)	32.6(15)	-13.4(12)	-2.8(10)	0.1(10)
B4	15.3(10)	23.6(12)	19.9(12)	-4.7(10)	-2.7(9)	-0.9(9)
B5	13.6(10)	36.4(15)	25.1(13)	-13.1(11)	-2.0(9)	-4.8(9)
O1	13.6(7)	30.3(9)	28.8(9)	-7.5(7)	-4.2(6)	-4.6(6)
O2	15.0(7)	32.0(9)	32.8(9)	-10.4(7)	-4.2(6)	-5.2(6)
O3	15.7(7)	28.4(9)	42.4(10)	-12.6(7)	-7.7(7)	-0.7(6)
O4	12.9(6)	31.4(9)	24.9(8)	-11.2(7)	-1.5(6)	-2.2(6)
O5	12.3(6)	33.0(9)	22.8(8)	-11.6(7)	-2.3(6)	-2.9(6)
O6	13.4(7)	53.1(11)	28.0(9)	-21.2(8)	-1.1(6)	-5.7(7)
O7	14.6(7)	33.1(9)	28.1(9)	-7.7(7)	-4.0(6)	-5.2(6)
O8	22.7(8)	29.4(9)	60.8(12)	-13.8(8)	-11.9(8)	-2.1(7)
O9	16.7(7)	42.0(10)	26.5(9)	-14.9(7)	-3.5(6)	-2.4(7)
O10	15.0(7)	71.9(13)	36.6(10)	-32.2(9)	2.0(7)	-11.6(8)
B11	14.0(10)	28.7(13)	25.2(13)	-10.3(10)	-2.6(9)	-3.0(9)
B12	16.1(10)	20.9(12)	18.9(12)	-4.1(9)	-1.2(8)	-1.5(9)
B13	14.9(10)	33.8(14)	25.8(13)	-11.9(11)	-3.6(9)	-1.2(9)
B14	14.1(10)	33.1(14)	19.8(12)	-9.6(10)	0.6(9)	-4.8(9)
B15	19.3(11)	27.6(14)	27.7(14)	-11.5(11)	1.0(9)	-4.2(9)
O11	12.7(6)	27.1(8)	23.6(8)	-7.7(6)	-2.0(5)	-3.7(6)
O12	12.9(6)	33.4(9)	21.9(8)	-10.4(7)	-2.9(6)	-3.8(6)
O13	14.3(7)	42.7(10)	25.2(8)	-15.1(7)	-2.3(6)	-5.5(6)
O14	14.4(7)	27.1(8)	30.1(9)	-6.4(7)	-6.1(6)	-3.3(6)
O15	17.6(7)	31.7(9)	36.7(10)	-10.6(7)	-4.7(6)	-7.9(6)
O16	19.8(7)	24.9(8)	36.9(9)	-9.0(7)	-7.4(6)	-2.5(6)
O17	15.9(7)	41.3(10)	26.1(8)	-14.9(7)	-2.7(6)	-3.6(6)
O18	15.5(7)	78.2(14)	35.1(10)	-33.1(9)	2.1(7)	-12.3(8)
O19	14.4(7)	36.3(9)	32.5(9)	-9.6(7)	-5.9(6)	-4.7(6)
O20	27.3(8)	28.4(9)	46.6(11)	-12.8(8)	-6.8(8)	-5.1(7)
C1	12.3(9)	22.6(11)	26.7(12)	-6.2(9)	-2.8(8)	0.4(8)
C2	28.1(11)	24.8(12)	32.7(13)	-6.9(10)	-12.5(10)	0.5(9)
C3	33.9(13)	22.3(12)	49.4(16)	0.4(11)	-20.6(12)	-2.0(10)
C4	17.7(10)	23.3(12)	67.1(19)	-13.7(12)	-6.3(11)	2.0(9)
C5	34.8(13)	31.8(14)	52.9(18)	-19.9(13)	12.5(12)	0.0(11)
C6	32.9(12)	29.0(13)	33.5(14)	-11.7(11)	10.9(10)	-0.4(10)
C7	17.7(9)	19.2(11)	22.9(11)	-5.4(8)	-3.6(8)	0.4(8)
C8	21.8(10)	27.0(12)	24.0(12)	-9.5(9)	-3.8(8)	1.1(8)
C9	25.7(11)	30.7(13)	37.1(14)	-15.5(11)	-12.1(10)	0.0(9)
C10	21.2(11)	23.6(12)	43.2(15)	-5.5(10)	-5.5(10)	-4.7(9)

Atom	U_{11}	U_{22}	U_{33}	U_{23}	U_{13}	U_{12}
C11	22.1(11)	34.2(13)	29.2(13)	-0.4(10)	0.3(9)	-5.6(9)
C12	21.4(10)	32.2(13)	21.1(11)	-3.8(9)	-2.6(8)	-3.8(9)
C13	17.0(9)	26.2(12)	19.0(11)	-5.8(9)	-0.8(8)	0.8(8)
C14	23.2(11)	31.7(13)	26.9(12)	-3.8(10)	-4.4(9)	-6.3(9)
C15	23.9(11)	49.0(16)	27.7(13)	-7.1(11)	-7.5(9)	-8.4(10)
C16	22.6(11)	46.5(15)	22.6(12)	-1.8(11)	-6.4(9)	3.6(10)
C17	26.5(11)	31.2(13)	26.6(12)	-2.3(10)	-3.8(9)	4.1(9)
C18	23.4(10)	23.9(12)	24.4(12)	-4.0(9)	-3.1(9)	-0.6(8)
C19	20.1(10)	25.4(11)	20.3(11)	-6.0(9)	-1.6(8)	1.1(8)
C20	30.2(11)	26.2(12)	28.6(13)	-11.0(10)	-3.8(9)	5.7(9)
C21	21.3(11)	40.3(14)	30.5(13)	-10.7(11)	7.6(9)	-4.6(10)
P1	15.3(2)	19.1(3)	18.0(3)	-4.5(2)	-1.13(19)	-0.17(19)
C31	16.8(9)	27.5(12)	19.3(11)	-4.3(9)	-1.0(8)	1.7(8)
C32	23.4(11)	29.5(13)	26.8(12)	-3.8(10)	-2.1(9)	2.9(9)
C33	28.7(12)	38.4(15)	28.8(13)	5.1(11)	-1.6(10)	10.0(10)
C34	24.6(11)	62.2(18)	20.7(12)	-2.2(12)	-6.9(9)	9.6(11)
C35	25.4(11)	53.0(17)	26.5(13)	-10.1(12)	-8.1(10)	-4.1(11)
C36	22.5(11)	35.2(13)	26.5(12)	-4.3(10)	-4.7(9)	-3.4(9)
C37	15.4(9)	22.1(11)	24.5(11)	-6.0(9)	-4.0(8)	-0.7(8)
C38	27.6(11)	24.3(12)	30.6(13)	-7.4(10)	-11.0(9)	-1.0(9)
C39	30.5(12)	23.7(12)	46.1(15)	-0.7(11)	-19.5(11)	-2.3(9)
C40	19.8(11)	22.7(12)	63.3(18)	-16.2(12)	-9.6(11)	0.9(9)
C41	21.7(11)	34.3(14)	49.9(16)	-22.1(12)	6.0(10)	-1.4(10)
C42	24.1(11)	28.0(12)	29.1(13)	-10.7(10)	2.7(9)	-2.0(9)
C43	16.5(9)	17.3(10)	24.4(11)	-4.4(8)	-2.5(8)	0.9(8)
C44	22.0(10)	25.4(12)	24.2(12)	-8.8(9)	-3.4(8)	-0.4(8)
C45	24.2(11)	30.7(13)	32.1(13)	-12.1(10)	-8.1(9)	-0.1(9)
C46	20.3(10)	27.5(13)	42.8(15)	-6.7(11)	-6.1(10)	-4.3(9)
C47	20.4(10)	30.9(13)	29.6(13)	-1.2(10)	1.5(9)	-3.5(9)
C48	20.3(10)	27.5(12)	21.8(11)	-5.2(9)	-1.4(8)	-1.3(8)
C49	17.9(9)	26.9(12)	20.9(11)	-8.0(9)	0.4(8)	1.0(8)
C50	22.6(11)	40.7(14)	29.0(13)	-10.1(11)	5.4(9)	-5.6(10)
C51	24.4(11)	33.4(13)	30.2(13)	-14.1(10)	-2.5(9)	5.7(9)
P31	15.1(2)	20.3(3)	17.4(3)	-4.8(2)	-1.97(19)	0.30(19)
O61	22.3(7)	31.6(9)	29.9(9)	-9.3(7)	-5.7(6)	-6.8(7)
O62	18.6(7)	38.0(10)	30.8(9)	-13.3(7)	-3.9(6)	1.9(7)
O63	21.0(7)	25.9(8)	27.5(9)	-6.0(7)	-7.3(6)	-3.8(6)
O64	60.8(14)	69.3(16)	48.3(14)	-3.3(12)	-7.9(11)	17.1(12)
O65	37.5(11)	60.0(14)	80.6(17)	-28.9(13)	-24.4(11)	16.1(9)
O66	57.2(13)	39.2(11)	49.1(13)	-14.2(9)	-15.5(10)	8.3(9)
O67	17.1(7)	39.4(10)	30.5(9)	-14.9(7)	-4.2(6)	3.1(7)

Table S11: Bond Lengths in Å for **2010src0454b**.

Atom	Atom	Length/Å
B1	O1	1.480(3)
B1	O3	1.463(3)
B1	O4	1.482(3)
B1	O6	1.467(3)
B2	O1	1.356(3)
B2	O2	1.374(3)
B2	O7	1.372(3)
B3	O2	1.401(3)
B3	O3	1.357(3)
B3	O8	1.352(3)
B4	O4	1.354(3)
B4	O5	1.393(3)
B4	O9	1.364(3)
B5	O5	1.393(3)
B5	O6	1.344(3)

Atom	Atom	Length/Å
B5	O10	1.364(3)
B11	O11	1.487(3)
B11	O13	1.461(3)
B11	O14	1.485(3)
B11	O16	1.455(3)
B12	O11	1.354(2)
B12	O12	1.390(3)
B12	O17	1.365(3)
B13	O12	1.395(3)
B13	O13	1.350(3)
B13	O18	1.358(3)
B14	O14	1.358(3)
B14	O15	1.372(3)
B14	O19	1.376(3)
B15	O15	1.395(3)

Atom	Atom	Length/Å
B15	O16	1.348(3)
B15	O20	1.369(3)
C1	C2	1.396(3)
C1	C6	1.392(3)
C1	P1	1.796(2)
C2	C3	1.388(3)
C3	C4	1.383(4)
C4	C5	1.369(4)
C5	C6	1.386(3)
C7	C8	1.402(3)
C7	C12	1.397(3)
C7	P1	1.802(2)
C8	C9	1.391(3)
C9	C10	1.386(3)
C10	C11	1.387(3)
C11	C12	1.389(3)
C13	C14	1.398(3)
C13	C18	1.402(3)
C13	P1	1.795(2)
C14	C15	1.387(3)
C15	C16	1.387(4)
C16	C17	1.383(3)
C17	C18	1.385(3)
C19	C20	1.534(3)
C19	C21	1.537(3)
C19	P1	1.821(2)
C31	C32	1.394(3)
C31	C36	1.396(3)
C31	P31	1.798(2)
C32	C33	1.388(3)
C33	C34	1.388(4)
C34	C35	1.380(4)
C35	C36	1.387(3)
C37	C38	1.396(3)
C37	C42	1.401(3)
C37	P31	1.795(2)
C38	C39	1.384(3)
C39	C40	1.383(4)
C40	C41	1.381(4)
C41	C42	1.384(3)
C43	C44	1.405(3)
C43	C48	1.395(3)
C43	P31	1.798(2)
C44	C45	1.390(3)
C45	C46	1.384(3)
C46	C47	1.389(3)
C47	C48	1.390(3)
C49	C50	1.535(3)
C49	C51	1.535(3)
C49	P31	1.822(2)

Table S12: Bond Angles in ° for **2010src0454b**.

Atom	Atom	Atom	Angle/°
O1	B1	O4	107.16(17)
O3	B1	O1	110.41(16)
O3	B1	O4	109.53(19)
O3	B1	O6	109.04(18)
O6	B1	O1	109.58(19)
O6	B1	O4	111.10(16)
O1	B2	O2	122.0(2)
O1	B2	O7	121.4(2)
O7	B2	O2	116.67(18)
O3	B3	O2	120.4(2)
O8	B3	O2	120.97(19)
O8	B3	O3	118.7(2)
O4	B4	O5	120.91(19)
O4	B4	O9	120.53(19)
O9	B4	O5	118.55(18)
O6	B5	O5	121.48(19)
O6	B5	O10	120.15(19)
O10	B5	O5	118.36(18)
B2	O1	B1	122.38(18)
B2	O2	B3	118.55(17)
B3	O3	B1	123.39(18)
B4	O4	B1	123.04(16)
B4	O5	B5	118.87(16)
B5	O6	B1	122.94(17)
O13	B11	O11	111.19(16)
O13	B11	O14	108.68(18)
O14	B11	O11	106.24(17)
O16	B11	O11	109.48(18)
O16	B11	O13	110.15(18)
O16	B11	O14	111.03(16)
O11	B12	O12	121.15(19)
O11	B12	O17	120.38(19)
O17	B12	O12	118.46(18)
O13	B13	O12	121.1(2)
O13	B13	O18	119.9(2)
O18	B13	O12	118.99(18)
O14	B14	O15	121.9(2)
O14	B14	O19	120.7(2)
O15	B14	O19	117.38(18)
O16	B15	O15	121.6(2)
O16	B15	O20	122.2(2)
O20	B15	O15	116.21(19)
B12	O11	B11	122.58(16)
B12	O12	B13	119.02(16)
B13	O13	B11	122.97(17)
B14	O14	B11	122.21(18)
B14	O15	B15	118.40(17)
B15	O16	B11	123.18(18)
C2	C1	P1	120.05(16)
C6	C1	C2	120.4(2)
C6	C1	P1	119.37(17)
C3	C2	C1	119.0(2)
C4	C3	C2	120.2(2)
C5	C4	C3	120.6(2)
C4	C5	C6	120.4(2)
C5	C6	C1	119.3(2)
C8	C7	P1	120.97(16)
C12	C7	C8	120.37(19)
C12	C7	P1	118.47(15)
C9	C8	C7	119.3(2)
C10	C9	C8	120.1(2)
C9	C10	C11	120.7(2)

Atom	Atom	Atom	Angle/°
C10	C11	C12	120.0(2)
C11	C12	C7	119.6(2)
C14	C13	C18	119.6(2)
C14	C13	P1	120.80(17)
C18	C13	P1	119.48(16)
C15	C14	C13	119.8(2)
C16	C15	C14	120.3(2)
C17	C16	C15	120.1(2)
C16	C17	C18	120.5(2)
C17	C18	C13	119.7(2)
C20	C19	C21	110.60(17)
C20	C19	P1	110.42(15)
C21	C19	P1	110.08(15)
C1	P1	C7	108.96(9)
C1	P1	C19	109.87(10)
C7	P1	C19	111.31(10)
C13	P1	C1	109.89(10)
C13	P1	C7	108.13(10)
C13	P1	C19	108.65(9)
C32	C31	C36	119.5(2)
C32	C31	P31	120.34(16)
C36	C31	P31	120.07(17)
C33	C32	C31	119.9(2)
C32	C33	C34	120.0(2)
C35	C34	C33	120.3(2)
C34	C35	C36	120.0(2)

Atom	Atom	Atom	Angle/°
C35	C36	C31	120.2(2)
C38	C37	C42	120.2(2)
C38	C37	P31	120.44(16)
C42	C37	P31	119.08(17)
C39	C38	C37	119.4(2)
C40	C39	C38	120.3(2)
C41	C40	C39	120.5(2)
C40	C41	C42	120.3(2)
C41	C42	C37	119.4(2)
C44	C43	P31	121.06(16)
C48	C43	C44	120.14(19)
C48	C43	P31	118.49(15)
C45	C44	C43	119.2(2)
C46	C45	C44	120.4(2)
C45	C46	C47	120.4(2)
C46	C47	C48	120.0(2)
C47	C48	C43	119.8(2)
C50	C49	C51	110.61(17)
C50	C49	P31	110.30(15)
C51	C49	P31	110.12(15)
C31	P31	C43	109.40(10)
C31	P31	C49	108.88(9)
C37	P31	C31	108.97(10)
C37	P31	C43	107.84(9)
C37	P31	C49	110.84(10)
C43	P31	C49	110.87(10)

Table S13: Torsion Angles in ° for **2010src0454b**.

Atom	Atom	Atom	Atom	Angle/°
O1	B1	O3	B3	-19.0(3)
O1	B1	O4	B4	131.12(19)
O1	B1	O6	B5	-133.5(2)
O1	B2	O2	B3	-8.7(3)
O2	B2	O1	B1	-2.5(3)
O2	B3	O3	B1	9.7(3)
O3	B1	O1	B2	15.3(3)
O3	B1	O4	B4	-109.1(2)
O3	B1	O6	B5	105.5(2)
O3	B3	O2	B2	5.2(3)
O4	B1	O1	B2	134.55(19)
O4	B1	O3	B3	-136.7(2)
O4	B1	O6	B5	-15.3(3)
O4	B4	O5	B5	-3.6(3)
O5	B4	O4	B1	-2.8(3)
O5	B5	O6	B1	10.5(4)
O6	B1	O1	B2	-104.8(2)
O6	B1	O3	B3	101.5(2)
O6	B1	O4	B4	11.4(3)
O6	B5	O5	B4	-0.2(3)
O7	B2	O1	B1	178.25(19)
O7	B2	O2	B3	170.57(19)
O8	B3	O2	B2	-175.0(2)
O8	B3	O3	B1	-170.1(2)
O9	B4	O4	B1	177.0(2)
O9	B4	O5	B5	176.6(2)
O10	B5	O5	B4	178.8(2)
O10	B5	O6	B1	-168.4(2)
O11	B11	O13	B13	-16.7(3)
O11	B11	O14	B14	132.28(19)

Atom	Atom	Atom	Atom	Angle/°
O11	B11	O16	B15	-131.3(2)
O11	B12	O12	B13	-4.3(3)
O12	B12	O11	B11	-3.0(3)
O12	B13	O13	B11	11.1(3)
O13	B11	O11	B12	12.6(3)
O13	B11	O14	B14	-108.0(2)
O13	B11	O16	B15	106.2(2)
O13	B13	O12	B12	0.4(3)
O14	B11	O11	B12	130.70(19)
O14	B11	O13	B13	-133.3(2)
O14	B11	O16	B15	-14.3(3)
O14	B14	O15	B15	-4.6(3)
O15	B14	O14	B11	-4.7(3)
O15	B15	O16	B11	6.6(3)
O16	B11	O11	B12	-109.3(2)
O16	B11	O13	B13	104.9(2)
O16	B11	O14	B14	13.3(3)
O16	B15	O15	B14	3.8(3)
O17	B12	O11	B11	176.84(19)
O17	B12	O12	B13	175.9(2)
O18	B13	O12	B12	179.2(2)
O18	B13	O13	B11	-167.8(2)
O19	B14	O14	B11	176.54(19)
O19	B14	O15	B15	174.25(19)
O20	B15	O15	B14	-175.23(19)
O20	B15	O16	B11	-174.5(2)
C1	C2	C3	C4	0.7(3)
C2	C1	C6	C5	-1.6(3)
C2	C1	P1	C7	-96.67(18)
C2	C1	P1	C13	145.03(16)
C2	C1	P1	C19	25.53(19)
C2	C3	C4	C5	-1.4(3)
C3	C4	C5	C6	0.7(4)
C4	C5	C6	C1	0.8(4)
C6	C1	C2	C3	0.8(3)
C6	C1	P1	C7	78.79(19)
C6	C1	P1	C13	-39.51(19)
C6	C1	P1	C19	-159.01(17)
C7	C8	C9	C10	-0.1(3)
C8	C7	C12	C11	0.2(3)
C8	C7	P1	C1	92.88(19)
C8	C7	P1	C13	-147.71(17)
C8	C7	P1	C19	-28.4(2)
C8	C9	C10	C11	0.0(3)
C9	C10	C11	C12	0.2(4)
C10	C11	C12	C7	-0.3(3)
C12	C7	C8	C9	0.1(3)
C12	C7	P1	C1	-82.12(18)
C12	C7	P1	C13	37.29(19)
C12	C7	P1	C19	156.56(16)
C13	C14	C15	C16	1.2(4)
C14	C13	C18	C17	0.6(3)
C14	C13	P1	C1	-27.9(2)
C14	C13	P1	C7	-146.74(17)
C14	C13	P1	C19	92.32(19)
C14	C15	C16	C17	0.6(4)
C15	C16	C17	C18	-1.8(4)
C16	C17	C18	C13	1.2(3)
C18	C13	C14	C15	-1.8(3)
C18	C13	P1	C1	155.49(16)
C18	C13	P1	C7	36.67(19)
C18	C13	P1	C19	-84.27(18)

Atom	Atom	Atom	Atom	Angle/˚
C20	C19	P1	C1	-178.79(14)
C20	C19	P1	C7	-58.00(17)
C20	C19	P1	C13	60.95(17)
C21	C19	P1	C1	58.82(17)
C21	C19	P1	C7	179.61(15)
C21	C19	P1	C13	-61.44(18)
P1	C1	C2	C3	176.25(16)
P1	C1	C6	C5	-177.04(18)
P1	C7	C8	C9	-174.84(16)
P1	C7	C12	C11	175.19(17)
P1	C13	C14	C15	-178.35(17)
P1	C13	C18	C17	177.20(16)
C31	C32	C33	C34	-0.5(3)
C32	C31	C36	C35	1.7(3)
C32	C31	P31	C37	-152.52(17)
C32	C31	P31	C43	-34.8(2)
C32	C31	P31	C49	86.47(19)
C32	C33	C34	C35	1.1(4)
C33	C34	C35	C36	-0.4(4)
C34	C35	C36	C31	-1.0(4)
C36	C31	C32	C33	-0.9(3)
C36	C31	P31	C37	30.7(2)
C36	C31	P31	C43	148.35(17)
C36	C31	P31	C49	-90.34(19)
C37	C38	C39	C40	-0.5(3)
C38	C37	C42	C41	1.3(3)
C38	C37	P31	C31	-146.52(16)
C38	C37	P31	C43	94.81(18)
C38	C37	P31	C49	-26.73(19)
C38	C39	C40	C41	0.6(3)
C39	C40	C41	C42	0.3(3)
C40	C41	C42	C37	-1.2(3)
C42	C37	C38	C39	-0.4(3)
C42	C37	P31	C31	39.81(19)
C42	C37	P31	C43	-78.85(18)
C42	C37	P31	C49	159.61(16)
C43	C44	C45	C46	0.0(3)
C44	C43	C48	C47	-0.5(3)
C44	C43	P31	C31	147.61(17)
C44	C43	P31	C37	-94.01(18)
C44	C43	P31	C49	27.5(2)
C44	C45	C46	C47	-0.2(3)
C45	C46	C47	C48	0.0(3)
C46	C47	C48	C43	0.4(3)
C48	C43	C44	C45	0.3(3)
C48	C43	P31	C31	-38.77(19)
C48	C43	P31	C37	79.61(18)
C48	C43	P31	C49	-158.87(16)
C50	C49	P31	C31	61.19(18)
C50	C49	P31	C37	-58.66(17)
C50	C49	P31	C43	-178.40(15)
C51	C49	P31	C31	-61.17(16)
C51	C49	P31	C37	178.98(14)
C51	C49	P31	C43	59.24(16)
P31	C31	C32	C33	-177.73(17)
P31	C31	C36	C35	178.48(17)
P31	C37	C38	C39	-173.99(16)
P31	C37	C42	C41	174.97(16)
P31	C43	C44	C45	173.84(16)
P31	C43	C48	C47	-174.22(17)

Table S14: Hydrogen Fractional Atomic Coordinates (×10[4]) and Equivalent Isotropic Displacement Parameters (Å[2]×10[3]) for **2010src0454b**. U_{eq} is defined as 1/3 of the trace of the orthogonalised U_{ij}.

Atom	x	y	z	U_{eq}
H7	3372.18	3114.26	3266.71	37
H8A	2375.5	7189.35	2463.1	55
H9A	-2984.18	3371.48	1622.79	41
H10A	-4543.55	4146.1	3287.41	57
H17A	1660.29	8320.37	-3390.73	40
H18A	81.14	9051.02	-1716.59	59
H19A	8024.87	8132.46	-1790.71	41
H20	5600.84	12328.89	-2857.82	50
H2	2738.33	9360.84	-860.01	33
H3	2081.06	11093.92	-952.61	43
H4	1446.83	11704.61	-123.93	43
H5	1363.42	10600.02	795.72	48
H6	1960.55	8858.66	904.66	39
H8	992.87	6932.03	-627.07	28
H9	-1329.56	6201.38	-466.56	35
H10	-2576.27	5782.91	470.24	35
H11	-1534.42	6083.27	1255.65	36
H12	775.94	6822.07	1105.64	30
H14	4737.07	8432.71	745.14	33
H15	6003.32	7743.21	1555.75	40
H16	5836.37	6003.12	2027.46	38
H17	4442.17	4939.83	1681.45	35
H18	3100.32	5619.73	893.85	29
H19	3425.61	7677.85	-875.86	26
H20A	4842.69	6149.85	-823.65	42
H20B	3339.38	5893.88	-406.17	42
H20C	4826.38	5970.21	-133.02	42
H21A	5931.96	7718.01	-266.4	47
H21B	5187.74	8709.04	-662.64	47
H21C	6013.32	7860.08	-956.61	47
H32	1663.65	9350.89	4036.8	33
H33	304.34	9964.48	3244.93	42
H34	-1091.03	8840.24	2940.19	45
H35	-1195.75	7117.6	3440.18	41
H36	115.22	6501.46	4244.46	34
H38	2235.98	5608.19	5797.34	32
H39	3087.24	3922.73	5864.31	40
H40	3814.87	3367.9	5022.77	41
H41	3736.13	4490.38	4107.35	41
H42	2949.53	6193.96	4025.34	32
H44	3770.26	8102.59	5593.95	28
H45	6075.21	8849.56	5446.04	33
H46	7364.76	9267.31	4518.11	36
H47	6377.98	8940.05	3725.87	34
H48	4082.54	8184.29	3863.23	28
H49	1416.42	7301.23	5834.17	26
H50A	-1143.35	7035.75	5931.42	46
H50B	-284.34	6222.16	5618.65	46
H50C	-1095.84	7200.9	5240.55	46
H51A	-71.85	8968.35	5096.21	43
H51B	1404.25	9085.95	5366.37	43
H51C	-86.92	8793.03	5786.52	43
H61A	-250.21	8265.1	-2763.56	41
H61B	-691.84	7464.52	-2958.77	41
H62A	5960.61	8640.33	-3340.39	42
H62B	7446.29	8445.32	-3337.11	42
H63A	5362.57	7546.61	-2088.5	37
H63B	4889.22	6749.75	-2278.75	37
H64A	518.61	8413.44	1938.97	93

Atom	x	y	z	U_{eq}
H64B	-441.32	9203.34	1730.99	93
H65A	-1583.22	7549.21	2154.15	85
H65B	-1273.76	6519.81	2358.7	85
H66A	6526.28	6849.47	2895.27	71
H66B	5608.65	6082.09	3211.08	71
H67A	1325.9	3594.9	1702.74	42
H67B	2818.99	3426.48	1692.98	42

Table S15: Hydrogen Bond information for **2010src0454b**.

D	H	A	d(D-H)/Å	d(H-A)/Å	d(D-A)/Å	D-H-A/deg
O7	H7	O62[1]	0.84	1.87	2.695(2)	168.1
O8	H8A	O20[2]	0.84	1.87	2.685(2)	161.9
O9	H9A	O63[3]	0.84	2.07	2.874(2)	160.8
O10	H10A	O7[4]	0.84	1.90	2.734(2)	170.9
O17	H17A	O61	0.84	2.06	2.871(2)	162.3
O18	H18A	O19[4]	0.84	1.87	2.703(2)	171.4
O20	H20	O66[2]	0.84	1.88	2.703(3)	166.8
O61	H61A	O12	0.87	2.18	2.8776(19)	136.7
O61	H61B	O1[3]	0.87	1.85	2.711(2)	170.1
O62	H62A	O11	0.87	1.93	2.788(2)	167.8
O62	H62B	O61[5]	0.87	2.04	2.840(2)	153.2
O63	H63A	O14	0.87	1.84	2.698(2)	169.7
O63	H63B	O5[3]	0.87	2.21	2.8865(19)	134.5
O64	H64A	O8	0.87	2.15	2.829(3)	134.4
O64	H64B	O18[6]	0.87	2.07	2.936(3)	176.2
O65	H65A	O64	0.87	1.87	2.714(3)	162.9
O65	H65B	O3	0.87	1.94	2.797(2)	170.8
O66	H66A	O65[5]	0.87	1.81	2.679(3)	173.0
O66	H66B	O10[5]	0.87	2.27	3.069(3)	151.8
O67	H67A	O4	0.87	1.91	2.777(2)	171.9
O67	H67B	O63[1]	0.87	2.05	2.856(2)	153.6

––––

[1]1-x,1-y,-z; [2]1-x,2-y,-z; [3]-x,1-y,-z; [4]-1+x,+y,+z; [5]1+x,+y,+z; [6]-x,2-y,-z

Citations for 2

COLLECT (Hooft, R.W.W., 1998)

DENZO (Otwinowski & Minor, 1997)

O.V. Dolomanov and L.J. Bourhis and R.J. Gildea and J.A.K. Howard and H. Puschmann, Olex2: A complete structure solution, refinement and analysis program, *J. Appl. Cryst.*, (2009), **42**, 339-341.

Sheldrick, G.M., A short history of ShelX, *Acta Cryst.*, (2008), **A64**, 339-341.

Sheldrick, G.M., Crystal structure refinement with ShelXL, *Acta Cryst.*, (2015), **C71**, 3-8.

TGA plots for $[MePPh_3][B_5O_6(OH)_4]{\cdot}2H_2O$ (**1**) and $[^iPrPPh_3][B_5O_6(OH)_4]{\cdot}3.5H_2O$ (**2**).

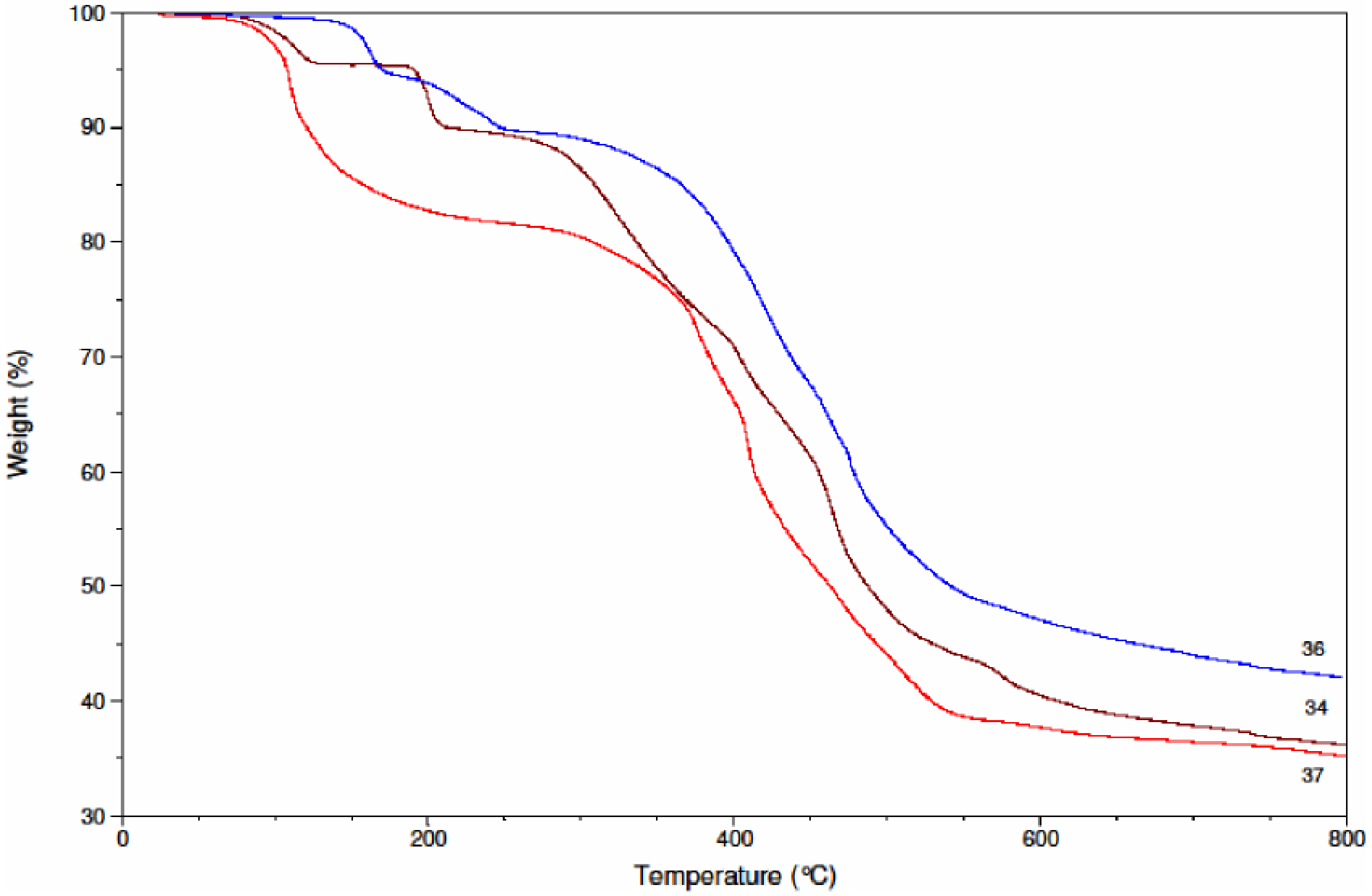

Figure S10. TGA data for the thermal decomposition of tetraalkylphosphonium pentaborate salts. The plot labelled '36' is $[MePPh_3][B_5O_6(OH)_4]{\cdot}2H_2O$ (**1**) that labelled '37' is $[^iPrPPh_3][B_5O_6(OH)_4]{\cdot}3.5H_2O$ (**2**). For comparison the line labelled '34' is $[PPh_4][B_5O_6(OH)_4]{\cdot}1.5H_2O$. (Taken from Ref. 39 in Manuscript).
