# Supplementary material for: Synthesis and Thermal Studies of Two Phosphonium Tetrahydroxidohexaoxidopentaborate(1-) Salts: Single-Crystal XRD Characterization of [iPrPPh3][B5O6(OH)4]·3.5H2O and [MePPh3][B5O6(OH)4]·B(OH)3·0.5H2O
Source: Molecules. 2023 Sep 29;28(19):6867. doi: 10.3390/molecules28196867 (PMC10574587; doi:10.3390/molecules28196867)

checkCIF/PLATON report

Structure factors have been supplied for datablock(s) MAB1

THIS REPORT IS FOR GUIDANCE ONLY. IF USED AS PART OF A REVIEW PROCEDURE FOR PUBLICATION, IT SHOULD NOT REPLACE THE EXPERTISE OF AN EXPERIENCED CRYSTALLOGRAPHIC REFEREE.

No syntax errors found. CIF dictionary Interpreting this report

Datablock: MAB1

Bond precision: C-C = 0.0032 A Wavelength=0.71073

Cell:	a=9.1810(3)	b=13.4812(5)	c=23.8357(8)
	alpha=75.313(1)	beta=83.095(2)	gamma=86.919(2)
Temperature:	120 K		

	Calculated	Reported
Volume	2832.24(17)	2832.24(17)
Space group	P -1	P -1
Hall group	-P 1	-P 1
Moiety formula	2(C21 H22 P), 2(B5 H4 O10), 7(H2 O)	B5 H4 O10, C21 H22 P, 3.5(H2 O)
Sum formula	C42 H66 B10 O27 P2	C21 H33 B5 O13.50 P
Mr	1172.99	586.49
Dx,g cm-3	1.375	1.375
Z	2	4
Mu (mm-1)	0.162	0.162
F000	1228.0	1228.0
F000’	1229.15	
h,k,lmax	11,17,31	11,17,30
Nref	13138	12947
Tmin,Tmax	0.966,0.981	0.959,0.981
Tmin’	0.959	

Correction method= # Reported T Limits: Tmin=0.959 Tmax=0.981
AbsCorr = MULTI-SCAN

Data completeness= 0.985 Theta(max)= 27.605

R(reflections)= 0.0563(9308) wR2(reflections)= 0.1571(12993)

S = 1.038 Npar= 763

The following ALERTS were generated. Each ALERT has the format
test-name_ALERT_alert-type_alert-level.
Click on the hyperlinks for more details of the test.

Alert level B

```
PLAT112_ALERT_2_B ADDSYM Detects New (Pseudo) Symm. Elem          I          98 %Fit
PLAT910_ALERT_3_B Missing # of FCF Reflection(s) Below Theta(Min).           18 Note
```

Alert level C

```
PLAT042_ALERT_1_C Calc. and Reported MoietyFormula Strings  Differ     Please Check
PLAT250_ALERT_2_C Large U3/U1 Ratio for Average U(i,j) Tensor ....        2.8 Note
PLAT250_ALERT_2_C Large U3/U1 Ratio for Average U(i,j) Tensor ....        2.5 Note
PLAT906_ALERT_3_C Large K Value in the Analysis of Variance ......      7.536 Check
PLAT911_ALERT_3_C Missing FCF Refl Between Thmin & STh/L=    0.600         10 Report
PLAT913_ALERT_3_C Missing # of Very Strong Reflections in FCF ....          6 Note
PLAT934_ALERT_3_C Number of (Iobs-Icalc)/Sigma(W) > 10 Outliers ..          1 Check
PLAT975_ALERT_2_C Check Calcd Resid. Dens.  0.90Ang From O64     .       0.55 eA-3
PLAT976_ALERT_2_C Check Calcd Resid. Dens.  0.68Ang From O64     .      -0.49 eA-3
PLAT976_ALERT_2_C Check Calcd Resid. Dens.  0.52Ang From O64     .      -0.49 eA-3
PLAT976_ALERT_2_C Check Calcd Resid. Dens.  0.59Ang From O65     .      -0.43 eA-3
PLAT976_ALERT_2_C Check Calcd Resid. Dens.  0.68Ang From O64     .      -0.43 eA-3
PLAT976_ALERT_2_C Check Calcd Resid. Dens.  0.53Ang From O66     .      -0.41 eA-3
PLAT992_ALERT_5_C Repd & Actual _reflns_number_gt Values Differ by         18 Check
```

Alert level G

```
PLAT002_ALERT_2_G Number of Distance or Angle Restraints on AtSite          1 Note
PLAT007_ALERT_5_G Number of Unrefined Donor-H Atoms .............          22 Report
PLAT045_ALERT_1_G Calculated and Reported Z Differ by a Factor ...      0.500 Check
PLAT066_ALERT_1_G Predicted and Reported Tmin&Tmax Range Identical          ? Check
PLAT172_ALERT_4_G The CIF-Embedded .res File Contains DFIX Records          1 Report
PLAT790_ALERT_4_G Centre of Gravity not Within Unit Cell: Resd.  #          4 Note
              B5 H4 O10
PLAT790_ALERT_4_G Centre of Gravity not Within Unit Cell: Resd.  #          5 Note
              H2 O
PLAT790_ALERT_4_G Centre of Gravity not Within Unit Cell: Resd.  #          6 Note
              H2 O
PLAT790_ALERT_4_G Centre of Gravity not Within Unit Cell: Resd.  #          7 Note
              H2 O
PLAT790_ALERT_4_G Centre of Gravity not Within Unit Cell: Resd.  #          9 Note
              H2 O
PLAT860_ALERT_3_G Number of Least-Squares Restraints ............           1 Note
PLAT883_ALERT_1_G No Info/Value for _atom_sites_solution_primary .     Please Do !
PLAT912_ALERT_4_G Missing # of FCF Reflections Above STh/L=  0.600        112 Note
PLAT933_ALERT_2_G Number of HKL-OMIT Records in Embedded .res File          9 Note
PLAT941_ALERT_3_G Average HKL Measurement Multiplicity ..........         3.9 Low
PLAT978_ALERT_2_G Number C-C Bonds with Positive Residual Density.          2 Info
```

0 **ALERT level A** = Most likely a serious problem - resolve or explain
2 **ALERT level B** = A potentially serious problem, consider carefully
14 **ALERT level C** = Check. Ensure it is not caused by an omission or oversight
16 **ALERT level G** = General information/check it is not something unexpected

```
 4 ALERT type 1 CIF construction/syntax error, inconsistent or missing data
12 ALERT type 2 Indicator that the structure model may be wrong or deficient
 7 ALERT type 3 Indicator that the structure quality may be low
 7 ALERT type 4 Improvement, methodology, query or suggestion
 2 ALERT type 5 Informative message, check
```

It is advisable to attempt to resolve as many as possible of the alerts in all categories. Often the minor alerts point to easily fixed oversights, errors and omissions in your CIF or refinement strategy, so attention to these fine details can be worthwhile. In order to resolve some of the more serious problems it may be necessary to carry out additional measurements or structure refinements. However, the purpose of your study may justify the reported deviations and the more serious of these should normally be commented upon in the discussion or experimental section of a paper or in the "special_details" fields of the CIF. checkCIF was carefully designed to identify outliers and unusual parameters, but every test has its limitations and alerts that are not important in a particular case may appear. Conversely, the absence of alerts does not guarantee there are no aspects of the results needing attention. It is up to the individual to critically assess their own results and, if necessary, seek expert advice.

Publication of your CIF in IUCr journals

A basic structural check has been run on your CIF. These basic checks will be run on all CIFs submitted for publication in IUCr journals (*Acta Crystallographica*, *Journal of Applied Crystallography*, *Journal of Synchrotron Radiation*); however, if you intend to submit to *Acta Crystallographica Section C* or *E* or *IUCrData*, you should make sure that full publication checks are run on the final version of your CIF prior to submission.

Publication of your CIF in other journals

Please refer to the *Notes for Authors* of the relevant journal for any special instructions relating to CIF submission.

PLATON version of 06/07/2023; check.def file version of 30/06/2023

Datablock MAB1 - ellipsoid plot

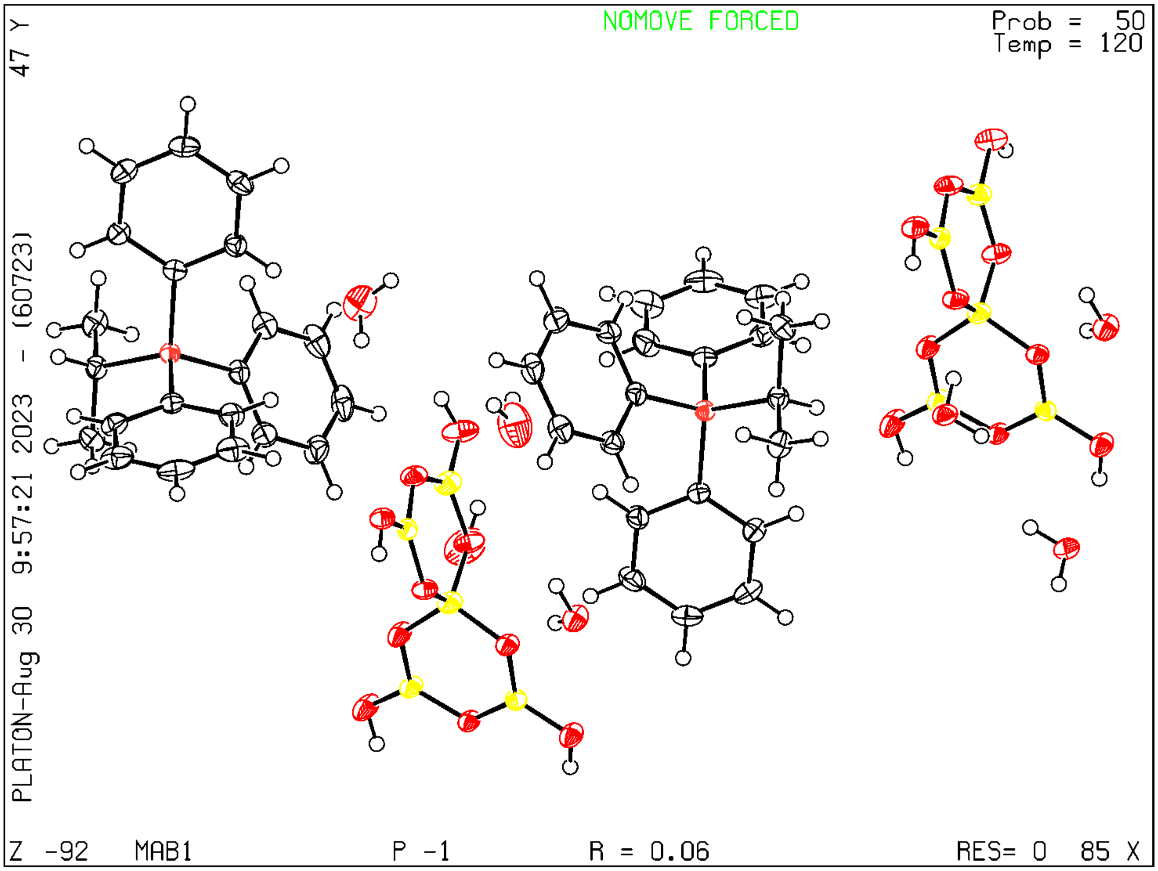

Supplement: Supplementary file 1 [file molecules-28-06867-s001.zip › MAB1_checkcif.pdf]
