# Supplementary material for: Synthesis and Thermal Studies of Two Phosphonium Tetrahydroxidohexaoxidopentaborate(1-) Salts: Single-Crystal XRD Characterization of [iPrPPh3][B5O6(OH)4]·3.5H2O and [MePPh3][B5O6(OH)4]·B(OH)3·0.5H2O
Source: Molecules. 2023 Sep 29;28(19):6867. doi: 10.3390/molecules28196867 (PMC10574587; doi:10.3390/molecules28196867)

checkCIF/PLATON report

Structure factors have been supplied for datablock(s) MAB2

THIS REPORT IS FOR GUIDANCE ONLY. IF USED AS PART OF A REVIEW PROCEDURE FOR PUBLICATION, IT SHOULD NOT REPLACE THE EXPERTISE OF AN EXPERIENCED CRYSTALLOGRAPHIC REFEREE.

No syntax errors found. CIF dictionary Interpreting this report

Datablock: MAB2

Bond precision: C-C = 0.0053 A Wavelength=0.71073

Cell:	a=10.1076(10)	b=13.0403(10)	c=20.6260(15)
	alpha=84.364(5)	beta=82.811(4)	gamma=76.492(4)
Temperature:	120 K		

	Calculated	Reported
Volume	2615.9(4)	2615.9(4)
Space group	P -1	P -1
Hall group	-P 1	-P 1
Moiety formula	2(C19 H18 P), 2(B5 H4 O10), 2(B H3 O3), H2 O	2(B5 H4 O10), 2(C19 H18 P), 2(B H3 O3), H2 O
Sum formula	C38 H52 B12 O27 P2	C38 H52 B12 O27 P2
Mr	1132.46	1132.45
Dx,g cm-3	1.438	1.438
Z	2	2
Mu (mm-1)	0.172	0.172
F000	1172.0	1172.0
F000'	1173.13	
h,k,lmax	13,17,27	13,17,26
Nref	12551	12083
Tmin,Tmax	0.974,0.988	0.966,0.988
Tmin'	0.966	

Correction method= # Reported T Limits: Tmin=0.966 Tmax=0.988
AbsCorr = MULTI-SCAN

Data completeness= 0.963 Theta(max)= 27.938

R(reflections)= 0.0742(9025) wR2(reflections)= 0.1676(12083)

S = 1.040 Npar= 735

The following ALERTS were generated. Each ALERT has the format
test-name_ALERT_alert-type_alert-level.
Click on the hyperlinks for more details of the test.

Alert level B

```
PLAT230_ALERT_2_B Hirshfeld Test Diff for    P21      --C22      .        8.0 s.u.
PLAT910_ALERT_3_B Missing # of FCF Reflection(s) Below Theta(Min).         16 Note
```

Alert level C

```
PLAT042_ALERT_1_C Calc. and Reported MoietyFormula Strings  Differ     Please Check
PLAT250_ALERT_2_C Large U3/U1 Ratio for Average U(i,j) Tensor ....        2.2 Note
PLAT340_ALERT_3_C Low Bond Precision on  C-C Bonds ..............    0.00531 Ang.
PLAT906_ALERT_3_C Large K Value in the Analysis of Variance ......      6.236 Check
PLAT911_ALERT_3_C Missing FCF Refl Between Thmin & STh/L=    0.600         94 Report
```

Alert level G

```
PLAT007_ALERT_5_G Number of Unrefined Donor-H Atoms ..............         22 Report
PLAT066_ALERT_1_G Predicted and Reported Tmin&Tmax Range Identical          ? Check
PLAT083_ALERT_2_G SHELXL Second Parameter in WGHT  Unusually Large       6.86 Why ?
PLAT171_ALERT_4_G The CIF-Embedded .res File Contains EADP Records          1 Report
PLAT302_ALERT_4_G Anion/Solvent/Minor-Residue Disorder (Resd  7  )       100% Note
PLAT302_ALERT_4_G Anion/Solvent/Minor-Residue Disorder (Resd  8  )       100% Note
PLAT302_ALERT_4_G Anion/Solvent/Minor-Residue Disorder (Resd  9  )       100% Note
PLAT302_ALERT_4_G Anion/Solvent/Minor-Residue Disorder (Resd 10  )       100% Note
PLAT304_ALERT_4_G Non-Integer Number of Atoms in ..... (Resd  7  )       0.47 Check
PLAT304_ALERT_4_G Non-Integer Number of Atoms in ..... (Resd  8  )       0.65 Check
PLAT304_ALERT_4_G Non-Integer Number of Atoms in ..... (Resd  9  )       0.84 Check
PLAT304_ALERT_4_G Non-Integer Number of Atoms in ..... (Resd 10  )       1.03 Check
PLAT417_ALERT_2_G Short Inter D-H..H-D       H20      ..H43A     .       1.82 Ang.
                                                      x,y,z  =      1_555 Check
PLAT417_ALERT_2_G Short Inter D-H..H-D       H20      ..H44A     .       1.81 Ang.
                                                      x,y,z  =      1_555 Check
PLAT417_ALERT_2_G Short Inter D-H..H-D       H20      ..H44B     .       2.08 Ang.
                                                      x,y,z  =      1_555 Check
PLAT789_ALERT_4_G Atoms with Negative _atom_site_disorder_group  #         12 Check
PLAT790_ALERT_4_G Centre of Gravity not Within Unit Cell: Resd.  #          5 Note
              B H3 O3
PLAT790_ALERT_4_G Centre of Gravity not Within Unit Cell: Resd.  #          6 Note
              B H3 O3
PLAT822_ALERT_4_G CIF-embedded .res Contains Negative PART Numbers          4 Check
PLAT860_ALERT_3_G Number of Least-Squares Restraints .............          1 Note
PLAT883_ALERT_1_G No Info/Value for _atom_sites_solution_primary .     Please Do !
PLAT912_ALERT_4_G Missing # of FCF Reflections Above STh/L=  0.600        349 Note
PLAT933_ALERT_2_G Number of HKL-OMIT Records in Embedded .res File          1 Note
PLAT941_ALERT_3_G Average HKL Measurement Multiplicity ...........        2.9 Low
PLAT978_ALERT_2_G Number C-C Bonds with Positive Residual Density.          0 Info
```

0 **ALERT level A** = Most likely a serious problem - resolve or explain
2 **ALERT level B** = A potentially serious problem, consider carefully
5 **ALERT level C** = Check. Ensure it is not caused by an omission or oversight
25 **ALERT level G** = General information/check it is not something unexpected

```
 3 ALERT type 1 CIF construction/syntax error, inconsistent or missing data
 8 ALERT type 2 Indicator that the structure model may be wrong or deficient
 6 ALERT type 3 Indicator that the structure quality may be low
14 ALERT type 4 Improvement, methodology, query or suggestion
 1 ALERT type 5 Informative message, check
```

It is advisable to attempt to resolve as many as possible of the alerts in all categories. Often the minor alerts point to easily fixed oversights, errors and omissions in your CIF or refinement strategy, so attention to these fine details can be worthwhile. In order to resolve some of the more serious problems it may be necessary to carry out additional measurements or structure refinements. However, the purpose of your study may justify the reported deviations and the more serious of these should normally be commented upon in the discussion or experimental section of a paper or in the "special_details" fields of the CIF. checkCIF was carefully designed to identify outliers and unusual parameters, but every test has its limitations and alerts that are not important in a particular case may appear. Conversely, the absence of alerts does not guarantee there are no aspects of the results needing attention. It is up to the individual to critically assess their own results and, if necessary, seek expert advice.

Publication of your CIF in IUCr journals

A basic structural check has been run on your CIF. These basic checks will be run on all CIFs submitted for publication in IUCr journals (*Acta Crystallographica*, *Journal of Applied Crystallography*, *Journal of Synchrotron Radiation*); however, if you intend to submit to *Acta Crystallographica Section C* or *E* or *IUCrData*, you should make sure that full publication checks are run on the final version of your CIF prior to submission.

Publication of your CIF in other journals

Please refer to the *Notes for Authors* of the relevant journal for any special instructions relating to CIF submission.

PLATON version of 06/07/2023; check.def file version of 30/06/2023

Datablock MAB2 - ellipsoid plot

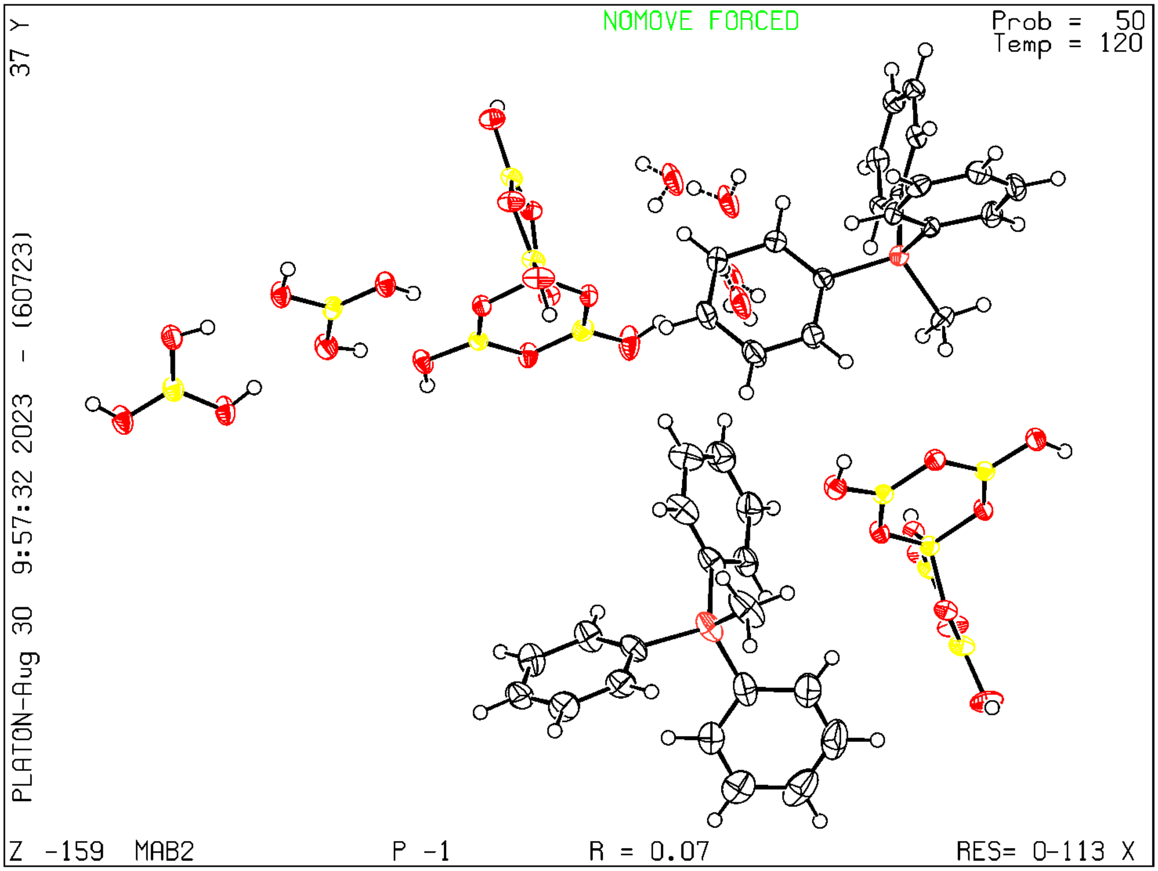

Supplement: Supplementary file 1 [file molecules-28-06867-s001.zip › MAB2_checkcif.pdf]
