# Supplementary material for: Synthesis and Thermal Studies of Two Phosphonium Tetrahydroxidohexaoxidopentaborate(1-) Salts: Single-Crystal XRD Characterization of [iPrPPh3][B5O6(OH)4]·3.5H2O and [MePPh3][B5O6(OH)4]·B(OH)3·0.5H2O
Source: Molecules. 2023 Sep 29;28(19):6867. doi: 10.3390/molecules28196867 (PMC10574587; doi:10.3390/molecules28196867)
Supplement: Supplementary file 8 [file molecules-28-06867-s001.zip › Electronic Supplementary data.odt]

Electronic Supplementary data 

					

Synthesis and thermal studies of two phosphonium tetrahydroxidohexaoxidopentaborate(1-) salts: single-crystal XRD characterization of [iPrPPh3][B5O6(OH)4].3.5H2O and [MePPh3][B5O6(OH)4].B(OH)3.0.5H2O

Michael A. Beckett 1*, Peter N. Horton2, Michael B. Hursthouse,b and James L Timmis,a

1	School of Natural Sciences, Bangor University, Bangor, Gwynedd, LL57 2UW, UK;

2	Chemistry Department, University of Southampton, Southampton, SO17 1BJ, UK;

*	Correspondence: [m.a.beckett@bangor.ac..uk](mailto:m.a.beckett@bangor.ac..uk) 

Contains sc-XRD data and TGA data for [iPrPPh3][B5O6(OH)4].3.5H2O (1)  and [MePPh3][B5O6(OH)4].B(OH)3.0.5H2O (2)

A blue and black rectangle

Description automatically generated

[iPrPPh3][B5O6(OH)4].3.5H2O Compound 1

Submitted by: 	Mike A Beckett

		Bangor University

Solved by: 	Peter N Horton

Sample ID: 	MAB/JT/23

R1=7.42%

Crystal Data and Experimental

A group of colorful molecules

Description automatically generated with medium confidence

Experimental. Single colourless cut block-shaped crystals of 2009src0115 (1) were used as supplied. A suitable crystal with dimensions 0.200 × 0.130 × 0.070 mm3 was selected and mounted on a Bruker-Nonius APEX II CCD camera on k-goniostat diffractometer. The crystal was kept at a steady T = 120(2) K during data collection. The structure was solved with the ShelXS-97 (Sheldrick, 1997a) solution program using direct methods and by using Olex2 1.5-alpha (Dolomanov et al., 2009) as the graphical interface. The model was refined with ShelXL 2018/3 (Sheldrick, 2015) using full matrix least squares minimisation on F2.

Crystal Data. C38H52B12O27P2, Mr = 1132.45, triclinic, P-1 (No. 2), a = 10.1076(10) Å, b = 13.0403(10) Å, c = 20.6260(15) Å, a = 84.364(5)°, b = 82.811(4)°, g = 76.492(4)°, V = 2615.9(4) Å3, T = 120(2) K, Z = 2, Z' = 1, m(MoKa) = 0.172 mm-1, 35616 reflections measured, 12083 unique (Rint = 0.0649) which were used in all calculations. The final wR2 was 0.1676 (all data) and R1 was 0.0742 (I≥2 s(I)).

| Compound 1 | 2009src0115 |
| --- | --- |
|  |  |
| Formula | C38H52B12O27P2 |
| Dcalc./ g cm-3 | 1.438 |
| m/mm-1 | 0.172 |
| Formula Weight | 1132.45 |
| Colour | colourless |
| Shape | cut block-shaped |
| Size/mm3 | 0.200×0.130×0.070 |
| T/K | 120(2) |
| Crystal System | triclinic |
| Space Group | P-1 |
| a/Å | 10.1076(10) |
| b/Å | 13.0403(10) |
| c/Å | 20.6260(15) |
| a/° | 84.364(5) |
| b/° | 82.811(4) |
| g/° | 76.492(4) |
| V/Å3 | 2615.9(4) |
| Z | 2 |
| Z' | 1 |
| Wavelength/Å | 0.71073 |
| Radiation type | MoKa |
| Qmin/° | 2.994 |
| Qmax/° | 27.938 |
| Measured Refl's. | 35616 |
| Indep't Refl's | 12083 |
| Refl's I≥2 s(I) | 9025 |
| Rint | 0.0649 |
| Parameters | 735 |
| Restraints | 1 |
| Largest Peak | 1.103 |
| Deepest Hole | -1.031 |
| GooF | 1.040 |
| wR2 (all data) | 0.1676 |
| wR2 | 0.1485 |
| R1 (all data) | 0.1044 |
| R1 | 0.0742 |

Structure Quality Indicators

| Reflections: |  |
| --- | --- |
| Refinement: |  |

A colourless cut block-shaped-shaped crystal with dimensions 0.200 × 0.130 × 0.070 mm3 was mounted. Data were collected using a Bruker-Nonius APEX II CCD camera on k-goniostat diffractometer equipped with an Oxford Cryosystems low-temperature device operating at T = 120(2) K.

Data were measured using f & w scans with MoKa radiation. The diffraction pattern was indexed and the total number of runs and images was based on the strategy calculation from the program COLLECT (Hooft, R.W.W., 1998). The maximum resolution that was achieved was Q = 27.938° (0.76 Å).

The unit cell was refined using DENZO (Otwinowski & Minor, 1997) & COLLECT (Hooft, R.W.W., 1998) on 1919 reflections, 5% of the observed reflections.

Data reduction, scaling and absorption corrections were performed using DENZO (Otwinowski & Minor, 1997) & COLLECT (Hooft, R.W.W., 1998). The final completeness is 98.80 % out to 27.938° in Q. SADABS 2007/2 (Sheldrick, G.M., 2007).  The absorption coefficient m of this material is 0.172 mm-1 at this wavelength (l = 0.71073Å) and the minimum and maximum transmissions are 0.967 and 0.988.

The structure was solved and the space group P-1 (# 2) determined by the ShelXS-97 (Sheldrick, 1997a) structure solution program using using direct methods and refined by full matrix least squares minimisation on F2 using version 2018/3 of ShelXL 2018/3 (Sheldrick, 2015). All non-hydrogen atoms were refined anisotropically. Hydrogen atom positions were calculated geometrically and refined using the riding model.

_refine_special_details: There was a disordered water molecule over 4 sites (and symmetry related) for which some constraints were applied.

_exptl_absorpt_process_details: SADABS 2007/2 (Sheldrick, G.M., 2007)

There is a single formula unit in the asymmetric unit, which is represented by the reported sum formula. In other words: Z is 2 and Z' is 1. The moiety formula is 2(B5 H4 O10), 2(C19 H18 P), 2(B H3 O3), H2 O.

A close-up of a molecule

Description automatically generated

Figure S1 The following hydrogen bonding interactions with a maximum D-D distance of 2.9 Å and a minimum angle of 120 ° are present in 2009src0115: O7–O31_1: 2.714 Å, O8–O32_2: 2.748 Å, O9–O11_3: 2.828 Å, O10–O6_4: 2.803 Å, O17–O4_5: 2.779 Å, O18–O13_6: 2.764 Å, O19–O7_7: 2.673 Å, O20–O44: 2.622 Å, O20–O43: 2.676 Å, O20–O42_8: 3.057 Å, O21–O19: 2.676 Å, O22–O14: 2.695 Å, O23–O20_5: 2.71 Å, O31–O23: 2.699 Å, O32–O21: 2.671 Å, O33–O1_9: 2.696 Å.

A close up of a structure

Description automatically generated

Figure S2 Packing diagram of 2009src0115 viewed along the a axis

A structure of a molecule

Description automatically generated

Figure S3 Packing diagram of 2009src0115 viewed along the b axis

A close up of a molecule

Description automatically generated

Figure S4 Packing diagram of 2009src0115 viewed along the c axis

Table S1: Fractional Atomic Coordinates (×104) and Equivalent Isotropic Displacement Parameters (Å2×103) for 2009src0115. Ueq is defined as 1/3 of the trace of the orthogonalised Uij.

| Atom | x | y | z | Ueq |
| --- | --- | --- | --- | --- |
| B1 | 724(3) | 9085(2) | 1353.6(16) | 18.1(6) |
| B2 | 496(3) | 10658(3) | 1971.2(15) | 18.8(6) |
| B3 | 2684(3) | 9788(3) | 1527.8(15) | 18.7(6) |
| B4 | -437(4) | 7598(3) | 1487.6(16) | 20.6(7) |
| B5 | -654(4) | 8746(3) | 515.1(17) | 24.0(7) |
| O1 | -71(2) | 9965.8(15) | 1734.1(10) | 19.8(4) |
| O2 | 1879(2) | 10587.2(16) | 1901.3(10) | 21.3(4) |
| O3 | 2159(2) | 9067.2(16) | 1296.2(10) | 20.5(4) |
| O4 | 449(2) | 8100.0(15) | 1688.8(10) | 20.1(4) |
| O5 | -1000(2) | 7903.4(18) | 899.8(10) | 28.4(5) |
| O6 | 255(2) | 9249.4(16) | 696.4(9) | 20.9(4) |
| O7 | -280(2) | 11473.3(17) | 2308.9(11) | 24.3(5) |
| O8 | 4030(2) | 9755.8(18) | 1413.1(11) | 26.6(5) |
| O9 | -835(2) | 6768.2(17) | 1843.6(11) | 27.7(5) |
| O10 | -1242(3) | 9043.2(19) | -48.7(11) | 32.2(5) |
| B11 | 9639(3) | 5489(3) | 3504.2(16) | 18.5(6) |
| B12 | 10908(4) | 6910(3) | 3301.9(16) | 21.7(7) |
| B13 | 10940(3) | 5921(3) | 4339.0(16) | 20.2(7) |
| B14 | 9933(3) | 3771(2) | 3023.0(15) | 17.5(6) |
| B15 | 7713(4) | 4750(3) | 3319.3(17) | 22.6(7) |
| O11 | 10005(2) | 6405.1(15) | 3119.3(10) | 20.1(4) |
| O12 | 11450(2) | 6637.1(17) | 3899.9(10) | 25.8(5) |
| O13 | 9963(2) | 5459.6(15) | 4187.4(9) | 18.9(4) |
| O14 | 10474(2) | 4526.3(15) | 3216.5(10) | 18.6(4) |
| O15 | 8558(2) | 3867.3(16) | 3051.5(11) | 23.1(4) |
| O16 | 8209(2) | 5524.2(15) | 3509.5(10) | 19.4(4) |
| O17 | 11338(3) | 7704.6(18) | 2929.6(11) | 30.8(5) |
| O18 | 11464(2) | 5726.8(17) | 4927.1(10) | 24.4(5) |
| O19 | 10787(2) | 2883.1(16) | 2801.5(11) | 23.1(4) |
| O20 | 6372(2) | 4753.9(19) | 3372.3(14) | 38.8(6) |
| C1 | 1443(3) | 9935(3) | 3597.2(16) | 31.0(7) |
| C2 | 2938(3) | 11214(2) | 4188.1(14) | 21.3(6) |
| C3 | 1791(3) | 11967(2) | 4383.6(16) | 27.8(7) |
| C4 | 1920(4) | 12954(3) | 4517.2(16) | 32.9(8) |
| C5 | 3181(4) | 13189(3) | 4458.9(16) | 30.5(7) |
| C6 | 4316(3) | 12459(3) | 4255.3(15) | 28.2(7) |
| C7 | 4201(3) | 11462(3) | 4117.4(15) | 25.8(7) |
| C8 | 2416(3) | 9300(2) | 4887.8(14) | 21.6(6) |
| C9 | 2448(3) | 9808(2) | 5448.3(15) | 22.4(6) |
| C10 | 2229(3) | 9302(2) | 6060.4(15) | 24.8(6) |
| C11 | 1996(3) | 8290(3) | 6112.9(15) | 24.9(6) |
| C12 | 1956(3) | 7787(3) | 5558.7(16) | 26.4(7) |
| C13 | 2159(3) | 8287(2) | 4942.0(15) | 25.8(6) |
| C14 | 4329(3) | 9167(2) | 3719.8(15) | 23.2(6) |
| C15 | 4619(4) | 9291(3) | 3045.9(16) | 32.9(8) |
| C16 | 5833(4) | 8745(3) | 2753.0(17) | 37.8(9) |
| C17 | 6762(4) | 8091(3) | 3132.7(17) | 33.1(8) |
| C18 | 6479(3) | 7969(3) | 3792.9(17) | 32.9(8) |
| C19 | 5266(3) | 8504(3) | 4091.0(16) | 29.4(7) |
| P1 | 2782.9(8) | 9903.2(6) | 4091.9(4) | 21.23(17) |
| C21 | 5479(5) | 8645(3) | 43(2) | 53.4(12) |
| C22 | 4182(4) | 7103(3) | -416.9(18) | 39.3(9) |
| C23 | 4480(4) | 6349(3) | -883.1(18) | 37.1(8) |
| C24 | 3499(4) | 6210(3) | -1252(2) | 46.2(10) |
| C25 | 2200(5) | 6820(4) | -1160(2) | 63.1(15) |
| C26 | 1896(5) | 7569(4) | -700(2) | 67.0(15) |
| C27 | 2868(4) | 7719(4) | -329(2) | 54.4(12) |
| C28 | 5150(4) | 6788(3) | 878.3(17) | 33.1(8) |
| C29 | 3940(4) | 6475(3) | 1094.6(18) | 34.7(8) |
| C30 | 3696(4) | 6175(3) | 1750.2(19) | 40.7(9) |
| C31 | 4630(4) | 6184(3) | 2178(2) | 43.9(9) |
| C32 | 5839(4) | 6477(3) | 1959.1(19) | 43.9(9) |
| C33 | 6090(4) | 6797(3) | 1305.9(19) | 40.7(9) |
| C34 | 7034(4) | 6566(3) | -307.2(17) | 31.9(7) |
| C35 | 7682(4) | 7027(3) | -852.6(18) | 40.5(9) |
| C36 | 8805(4) | 6438(4) | -1178.9(19) | 49.8(11) |
| C37 | 9298(4) | 5397(4) | -977.0(19) | 45.8(10) |
| C38 | 8674(4) | 4934(3) | -434(2) | 42.7(9) |
| C39 | 7537(4) | 5518(3) | -98.7(19) | 38.5(9) |
| P21 | 5461.1(10) | 7279.1(7) | 50.5(5) | 33.9(2) |
| B21 | 13973(3) | 3538(3) | 2828.6(17) | 21.4(7) |
| O21 | 13483(2) | 2766.1(18) | 2592.5(12) | 30.1(5) |
| O22 | 13187(2) | 4426.4(16) | 3070.1(11) | 24.2(5) |
| O23 | 15345(2) | 3365.8(17) | 2800.9(11) | 26.9(5) |
| B31 | 16446(4) | 1037(3) | 1954.3(18) | 25.0(7) |
| O31 | 16996(2) | 1576.6(17) | 2357.6(11) | 26.5(5) |
| O32 | 15094(2) | 1259.1(18) | 1881.6(12) | 30.3(5) |
| O33 | 17238(2) | 252.4(19) | 1616.4(12) | 33.8(6) |
| O42 | 5273(10) | 4515(7) | 5343(3) | 42.6(17) |
| O41 | 4373(11) | 5477(8) | 5093(3) | 42.6(17) |
| O43 | 4513(4) | 6026(4) | 4149(3) | 42.6(17) |
| O44 | 4456(5) | 6289(4) | 3863(3) | 42.6(17) |

Table S2: Anisotropic Displacement Parameters (×104) for 2009src0115. The anisotropic displacement factor exponent takes the form: -2p2[h2a*2 × U11+ ... +2hka* × b* × U12]

| Atom | U11 | U22 | U33 | U23 | U13 | U12 |
| --- | --- | --- | --- | --- | --- | --- |
| B1 | 19.8(16) | 15.8(15) | 19.2(15) | -2.2(12) | -2.1(12) | -4.5(12) |
| B2 | 20.0(16) | 19.5(15) | 16.8(15) | -1.4(12) | -1.5(12) | -4.3(12) |
| B3 | 19.4(16) | 20.6(15) | 15.8(14) | 1.7(12) | -1.0(12) | -5.7(12) |
| B4 | 24.6(17) | 19.5(16) | 17.8(15) | -2.1(13) | 1.8(12) | -7.0(13) |
| B5 | 31.6(19) | 23.2(17) | 19.8(16) | -2.0(14) | -1.7(14) | -11.9(14) |
| O1 | 16.7(10) | 20.2(10) | 22.8(10) | -6.3(8) | 0.0(8) | -4.0(8) |
| O2 | 17.6(10) | 23.9(10) | 24.6(10) | -6.4(9) | -2.8(8) | -6.9(8) |
| O3 | 17.9(10) | 20.5(10) | 23.2(10) | -5.1(8) | 1.9(8) | -5.2(8) |
| O4 | 21.9(10) | 18.8(10) | 20.2(10) | -1.3(8) | -1.0(8) | -6.4(8) |
| O5 | 37.3(13) | 30.5(12) | 24.7(11) | 3.6(9) | -9.1(9) | -21.8(10) |
| O6 | 25.5(11) | 19.9(10) | 18.5(10) | -2.1(8) | -0.5(8) | -8.5(8) |
| O7 | 17.9(10) | 26.6(11) | 30.0(11) | -11.2(9) | -3.0(9) | -4.4(9) |
| O8 | 17.9(11) | 33.6(12) | 29.9(12) | -6.5(10) | 0.5(9) | -9.0(9) |
| O9 | 35.9(13) | 26.8(11) | 25.1(11) | 2.2(9) | -4.7(10) | -17.2(10) |
| O10 | 47.0(15) | 33.5(13) | 24.2(11) | 4.6(10) | -12.5(10) | -23.7(11) |
| B11 | 19.9(16) | 17.2(15) | 18.4(15) | -0.1(12) | -3.0(12) | -4.3(12) |
| B12 | 24.2(17) | 24.1(17) | 18.3(15) | -3.2(13) | -1.3(13) | -8.3(13) |
| B13 | 21.3(16) | 21.6(16) | 18.4(15) | -3.8(13) | -0.6(12) | -6.2(13) |
| B14 | 18.6(15) | 16.9(15) | 16.9(14) | -1.8(12) | -1.5(12) | -3.5(12) |
| B15 | 20.9(17) | 22.5(16) | 25.8(17) | -0.7(14) | -5.3(13) | -6.2(13) |
| O11 | 22.0(11) | 18.2(10) | 20.7(10) | -0.1(8) | -3.0(8) | -5.9(8) |
| O12 | 28.2(12) | 32.8(12) | 21.1(10) | -1.1(9) | -3.1(9) | -16.4(10) |
| O13 | 22.5(10) | 18.0(10) | 17.7(9) | -1.5(8) | -2.7(8) | -7.3(8) |
| O14 | 16.1(10) | 18.1(10) | 22.5(10) | -4.9(8) | -1.4(8) | -4.6(8) |
| O15 | 16.7(10) | 19.6(10) | 34.5(12) | -5.7(9) | -4.1(9) | -4.6(8) |
| O16 | 15.9(10) | 17.7(10) | 24.4(10) | -3.6(8) | -1.7(8) | -2.5(8) |
| O17 | 43.3(14) | 33.8(12) | 22.3(11) | 1.2(10) | -4.3(10) | -23.8(11) |
| O18 | 27.4(12) | 27.8(12) | 21.1(10) | -0.4(9) | -4.6(9) | -12.1(9) |
| O19 | 20.6(11) | 19.5(10) | 30.2(11) | -9.9(9) | 1.2(9) | -5.1(8) |
| O20 | 16.6(11) | 34.5(14) | 66.6(18) | -14.6(13) | -4.3(11) | -3.8(10) |
| C1 | 24.2(16) | 38.5(19) | 30.3(17) | -2.4(15) | -10.5(13) | -3.1(14) |
| C2 | 21.6(15) | 21.0(14) | 18.1(13) | 1.0(11) | -2.2(11) | 0.6(11) |
| C3 | 25.7(16) | 24.6(16) | 29.7(17) | 2.4(13) | -0.4(13) | -1.6(13) |
| C4 | 34.8(19) | 25.7(16) | 29.6(17) | -0.2(14) | 3.2(14) | 6.5(14) |
| C5 | 42(2) | 21.9(16) | 25.9(16) | -0.5(13) | -4.7(14) | -3.8(14) |
| C6 | 30.2(17) | 30.3(17) | 24.7(16) | 3.7(13) | -6.9(13) | -8.3(14) |
| C7 | 20.7(15) | 28.4(16) | 24.5(15) | -0.7(13) | -3.9(12) | 2.5(12) |
| C8 | 17.7(14) | 23.3(15) | 22.2(14) | -1.0(12) | -1.3(11) | -2.1(11) |
| C9 | 21.0(15) | 20.2(14) | 25.6(15) | -4.1(12) | 0.8(12) | -4.3(11) |
| C10 | 23.7(15) | 26.9(16) | 23.5(15) | -3.7(13) | -0.2(12) | -5.4(12) |
| C11 | 19.9(15) | 30.3(16) | 23.3(15) | 0.8(13) | 1.7(12) | -6.6(12) |
| C12 | 23.1(16) | 24.5(15) | 31.9(17) | -1.1(13) | 1.0(13) | -8.3(12) |
| C13 | 24.7(16) | 28.5(16) | 24.6(15) | -8.1(13) | 0.8(12) | -6.1(13) |
| C14 | 23.8(15) | 21.3(14) | 23.2(15) | -3.7(12) | -0.5(12) | -2.7(12) |
| C15 | 33.5(18) | 31.9(18) | 25.3(16) | 1.5(14) | 1.8(14) | 4.6(14) |
| C16 | 41(2) | 35.9(19) | 27.3(17) | -2.3(15) | 7.2(15) | 3.9(16) |
| C17 | 30.4(18) | 27.7(17) | 35.4(18) | -7.7(14) | 6.1(14) | 2.4(14) |
| C18 | 27.2(17) | 33.3(18) | 32.9(18) | -4.0(15) | -4.9(14) | 5.6(14) |
| C19 | 26.4(17) | 36.1(18) | 22.7(15) | -4.0(14) | -3.3(13) | 0.0(14) |
| P1 | 18.8(4) | 23.6(4) | 19.7(4) | -1.8(3) | -2.1(3) | -1.5(3) |
| C21 | 80(3) | 25.0(18) | 51(2) | -8.4(18) | 26(2) | -19(2) |
| C22 | 40(2) | 28.1(18) | 37.0(19) | 12.3(15) | 14.6(16) | 2.5(15) |
| C23 | 33.5(19) | 31.4(18) | 42(2) | 8.6(16) | 3.8(16) | -7.6(15) |
| C24 | 50(2) | 45(2) | 43(2) | 18.6(19) | -2.0(18) | -19.1(19) |
| C25 | 41(2) | 90(4) | 51(3) | 39(3) | -8(2) | -18(2) |
| C26 | 39(3) | 84(4) | 55(3) | 25(3) | 10(2) | 13(2) |
| C27 | 45(2) | 62(3) | 38(2) | 16(2) | 7.5(18) | 9(2) |
| C28 | 40(2) | 24.6(16) | 31.7(18) | -2.4(14) | 12.0(15) | -10.0(14) |
| C29 | 31.0(18) | 32.4(18) | 36.2(19) | 1.2(15) | 7.3(15) | -5.5(14) |
| C30 | 34(2) | 40(2) | 41(2) | 8.8(17) | 11.9(16) | -7.5(16) |
| C31 | 45(2) | 44(2) | 39(2) | 0.2(18) | 7.4(17) | -9.7(18) |
| C32 | 55(3) | 46(2) | 37(2) | -4.8(18) | -0.9(18) | -24.7(19) |
| C33 | 49(2) | 36(2) | 41(2) | -10.3(17) | 10.9(17) | -21.9(17) |
| C34 | 35.8(19) | 30.4(17) | 30.6(17) | -6.9(14) | 8.1(14) | -13.8(15) |
| C35 | 38(2) | 50(2) | 31.4(19) | 4.8(17) | 3.7(15) | -14.7(17) |
| C36 | 36(2) | 77(3) | 30.3(19) | 9(2) | 8.1(16) | -11(2) |
| C37 | 34(2) | 66(3) | 34(2) | -11.5(19) | 4.0(16) | -6.8(19) |
| C38 | 35(2) | 39(2) | 53(2) | -7.0(18) | 2.4(17) | -7.9(16) |
| C39 | 40(2) | 33.1(19) | 40(2) | -0.2(16) | 10.3(16) | -13.0(16) |
| P21 | 42.3(5) | 22.6(4) | 32.7(5) | -1.0(4) | 12.9(4) | -8.4(4) |
| B21 | 14.5(15) | 23.9(16) | 25.7(17) | -0.1(14) | -1.2(12) | -5.2(13) |
| O21 | 20.8(11) | 34.5(13) | 37.8(13) | -12.3(10) | -1.8(10) | -8.3(10) |
| O22 | 15.7(10) | 25.2(11) | 32.0(12) | -5.0(9) | 0.5(9) | -5.8(8) |
| O23 | 16.0(10) | 29.3(12) | 35.2(13) | -7.5(10) | 0.2(9) | -3.8(9) |
| B31 | 19.3(17) | 26.2(18) | 29.2(18) | -3.6(15) | 0.8(14) | -5.5(13) |
| O31 | 20.7(11) | 27.0(12) | 31.7(12) | -8.8(10) | -0.8(9) | -3.3(9) |
| O32 | 17.5(11) | 29.0(12) | 44.3(14) | -11.8(10) | 1.0(10) | -3.7(9) |
| O33 | 17.3(11) | 38.0(13) | 48.8(15) | -21.2(11) | -0.5(10) | -5.4(10) |
| O42 | 16(2) | 51(3) | 63(5) | -36(3) | 4(3) | -3(2) |
| O41 | 16(2) | 51(3) | 63(5) | -36(3) | 4(3) | -3(2) |
| O43 | 16(2) | 51(3) | 63(5) | -36(3) | 4(3) | -3(2) |
| O44 | 16(2) | 51(3) | 63(5) | -36(3) | 4(3) | -3(2) |

Table S3: Bond Lengths in Å for 2009src0115.

| Atom | Atom | Length/Å |
| --- | --- | --- |
| B1 | O1 | 1.473(4) |  |
| B1 | O3 | 1.435(4) |  |
| B1 | O4 | 1.466(4) |  |
| B1 | O6 | 1.471(4) |  |
| B2 | O1 | 1.335(4) |  |
| B2 | O2 | 1.369(4) |  |
| B2 | O7 | 1.360(4) |  |
| B3 | O2 | 1.395(4) |  |
| B3 | O3 | 1.334(4) |  |
| B3 | O8 | 1.342(4) |  |
| B4 | O4 | 1.350(4) |  |
| B4 | O5 | 1.387(4) |  |
| B4 | O9 | 1.359(4) |  |
| B5 | O5 | 1.378(4) |  |
| B5 | O6 | 1.353(4) |  |
| B5 | O10 | 1.353(4) |  |
| B11 | O11 | 1.463(4) |  |
| B11 | O13 | 1.481(4) |  |
| B11 | O14 | 1.472(4) |  |
| B11 | O16 | 1.434(4) |  |
| B12 | O11 | 1.351(4) |  |
| B12 | O12 | 1.393(4) |  |
| B12 | O17 | 1.350(4) |  |
| B13 | O12 | 1.378(4) |  |
| B13 | O13 | 1.351(4) |  |
| B13 | O18 | 1.361(4) |  |
| B14 | O14 | 1.346(4) |  |
| B14 | O15 | 1.361(4) |  |
| B14 | O19 | 1.354(4) |  |
| B15 | O15 | 1.381(4) |  |
| B15 | O16 | 1.337(4) |  |
| B15 | O20 | 1.346(4) |  |
| C1 | P1 | 1.785(3) |  |
| C2 | C3 | 1.379(4) |  |
| C2 | C7 | 1.375(4) |  |
| C2 | P1 | 1.785(3) |  |
| C3 | C4 | 1.380(5) |  |
| C4 | C5 | 1.368(5) |  |
| C5 | C6 | 1.359(5) |  |
| C6 | C7 | 1.392(5) |  |
| C8 | C9 | 1.395(4) |  |
| C8 | C13 | 1.396(4) |  |
| C8 | P1 | 1.784(3) |  |
| C9 | C10 | 1.383(4) |  |
| C10 | C11 | 1.386(4) |  |
| C11 | C12 | 1.382(4) |  |
| C12 | C13 | 1.386(4) |  |
| C14 | C15 | 1.386(4) |  |
| C14 | C19 | 1.378(4) |  |
| C14 | P1 | 1.760(3) |  |
| C15 | C16 | 1.367(5) |  |
| C16 | C17 | 1.378(5) |  |
| C17 | C18 | 1.357(5) |  |
| C18 | C19 | 1.365(4) |  |
| C21 | P21 | 1.784(4) |  |
| C22 | C23 | 1.399(5) |  |
| C22 | C27 | 1.382(5) |  |
| C22 | P21 | 1.774(5) |  |
| C23 | C24 | 1.376(6) |  |
| C24 | C25 | 1.368(6) |  |
| C25 | C26 | 1.385(8) |  |
| C26 | C27 | 1.377(7) |  |
| C28 | C29 | 1.386(5) |  |
| C28 | C33 | 1.378(5) |  |
| C28 | P21 | 1.780(3) |  |
| C29 | C30 | 1.379(5) |  |
| C30 | C31 | 1.373(6) |  |
| C31 | C32 | 1.375(5) |  |
| C32 | C33 | 1.381(5) |  |
| C34 | C35 | 1.387(5) |  |
| C34 | C39 | 1.387(5) |  |
| C34 | P21 | 1.757(4) |  |
| C35 | C36 | 1.355(5) |  |
| C36 | C37 | 1.374(6) |  |
| C37 | C38 | 1.373(5) |  |
| C38 | C39 | 1.369(5) |  |
| B21 | O21 | 1.373(4) |  |
| B21 | O22 | 1.344(4) |  |
| B21 | O23 | 1.347(4) |  |
| B31 | O31 | 1.382(4) |  |
| B31 | O32 | 1.354(4) |  |
| B31 | O33 | 1.339(4) |  |

Table S4: Bond Angles in ° for 2009src0115.

| Atom | Atom | Atom | Angle/° |
| --- | --- | --- | --- |
| O3 | B1 | O1 | 110.9(2) |  |
| O3 | B1 | O4 | 110.7(2) |  |
| O3 | B1 | O6 | 109.2(2) |  |
| O4 | B1 | O1 | 107.8(2) |  |
| O4 | B1 | O6 | 110.7(2) |  |
| O6 | B1 | O1 | 107.5(2) |  |
| O1 | B2 | O2 | 122.7(3) |  |
| O1 | B2 | O7 | 121.3(3) |  |
| O7 | B2 | O2 | 116.0(3) |  |
| O3 | B3 | O2 | 122.1(3) |  |
| O3 | B3 | O8 | 120.3(3) |  |
| O8 | B3 | O2 | 117.6(3) |  |
| O4 | B4 | O5 | 121.7(3) |  |
| O4 | B4 | O9 | 122.6(3) |  |
| O9 | B4 | O5 | 115.8(3) |  |
| O6 | B5 | O5 | 120.6(3) |  |
| O6 | B5 | O10 | 121.5(3) |  |
| O10 | B5 | O5 | 117.9(3) |  |
| B2 | O1 | B1 | 123.2(2) |  |
| B2 | O2 | B3 | 116.7(2) |  |
| B3 | O3 | B1 | 124.1(2) |  |
| B4 | O4 | B1 | 122.3(2) |  |
| B5 | O5 | B4 | 119.0(3) |  |
| B5 | O6 | B1 | 123.1(2) |  |
| O11 | B11 | O13 | 110.4(2) |  |
| O11 | B11 | O14 | 108.2(2) |  |
| O14 | B11 | O13 | 107.7(2) |  |
| O16 | B11 | O11 | 110.8(2) |  |
| O16 | B11 | O13 | 109.0(2) |  |
| O16 | B11 | O14 | 110.7(2) |  |
| O11 | B12 | O12 | 121.1(3) |  |
| O17 | B12 | O11 | 123.2(3) |  |
| O17 | B12 | O12 | 115.7(3) |  |
| O13 | B13 | O12 | 121.0(3) |  |
| O13 | B13 | O18 | 122.9(3) |  |
| O18 | B13 | O12 | 116.1(3) |  |
| O14 | B14 | O15 | 122.1(3) |  |
| O14 | B14 | O19 | 118.8(3) |  |
| O19 | B14 | O15 | 119.1(3) |  |
| O16 | B15 | O15 | 121.9(3) |  |
| O16 | B15 | O20 | 123.7(3) |  |
| O20 | B15 | O15 | 114.3(3) |  |
| B12 | O11 | B11 | 122.3(2) |  |
| B13 | O12 | B12 | 119.0(2) |  |
| B13 | O13 | B11 | 121.6(2) |  |
| B14 | O14 | B11 | 123.1(2) |  |
| B14 | O15 | B15 | 117.8(2) |  |
| B15 | O16 | B11 | 124.2(2) |  |
| C3 | C2 | P1 | 119.6(2) |  |
| C7 | C2 | C3 | 119.4(3) |  |
| C7 | C2 | P1 | 120.8(2) |  |
| C2 | C3 | C4 | 119.9(3) |  |
| C5 | C4 | C3 | 120.4(3) |  |
| C6 | C5 | C4 | 120.1(3) |  |
| C5 | C6 | C7 | 120.0(3) |  |
| C2 | C7 | C6 | 120.0(3) |  |
| C9 | C8 | C13 | 120.3(3) |  |
| C9 | C8 | P1 | 120.8(2) |  |
| C13 | C8 | P1 | 118.8(2) |  |
| C10 | C9 | C8 | 119.8(3) |  |
| C9 | C10 | C11 | 119.8(3) |  |
| C12 | C11 | C10 | 120.6(3) |  |
| C11 | C12 | C13 | 120.3(3) |  |
| C12 | C13 | C8 | 119.2(3) |  |
| C15 | C14 | P1 | 119.0(2) |  |
| C19 | C14 | C15 | 120.1(3) |  |
| C19 | C14 | P1 | 120.9(2) |  |
| C16 | C15 | C14 | 119.5(3) |  |
| C15 | C16 | C17 | 119.6(3) |  |
| C18 | C17 | C16 | 120.9(3) |  |
| C17 | C18 | C19 | 120.1(3) |  |
| C18 | C19 | C14 | 119.8(3) |  |
| C2 | P1 | C1 | 110.34(15) |  |
| C8 | P1 | C1 | 110.33(15) |  |
| C8 | P1 | C2 | 107.74(14) |  |
| C14 | P1 | C1 | 109.16(16) |  |
| C14 | P1 | C2 | 110.16(14) |  |
| C14 | P1 | C8 | 109.09(14) |  |
| C23 | C22 | P21 | 121.0(3) |  |
| C27 | C22 | C23 | 118.7(4) |  |
| C27 | C22 | P21 | 120.3(3) |  |
| C24 | C23 | C22 | 121.5(4) |  |
| C25 | C24 | C23 | 119.3(5) |  |
| C24 | C25 | C26 | 119.5(5) |  |
| C27 | C26 | C25 | 121.8(4) |  |
| C26 | C27 | C22 | 119.1(5) |  |
| C29 | C28 | P21 | 120.3(3) |  |
| C33 | C28 | C29 | 121.0(3) |  |
| C33 | C28 | P21 | 118.5(3) |  |
| C30 | C29 | C28 | 118.4(4) |  |
| C31 | C30 | C29 | 120.8(4) |  |
| C30 | C31 | C32 | 120.7(4) |  |
| C31 | C32 | C33 | 119.3(4) |  |
| C28 | C33 | C32 | 119.8(4) |  |
| C35 | C34 | C39 | 120.3(3) |  |
| C35 | C34 | P21 | 118.6(3) |  |
| C39 | C34 | P21 | 120.6(3) |  |
| C36 | C35 | C34 | 119.0(4) |  |
| C35 | C36 | C37 | 120.8(4) |  |
| C38 | C37 | C36 | 120.7(4) |  |
| C39 | C38 | C37 | 119.2(4) |  |
| C38 | C39 | C34 | 120.0(3) |  |
| C22 | P21 | C21 | 110.8(2) |  |
| C22 | P21 | C28 | 110.34(17) |  |
| C28 | P21 | C21 | 108.37(18) |  |
| C34 | P21 | C21 | 110.39(19) |  |
| C34 | P21 | C22 | 106.82(17) |  |
| C34 | P21 | C28 | 110.10(17) |  |
| O22 | B21 | O21 | 124.7(3) |  |
| O22 | B21 | O23 | 119.7(3) |  |
| O23 | B21 | O21 | 115.6(3) |  |
| O32 | B31 | O31 | 122.6(3) |  |
| O33 | B31 | O31 | 121.0(3) |  |
| O33 | B31 | O32 | 116.4(3) |  |

Table S5: Torsion Angles in ° for 2009src0115.

| Atom | Atom | Atom | Atom | Angle/° |  |
| --- | --- | --- | --- | --- | --- |
| O1 | B1 | O3 | B3 | -3.3(4) |
| O1 | B1 | O4 | B4 | 100.5(3) |
| O1 | B1 | O6 | B5 | -99.0(3) |
| O1 | B2 | O2 | B3 | 4.2(4) |
| O2 | B2 | O1 | B1 | -2.4(4) |
| O2 | B3 | O3 | B1 | 5.7(4) |
| O3 | B1 | O1 | B2 | 1.7(4) |
| O3 | B1 | O4 | B4 | -138.0(3) |
| O3 | B1 | O6 | B5 | 140.6(3) |
| O3 | B3 | O2 | B2 | -5.7(4) |
| O4 | B1 | O1 | B2 | 123.1(3) |
| O4 | B1 | O3 | B3 | -123.0(3) |
| O4 | B1 | O6 | B5 | 18.5(4) |
| O4 | B4 | O5 | B5 | -0.6(5) |
| O5 | B4 | O4 | B1 | 9.0(4) |
| O5 | B5 | O6 | B1 | -12.2(5) |
| O6 | B1 | O1 | B2 | -117.6(3) |
| O6 | B1 | O3 | B3 | 114.9(3) |
| O6 | B1 | O4 | B4 | -16.8(4) |
| O6 | B5 | O5 | B4 | 2.1(5) |
| O7 | B2 | O1 | B1 | 178.6(3) |
| O7 | B2 | O2 | B3 | -176.8(2) |
| O8 | B3 | O2 | B2 | 175.2(3) |
| O8 | B3 | O3 | B1 | -175.3(3) |
| O9 | B4 | O4 | B1 | -171.0(3) |
| O9 | B4 | O5 | B5 | 179.4(3) |
| O10 | B5 | O5 | B4 | -178.5(3) |
| O10 | B5 | O6 | B1 | 168.5(3) |
| O11 | B11 | O13 | B13 | -25.2(4) |
| O11 | B11 | O14 | B14 | -125.0(3) |
| O11 | B11 | O16 | B15 | 124.3(3) |
| O11 | B12 | O12 | B13 | -7.1(4) |
| O12 | B12 | O11 | B11 | -5.6(4) |
| O12 | B13 | O13 | B11 | 15.1(4) |
| O13 | B11 | O11 | B12 | 20.4(4) |
| O13 | B11 | O14 | B14 | 115.8(3) |
| O13 | B11 | O16 | B15 | -114.1(3) |
| O13 | B13 | O12 | B12 | 2.3(4) |
| O14 | B11 | O11 | B12 | -97.1(3) |
| O14 | B11 | O13 | B13 | 92.7(3) |
| O14 | B11 | O16 | B15 | 4.2(4) |
| O14 | B14 | O15 | B15 | -3.3(4) |
| O15 | B14 | O14 | B11 | 3.2(4) |
| O15 | B15 | O16 | B11 | -4.9(5) |
| O16 | B11 | O11 | B12 | 141.3(3) |
| O16 | B11 | O13 | B13 | -147.1(3) |
| O16 | B11 | O14 | B14 | -3.3(4) |
| O16 | B15 | O15 | B14 | 4.1(4) |
| O17 | B12 | O11 | B11 | 174.8(3) |
| O17 | B12 | O12 | B13 | 172.5(3) |
| O18 | B13 | O12 | B12 | -176.8(3) |
| O18 | B13 | O13 | B11 | -165.8(3) |
| O19 | B14 | O14 | B11 | -176.0(2) |
| O19 | B14 | O15 | B15 | 175.8(3) |
| O20 | B15 | O15 | B14 | -175.6(3) |
| O20 | B15 | O16 | B11 | 174.8(3) |
| C2 | C3 | C4 | C5 | -0.2(5) |
| C3 | C2 | C7 | C6 | 1.3(5) |
| C3 | C2 | P1 | C1 | 50.2(3) |
| C3 | C2 | P1 | C8 | -70.3(3) |
| C3 | C2 | P1 | C14 | 170.8(2) |
| C3 | C4 | C5 | C6 | 1.4(5) |
| C4 | C5 | C6 | C7 | -1.2(5) |
| C5 | C6 | C7 | C2 | -0.1(5) |
| C7 | C2 | C3 | C4 | -1.1(5) |
| C7 | C2 | P1 | C1 | -134.7(3) |
| C7 | C2 | P1 | C8 | 104.8(3) |
| C7 | C2 | P1 | C14 | -14.1(3) |
| C8 | C9 | C10 | C11 | 0.7(5) |
| C9 | C8 | C13 | C12 | -1.0(5) |
| C9 | C8 | P1 | C1 | -127.6(3) |
| C9 | C8 | P1 | C2 | -7.0(3) |
| C9 | C8 | P1 | C14 | 112.5(3) |
| C9 | C10 | C11 | C12 | -1.1(5) |
| C10 | C11 | C12 | C13 | 0.4(5) |
| C11 | C12 | C13 | C8 | 0.6(5) |
| C13 | C8 | C9 | C10 | 0.3(4) |
| C13 | C8 | P1 | C1 | 55.4(3) |
| C13 | C8 | P1 | C2 | 175.9(2) |
| C13 | C8 | P1 | C14 | -64.5(3) |
| C14 | C15 | C16 | C17 | -1.0(6) |
| C15 | C14 | C19 | C18 | 0.0(5) |
| C15 | C14 | P1 | C1 | 40.7(3) |
| C15 | C14 | P1 | C2 | -80.6(3) |
| C15 | C14 | P1 | C8 | 161.3(3) |
| C15 | C16 | C17 | C18 | 1.1(6) |
| C16 | C17 | C18 | C19 | -0.6(6) |
| C17 | C18 | C19 | C14 | 0.1(5) |
| C19 | C14 | C15 | C16 | 0.5(5) |
| C19 | C14 | P1 | C1 | -141.6(3) |
| C19 | C14 | P1 | C2 | 97.1(3) |
| C19 | C14 | P1 | C8 | -21.0(3) |
| P1 | C2 | C3 | C4 | 174.1(2) |
| P1 | C2 | C7 | C6 | -173.9(2) |
| P1 | C8 | C9 | C10 | -176.7(2) |
| P1 | C8 | C13 | C12 | 176.1(2) |
| P1 | C14 | C15 | C16 | 178.2(3) |
| P1 | C14 | C19 | C18 | -177.7(3) |
| C22 | C23 | C24 | C25 | -0.3(5) |
| C23 | C22 | C27 | C26 | 0.2(6) |
| C23 | C22 | P21 | C21 | 131.4(3) |
| C23 | C22 | P21 | C28 | -108.5(3) |
| C23 | C22 | P21 | C34 | 11.2(3) |
| C23 | C24 | C25 | C26 | 0.3(6) |
| C24 | C25 | C26 | C27 | 0.0(7) |
| C25 | C26 | C27 | C22 | -0.2(7) |
| C27 | C22 | C23 | C24 | 0.1(5) |
| C27 | C22 | P21 | C21 | -48.1(3) |
| C27 | C22 | P21 | C28 | 71.9(3) |
| C27 | C22 | P21 | C34 | -168.4(3) |
| C28 | C29 | C30 | C31 | -0.1(6) |
| C29 | C28 | C33 | C32 | 0.9(6) |
| C29 | C28 | P21 | C21 | 113.0(3) |
| C29 | C28 | P21 | C22 | -8.5(3) |
| C29 | C28 | P21 | C34 | -126.2(3) |
| C29 | C30 | C31 | C32 | -1.0(6) |
| C30 | C31 | C32 | C33 | 2.1(6) |
| C31 | C32 | C33 | C28 | -2.0(6) |
| C33 | C28 | C29 | C30 | 0.2(5) |
| C33 | C28 | P21 | C21 | -62.5(4) |
| C33 | C28 | P21 | C22 | 176.0(3) |
| C33 | C28 | P21 | C34 | 58.3(3) |
| C34 | C35 | C36 | C37 | 0.3(6) |
| C35 | C34 | C39 | C38 | -0.3(6) |
| C35 | C34 | P21 | C21 | -38.8(4) |
| C35 | C34 | P21 | C22 | 81.8(3) |
| C35 | C34 | P21 | C28 | -158.4(3) |
| C35 | C36 | C37 | C38 | -1.0(7) |
| C36 | C37 | C38 | C39 | 1.0(6) |
| C37 | C38 | C39 | C34 | -0.4(6) |
| C39 | C34 | C35 | C36 | 0.3(6) |
| C39 | C34 | P21 | C21 | 148.8(3) |
| C39 | C34 | P21 | C22 | -90.6(3) |
| C39 | C34 | P21 | C28 | 29.2(4) |
| P21 | C22 | C23 | C24 | -179.5(3) |
| P21 | C22 | C27 | C26 | 179.7(3) |
| P21 | C28 | C29 | C30 | -175.2(3) |
| P21 | C28 | C33 | C32 | 176.4(3) |
| P21 | C34 | C35 | C36 | -172.1(3) |
| P21 | C34 | C39 | C38 | 172.0(3) |

Table S6: Hydrogen Fractional Atomic Coordinates (×104) and Equivalent Isotropic Displacement Parameters (Å2×103) for 2009src0115. Ueq is defined as 1/3 of the trace of the orthogonalised Uij.

| Atom | x | y | z | Ueq |
| --- | --- | --- | --- | --- |
| H7A | -1112.79 | 11488.25 | 2298.23 | 36 |
| H8 | 4210.07 | 10248.46 | 1594.89 | 40 |
| H9A | -519.68 | 6680.75 | 2207.91 | 42 |
| H10A | -995.22 | 9584.16 | -229.1 | 48 |
| H17A | 11015.91 | 7783.89 | 2567.19 | 46 |
| H18A | 11038.96 | 5333.93 | 5175.51 | 37 |
| H19A | 10329.56 | 2504.16 | 2663.73 | 35 |
| H20 | 5927.23 | 5261.56 | 3587.34 | 58 |
| H1A | 1607.21 | 10335.29 | 3178.91 | 47 |
| H1B | 564.2 | 10276.11 | 3825.33 | 47 |
| H1C | 1422.76 | 9211.34 | 3515.84 | 47 |
| H3 | 912.79 | 11807.62 | 4426.29 | 33 |
| H4 | 1127.52 | 13472.16 | 4650.41 | 39 |
| H5 | 3263.55 | 13865.04 | 4561.05 | 37 |
| H6 | 5189.11 | 12629.59 | 4207.04 | 34 |
| H7 | 4995 | 10951.94 | 3974.35 | 31 |
| H9 | 2618.47 | 10498.5 | 5409.75 | 27 |
| H10 | 2239.35 | 9647.88 | 6443.73 | 30 |
| H11 | 1861.75 | 7939.11 | 6533.67 | 30 |
| H12 | 1788.54 | 7094.92 | 5600.74 | 32 |
| H13 | 2124.09 | 7945.34 | 4560.62 | 31 |
| H15 | 3978.18 | 9751.42 | 2789.24 | 39 |
| H16 | 6035.52 | 8814.98 | 2290.61 | 45 |
| H17 | 7612.26 | 7721.59 | 2929.65 | 40 |
| H18 | 7125.68 | 7511.46 | 4047.86 | 39 |
| H19 | 5067.96 | 8419.55 | 4553.05 | 35 |
| H21A | 6268.64 | 8717.24 | 252.33 | 80 |
| H21B | 4634.7 | 9019.69 | 284.09 | 80 |
| H21C | 5544.1 | 8948.8 | -410.21 | 80 |
| H23 | 5380.05 | 5921.94 | -946.22 | 44 |
| H24 | 3722.1 | 5696.06 | -1567.88 | 55 |
| H25 | 1512.42 | 6731.37 | -1410.19 | 76 |
| H26 | 992.51 | 7989.81 | -637.82 | 80 |
| H27 | 2639.4 | 8238.01 | -16.22 | 65 |
| H29 | 3294.75 | 6468.29 | 798.22 | 42 |
| H30 | 2871.45 | 5959.01 | 1907.84 | 49 |
| H31 | 4438.54 | 5985.48 | 2629.34 | 53 |
| H32 | 6494.95 | 6460.3 | 2254.41 | 53 |
| H33 | 6909.33 | 7023.15 | 1151.83 | 49 |
| H35 | 7342.66 | 7745.04 | -995.34 | 49 |
| H36 | 9257.81 | 6748 | -1551.97 | 60 |
| H37 | 10078.4 | 4993.24 | -1215.88 | 55 |
| H38 | 9027.03 | 4217.89 | -292.32 | 51 |
| H39 | 7092.72 | 5205.8 | 276.42 | 46 |
| H21 | 12624.43 | 2909.15 | 2655.25 | 45 |
| H22 | 12360.03 | 4399.29 | 3095.89 | 36 |
| H23A | 15555.99 | 3850.15 | 2977.92 | 40 |
| H31A | 16409.22 | 2114.66 | 2474.72 | 40 |
| H32A | 14678.06 | 1739.48 | 2124.35 | 45 |
| H33A | 18058.15 | 188.11 | 1681.92 | 51 |
| H42A | 5708.8 | 3932.47 | 5535.41 | 64 |
| H42B | 5778.84 | 4594.33 | 4976.19 | 64 |
| H41A | 4541.8 | 6008.2 | 5266.78 | 64 |
| H41B | 4969.58 | 4931.09 | 5232.7 | 64 |
| H43A | 4175.24 | 5569.79 | 3979.9 | 64 |
| H43B | 4223.46 | 6620.77 | 3926.08 | 64 |
| H44A | 4825.93 | 6021.65 | 4218.28 | 64 |
| H44B | 3863.36 | 5914.3 | 3828.7 | 64 |

Table S7: Hydrogen Bond information for 2009src0115.

| D | H | A | d(D-H)/Å | d(H-A)/Å | d(D-A)/Å | D-H-A/deg |
| --- | --- | --- | --- | --- | --- | --- |
| O7 | H7A | O311 | 0.84 | 1.88 | 2.714(3) | 174.2 |
| O8 | H8 | O322 | 0.84 | 1.93 | 2.748(3) | 164.7 |
| O9 | H9A | O113 | 0.84 | 1.99 | 2.828(3) | 172.3 |
| O10 | H10A | O64 | 0.84 | 1.97 | 2.803(3) | 174.1 |
| O17 | H17A | O45 | 0.84 | 1.95 | 2.779(3) | 171.8 |
| O18 | H18A | O136 | 0.84 | 1.93 | 2.764(3) | 174.0 |
| O19 | H19A | O77 | 0.84 | 1.85 | 2.673(3) | 166.7 |
| O20 | H20 | O428 | 0.84 | 2.39 | 3.057(9) | 136.7 |
| O20 | H20 | O43 | 0.84 | 1.88 | 2.676(5) | 158.2 |
| O20 | H20 | O44 | 0.84 | 1.83 | 2.622(5) | 157.5 |
| O21 | H21 | O19 | 0.84 | 1.85 | 2.676(3) | 166.5 |
| O22 | H22 | O14 | 0.84 | 1.86 | 2.695(3) | 171.4 |
| O23 | H23A | O205 | 0.84 | 1.88 | 2.710(3) | 168.7 |
| O31 | H31A | O23 | 0.84 | 1.87 | 2.699(3) | 170.2 |
| O32 | H32A | O21 | 0.84 | 1.84 | 2.671(3) | 169.4 |
| O33 | H33A | O19 | 0.84 | 1.86 | 2.696(3) | 173.2 |

–––– 

1-2+x,1+y,+z; 2-1+x,1+y,+z; 3-1+x,+y,+z; 4-x,2-y,-z; 51+x,+y,+z; 62-x,1-y,1-z; 71+x,-1+y,+z; 81-x,1-y,1-z; 92+x,-1+y,+z

Table S8: Atomic Occupancies for all atoms that are not fully occupied in 2009src0115.

| Atom | Occupancy |
| --- | --- |
| O42 | 0.217(3) |
| H42A | 0.217(3) |
| H42B | 0.217(3) |
| O41 | 0.158(3) |
| H41A | 0.158(3) |
| H41B | 0.158(3) |
| O43 | 0.281(3) |
| H43A | 0.281(3) |
| H43B | 0.281(3) |
| O44 | 0.344(3) |
| H44A | 0.344(3) |
| H44B | 0.344(3) |

Citations for 1

COLLECT (Hooft, R.W.W., 1998)

DENZO (Otwinowski & Minor, 1997)

O.V. Dolomanov and L.J. Bourhis and R.J. Gildea and J.A.K. Howard and H. Puschmann, Olex2: A complete structure solution, refinement and analysis program, J. Appl. Cryst., (2009), 42, 339-341.

Sheldrick, G.M., A short history of ShelX, Acta Cryst., (2008), A64, 339-341.

Sheldrick, G.M., Crystal structure refinement with ShelXL, Acta Cryst., (2015), C71, 3-8.

A blue and black rectangle

Description automatically generated2010src0454b

[MePPh3][B5O6(OH)4].B(OH)3.0.5H2O   Compound 2

Submitted by: 	Mike A Beckett

		Bangor University

Solved by: 	Peter N Horton

Sample ID: 	MAB/JT/33

R1=5.63%

Crystal Data and Experimental

A close-up of a molecule

Description automatically generated

Experimental. Single colourless block-shaped crystals of 2010src0454b  (2) were used as supplied. A suitable crystal with dimensions 0.260 × 0.180 × 0.120 mm3 was selected and mounted on a Damien diffractometer. The crystal was kept at a steady T = 120(2) K during data collection. The structure was solved with the ShelXS-97 (Sheldrick, 2008) solution program using direct methods and by using Olex2 1.5-alpha (Dolomanov et al., 2009) as the graphical interface. The model was refined with ShelXL 2018/3 (Sheldrick, 2015) using full matrix least squares minimisation on F2.

Crystal Data. C21H33B5O13.5P, Mr = 586.49, triclinic, P-1 (No. 2), a = 9.1810(3) Å, b = 13.4812(5) Å, c = 23.8357(8) Å, a = 75.3130(10)°, b = 83.095(2)°, g = 86.919(2)°, V = 2832.24(17) Å3, T = 120(2) K, Z = 4, Z' = 2, m(MoKa) = 0.162 mm-1, 50870 reflections measured, 12947 unique (Rint = 0.0578) which were used in all calculations. The final wR2 was 0.1571 (all data) and R1 was 0.0563 (I≥2 s(I)).

| Compound 2 | 2010src0454b |
| --- | --- |
|  |  |
| Formula | C21H33B5O13.5P |
| Dcalc./ g cm-3 | 1.375 |
| m/mm-1 | 0.162 |
| Formula Weight | 586.49 |
| Colour | colourless |
| Shape | block-shaped |
| Size/mm3 | 0.260×0.180×0.120 |
| T/K | 120(2) |
| Crystal System | triclinic |
| Space Group | P-1 |
| a/Å | 9.1810(3) |
| b/Å | 13.4812(5) |
| c/Å | 23.8357(8) |
| a/° | 75.3130(10) |
| b/° | 83.095(2) |
| g/° | 86.919(2) |
| V/Å3 | 2832.24(17) |
| Z | 4 |
| Z' | 2 |
| Wavelength/Å | 0.71073 |
| Radiation type | MoKa |
| Qmin/° | 2.941 |
| Qmax/° | 27.605 |
| Measured Refl's. | 50870 |
| Indep't Refl's | 12947 |
| Refl's I≥2 s(I) | 9308 |
| Rint | 0.0578 |
| Parameters | 763 |
| Restraints | 1 |
| Largest Peak | 0.940 |
| Deepest Hole | -0.540 |
| GooF | 1.038 |
| wR2 (all data) | 0.1571 |
| wR2 | 0.1416 |
| R1 (all data) | 0.0831 |
| R1 | 0.0563 |

Structure Quality Indicators

| Reflections: |  |
| --- | --- |
| Refinement: |  |

A colourless block-shaped-shaped crystal with dimensions 0.260 × 0.180 × 0.120 mm3 was mounted. Data were collected using a Damien diffractometer equipped with an Oxford Cryosystems low-temperature device operating at T = 120(2) K.

Data were measured using f & w scans with MoKa radiation. The diffraction pattern was indexed and the total number of runs and images was based on the strategy calculation from the program COLLECT (Hooft, R.W.W., 1998). The maximum resolution that was achieved was Q = 27.605° (0.77 Å).

The unit cell was refined using DENZO (Otwinowski & Minor, 1997) & COLLECT (Hooft, R.W.W., 1998) on 12555 reflections, 25% of the observed reflections.

Data reduction, scaling and absorption corrections were performed using DENZO (Otwinowski & Minor, 1997) & COLLECT (Hooft, R.W.W., 1998). The final completeness is 99.70 % out to 27.605° in Q. SADABS 2007/2 (Sheldrick, G.M., 2007).  The absorption coefficient m of this material is 0.162 mm-1 at this wavelength (l = 0.71073Å) and the minimum and maximum transmissions are 0.959 and 0.981.

The structure was solved and the space group P-1 (# 2) determined by the ShelXS-97 (Sheldrick, 2008) structure solution program using using direct methods and refined by full matrix least squares minimisation on F2 using version 2018/3 of ShelXL 2018/3 (Sheldrick, 2015). All non-hydrogen atoms were refined anisotropically. Hydrogen atom positions were calculated geometrically and refined using the riding model. Hydrogen atom positions were calculated geometrically and refined using the riding model.

_refine_special_details: There is a pseudo body-centring, but multiple reflections are observed which should be absent if it truly existed

_exptl_absorpt_process_details: SADABS 2007/2 (Sheldrick, G.M., 2007)

The value of Z' is 2. This means that there are two independent molecules in the asymmetric unit. The moiety formula is B5 H4 O10, C21 H22 P, 3.5(H2 O).

A structure of a molecule

Description automatically generated

Figure S5 The following hydrogen bonding interactions with a maximum D-D distance of 2.9 Å and a minimum angle of 120 ° are present in 2010src0454b: O7–O62_4: 2.695 Å, O8–O20_5: 2.685 Å, O9–O63_6: 2.874 Å, O10–O7_1: 2.734 Å, O17–O61: 2.871 Å, O18–O19_1: 2.703 Å, O20–O66_5: 2.703 Å, O61–O12: 2.878 Å, O61–O1_6: 2.711 Å, O62–O11: 2.788 Å, O62–O61_2: 2.84 Å, O63–O14: 2.698 Å, O63–O5_6: 2.887 Å, O64–O18_3: 2.936 Å, O64–O8: 2.829 Å, O65–O3: 2.797 Å, O65–O64: 2.714 Å, O66–O10_2: 3.069 Å, O66–O65_2: 2.679 Å, O67–O4: 2.777 Å, O67–O63_4: 2.856 Å, O19–O67_4: 2.699 Å.

A computer generated image of a molecule

Description automatically generated

Figure S6 The p-p interactions of 2010src0454b. Plane 1 to #1@2_575 (-X,2-Y,-Z): The angle between these two planes is 0.000 °, the Centroid-Centroid distance is 3.748 Å and the Shift Distance is 0.917 Å Plane 5 to #5@2_666 (1-X,1-Y,1-Z): The angle between these two planes is 0.000 °, the Centroid-Centroid distance is 3.712 Å and the Shift Distance is 1.155 Å

A diagram of a molecule

Description automatically generated

Figure S7 Packing diagram of 2010src0454b viewed along the a axis

A screenshot of a computer screen

Description automatically generated

Figure S8 Packing diagram of 2010src0454b viewed along the b axis

A close up of a structure

Description automatically generated

Figure S9 Packing diagram of 2010src0454b viewed along the c axis

Table S9: Fractional Atomic Coordinates (×104) and Equivalent Isotropic Displacement Parameters (Å2×103) for 2010src0454b. Ueq is defined as 1/3 of the trace of the orthogonalised Uij.

| Atom | x | y | z | Ueq |
| --- | --- | --- | --- | --- |
| B1 | -21(2) | 4492(2) | 2582.9(11) | 23.8(5) |
| B2 | 2426(2) | 4321(2) | 2949.3(10) | 21.7(5) |
| B3 | 1534(3) | 5989(2) | 2490.3(12) | 25.9(5) |
| B4 | -1784(2) | 3819.3(18) | 2059.7(10) | 19.6(5) |
| B5 | -2637(2) | 4251(2) | 2961.8(11) | 23.9(5) |
| O1 | 1240.3(15) | 3881.2(12) | 2833.4(7) | 23.9(3) |
| O2 | 2557.8(15) | 5367.7(12) | 2820.4(7) | 25.8(3) |
| O3 | 373.4(15) | 5565.8(12) | 2342.6(7) | 27.8(4) |
| O4 | -398.4(14) | 4054.7(11) | 2109.3(6) | 22.3(3) |
| O5 | -2940.2(14) | 3939.2(11) | 2474.6(6) | 21.7(3) |
| O6 | -1259.7(15) | 4434.7(13) | 3039.1(7) | 29.4(4) |
| O7 | 3565.5(15) | 3738.7(12) | 3196.1(7) | 25.0(3) |
| O8 | 1675.3(17) | 7017.6(12) | 2314.1(8) | 36.5(4) |
| O9 | -2078.8(15) | 3462.9(13) | 1599.6(7) | 27.1(4) |
| O10 | -3780.5(16) | 4385.0(15) | 3359.7(7) | 37.8(4) |
| B11 | 4574(2) | 9493.9(19) | -2432.6(11) | 21.8(5) |
| B12 | 2838(2) | 8806.6(18) | -2960.6(10) | 18.8(5) |
| B13 | 1984(2) | 9160(2) | -2037.7(11) | 23.9(5) |
| B14 | 7063(2) | 9341(2) | -2098.9(10) | 21.8(5) |
| B15 | 6112(3) | 10990(2) | -2543.3(11) | 24.2(5) |
| O11 | 4214.9(14) | 9069.7(11) | -2916.5(6) | 20.8(3) |
| O12 | 1687.9(14) | 8877.8(11) | -2535.6(6) | 21.9(3) |
| O13 | 3361.2(15) | 9369.1(12) | -1965.5(6) | 26.0(3) |
| O14 | 5865.8(15) | 8889.4(11) | -2199.0(7) | 23.6(3) |
| O15 | 7195.4(15) | 10386.4(12) | -2245.8(7) | 27.9(4) |
| O16 | 4913.3(15) | 10573.5(11) | -2657.7(7) | 26.5(3) |
| O17 | 2550.5(15) | 8466.4(13) | -3427.9(7) | 26.4(3) |
| O18 | 857.6(16) | 9241.4(16) | -1625.9(8) | 39.5(5) |
| O19 | 8208.0(15) | 8757.3(12) | -1853.4(7) | 27.1(4) |
| O20 | 6348.2(17) | 12022.4(12) | -2719.1(8) | 33.1(4) |
| C1 | 2430(2) | 8949.3(16) | 33.4(9) | 20.5(4) |
| C2 | 2460(2) | 9610.3(17) | -522.9(10) | 27.9(5) |
| C3 | 2076(3) | 10637.2(18) | -576.4(12) | 35.5(6) |
| C4 | 1686(2) | 10997.6(19) | -84.0(13) | 35.5(6) |
| C5 | 1643(3) | 10344(2) | 460.8(13) | 39.6(6) |
| C6 | 2004(3) | 9312.9(18) | 526.7(11) | 32.3(5) |
| C7 | 1105(2) | 6946.0(15) | 226.1(9) | 19.8(4) |
| C8 | 481(2) | 6762.0(17) | -245.9(10) | 23.7(5) |
| C9 | -896(2) | 6327.8(18) | -149.7(11) | 29.2(5) |
| C10 | -1636(2) | 6079.3(17) | 407.6(11) | 29.6(5) |
| C11 | -1019(2) | 6258.8(18) | 875.1(11) | 29.9(5) |
| C12 | 352(2) | 6695.1(17) | 786.8(10) | 25.3(5) |
| C13 | 3772(2) | 7099.3(16) | 745.2(9) | 20.9(4) |
| C14 | 4656(2) | 7726.6(18) | 939.9(10) | 27.6(5) |
| C15 | 5416(2) | 7315(2) | 1418.6(11) | 33.3(6) |
| C16 | 5322(2) | 6280(2) | 1697.5(10) | 31.6(5) |
| C17 | 4480(2) | 5651.7(19) | 1496.0(10) | 29.1(5) |
| C18 | 3692(2) | 6052.4(17) | 1025.8(9) | 24.2(5) |
| C19 | 3962(2) | 7398.8(16) | -525.7(9) | 22.0(4) |
| C20 | 4270(2) | 6249.4(17) | -466.8(10) | 27.7(5) |
| C21 | 5404(2) | 7973.6(19) | -610.5(11) | 31.1(5) |
| P1 | 2813.8(5) | 7602.6(4) | 115.7(2) | 17.53(13) |
| C31 | 1041(2) | 7861.8(17) | 4213.1(9) | 21.6(4) |
| C32 | 1085(2) | 8895.6(18) | 3916.6(10) | 27.3(5) |
| C33 | 281(3) | 9259(2) | 3445.4(11) | 35.0(6) |
| C34 | -556(3) | 8592(2) | 3267.2(10) | 37.3(6) |
| C35 | -616(3) | 7570(2) | 3562.5(11) | 34.4(6) |
| C36 | 170(2) | 7202.9(19) | 4037.7(10) | 28.4(5) |
| C37 | 2498(2) | 6061.1(16) | 4905.0(9) | 20.4(4) |
| C38 | 2544(2) | 5384.6(17) | 5453.6(10) | 26.7(5) |
| C39 | 3044(3) | 4385.5(18) | 5492.2(12) | 33.5(6) |
| C40 | 3482(2) | 4057.1(18) | 4992.2(12) | 33.9(6) |
| C41 | 3438(2) | 4723.8(19) | 4448.6(12) | 34.0(6) |
| C42 | 2962(2) | 5731.0(17) | 4399.0(10) | 26.8(5) |
| C43 | 3701(2) | 8076.6(15) | 4739.0(9) | 19.6(4) |
| C44 | 4297(2) | 8271.8(16) | 5215.3(10) | 23.3(5) |
| C45 | 5664(2) | 8715.3(17) | 5126.3(10) | 27.8(5) |
| C46 | 6432(2) | 8962.6(18) | 4574.6(11) | 30.2(5) |
| C47 | 5846(2) | 8768.2(18) | 4103.4(10) | 28.4(5) |
| C48 | 4482(2) | 8321.9(16) | 4184.5(9) | 23.5(5) |
| C49 | 865(2) | 7563.2(16) | 5486.8(9) | 21.8(4) |
| C50 | -542(2) | 6950.1(19) | 5577.5(10) | 31.0(5) |
| C51 | 494(2) | 8705.9(18) | 5428.7(10) | 28.5(5) |
| P31 | 2023.8(5) | 7391.3(4) | 4840.3(2) | 17.59(13) |
| O61 | -517.3(16) | 8117.1(12) | -3070.2(7) | 27.2(3) |
| O62 | 6688.2(16) | 8316.1(13) | -3490.9(7) | 28.3(4) |
| O63 | 5140.9(16) | 6902.2(12) | -1970.7(7) | 24.4(3) |
| O64 | -252(3) | 8557.0(18) | 1750.5(10) | 62.1(6) |
| O65 | -2000(2) | 6962.5(17) | 2305.4(11) | 56.4(6) |
| O66 | 5825(2) | 6715.0(15) | 3181.5(9) | 47.5(5) |
| O67 | 2061.9(16) | 3299.2(13) | 1537.7(7) | 27.9(4) |

Table S10: Anisotropic Displacement Parameters (×104) for 2010src0454b. The anisotropic displacement factor exponent takes the form: -2p2[h2a*2 × U11+ ... +2hka* × b* × U12]

| Atom | U11 | U22 | U33 | U23 | U13 | U12 |
| --- | --- | --- | --- | --- | --- | --- |
| B1 | 11.9(10) | 33.9(14) | 28.1(14) | -12.1(11) | -1.6(9) | -2.7(9) |
| B2 | 14.1(10) | 34.3(14) | 17.7(12) | -9.1(10) | 2.2(9) | -5.6(9) |
| B3 | 15.3(11) | 32.8(15) | 32.6(15) | -13.4(12) | -2.8(10) | 0.1(10) |
| B4 | 15.3(10) | 23.6(12) | 19.9(12) | -4.7(10) | -2.7(9) | -0.9(9) |
| B5 | 13.6(10) | 36.4(15) | 25.1(13) | -13.1(11) | -2.0(9) | -4.8(9) |
| O1 | 13.6(7) | 30.3(9) | 28.8(9) | -7.5(7) | -4.2(6) | -4.6(6) |
| O2 | 15.0(7) | 32.0(9) | 32.8(9) | -10.4(7) | -4.2(6) | -5.2(6) |
| O3 | 15.7(7) | 28.4(9) | 42.4(10) | -12.6(7) | -7.7(7) | -0.7(6) |
| O4 | 12.9(6) | 31.4(9) | 24.9(8) | -11.2(7) | -1.5(6) | -2.2(6) |
| O5 | 12.3(6) | 33.0(9) | 22.8(8) | -11.6(7) | -2.3(6) | -2.9(6) |
| O6 | 13.4(7) | 53.1(11) | 28.0(9) | -21.2(8) | -1.1(6) | -5.7(7) |
| O7 | 14.6(7) | 33.1(9) | 28.1(9) | -7.7(7) | -4.0(6) | -5.2(6) |
| O8 | 22.7(8) | 29.4(9) | 60.8(12) | -13.8(8) | -11.9(8) | -2.1(7) |
| O9 | 16.7(7) | 42.0(10) | 26.5(9) | -14.9(7) | -3.5(6) | -2.4(7) |
| O10 | 15.0(7) | 71.9(13) | 36.6(10) | -32.2(9) | 2.0(7) | -11.6(8) |
| B11 | 14.0(10) | 28.7(13) | 25.2(13) | -10.3(10) | -2.6(9) | -3.0(9) |
| B12 | 16.1(10) | 20.9(12) | 18.9(12) | -4.1(9) | -1.2(8) | -1.5(9) |
| B13 | 14.9(10) | 33.8(14) | 25.8(13) | -11.9(11) | -3.6(9) | -1.2(9) |
| B14 | 14.1(10) | 33.1(14) | 19.8(12) | -9.6(10) | 0.6(9) | -4.8(9) |
| B15 | 19.3(11) | 27.6(14) | 27.7(14) | -11.5(11) | 1.0(9) | -4.2(9) |
| O11 | 12.7(6) | 27.1(8) | 23.6(8) | -7.7(6) | -2.0(5) | -3.7(6) |
| O12 | 12.9(6) | 33.4(9) | 21.9(8) | -10.4(7) | -2.9(6) | -3.8(6) |
| O13 | 14.3(7) | 42.7(10) | 25.2(8) | -15.1(7) | -2.3(6) | -5.5(6) |
| O14 | 14.4(7) | 27.1(8) | 30.1(9) | -6.4(7) | -6.1(6) | -3.3(6) |
| O15 | 17.6(7) | 31.7(9) | 36.7(10) | -10.6(7) | -4.7(6) | -7.9(6) |
| O16 | 19.8(7) | 24.9(8) | 36.9(9) | -9.0(7) | -7.4(6) | -2.5(6) |
| O17 | 15.9(7) | 41.3(10) | 26.1(8) | -14.9(7) | -2.7(6) | -3.6(6) |
| O18 | 15.5(7) | 78.2(14) | 35.1(10) | -33.1(9) | 2.1(7) | -12.3(8) |
| O19 | 14.4(7) | 36.3(9) | 32.5(9) | -9.6(7) | -5.9(6) | -4.7(6) |
| O20 | 27.3(8) | 28.4(9) | 46.6(11) | -12.8(8) | -6.8(8) | -5.1(7) |
| C1 | 12.3(9) | 22.6(11) | 26.7(12) | -6.2(9) | -2.8(8) | 0.4(8) |
| C2 | 28.1(11) | 24.8(12) | 32.7(13) | -6.9(10) | -12.5(10) | 0.5(9) |
| C3 | 33.9(13) | 22.3(12) | 49.4(16) | 0.4(11) | -20.6(12) | -2.0(10) |
| C4 | 17.7(10) | 23.3(12) | 67.1(19) | -13.7(12) | -6.3(11) | 2.0(9) |
| C5 | 34.8(13) | 31.8(14) | 52.9(18) | -19.9(13) | 12.5(12) | 0.0(11) |
| C6 | 32.9(12) | 29.0(13) | 33.5(14) | -11.7(11) | 10.9(10) | -0.4(10) |
| C7 | 17.7(9) | 19.2(11) | 22.9(11) | -5.4(8) | -3.6(8) | 0.4(8) |
| C8 | 21.8(10) | 27.0(12) | 24.0(12) | -9.5(9) | -3.8(8) | 1.1(8) |
| C9 | 25.7(11) | 30.7(13) | 37.1(14) | -15.5(11) | -12.1(10) | 0.0(9) |
| C10 | 21.2(11) | 23.6(12) | 43.2(15) | -5.5(10) | -5.5(10) | -4.7(9) |
| C11 | 22.1(11) | 34.2(13) | 29.2(13) | -0.4(10) | 0.3(9) | -5.6(9) |
| C12 | 21.4(10) | 32.2(13) | 21.1(11) | -3.8(9) | -2.6(8) | -3.8(9) |
| C13 | 17.0(9) | 26.2(12) | 19.0(11) | -5.8(9) | -0.8(8) | 0.8(8) |
| C14 | 23.2(11) | 31.7(13) | 26.9(12) | -3.8(10) | -4.4(9) | -6.3(9) |
| C15 | 23.9(11) | 49.0(16) | 27.7(13) | -7.1(11) | -7.5(9) | -8.4(10) |
| C16 | 22.6(11) | 46.5(15) | 22.6(12) | -1.8(11) | -6.4(9) | 3.6(10) |
| C17 | 26.5(11) | 31.2(13) | 26.6(12) | -2.3(10) | -3.8(9) | 4.1(9) |
| C18 | 23.4(10) | 23.9(12) | 24.4(12) | -4.0(9) | -3.1(9) | -0.6(8) |
| C19 | 20.1(10) | 25.4(11) | 20.3(11) | -6.0(9) | -1.6(8) | 1.1(8) |
| C20 | 30.2(11) | 26.2(12) | 28.6(13) | -11.0(10) | -3.8(9) | 5.7(9) |
| C21 | 21.3(11) | 40.3(14) | 30.5(13) | -10.7(11) | 7.6(9) | -4.6(10) |
| P1 | 15.3(2) | 19.1(3) | 18.0(3) | -4.5(2) | -1.13(19) | -0.17(19) |
| C31 | 16.8(9) | 27.5(12) | 19.3(11) | -4.3(9) | -1.0(8) | 1.7(8) |
| C32 | 23.4(11) | 29.5(13) | 26.8(12) | -3.8(10) | -2.1(9) | 2.9(9) |
| C33 | 28.7(12) | 38.4(15) | 28.8(13) | 5.1(11) | -1.6(10) | 10.0(10) |
| C34 | 24.6(11) | 62.2(18) | 20.7(12) | -2.2(12) | -6.9(9) | 9.6(11) |
| C35 | 25.4(11) | 53.0(17) | 26.5(13) | -10.1(12) | -8.1(10) | -4.1(11) |
| C36 | 22.5(11) | 35.2(13) | 26.5(12) | -4.3(10) | -4.7(9) | -3.4(9) |
| C37 | 15.4(9) | 22.1(11) | 24.5(11) | -6.0(9) | -4.0(8) | -0.7(8) |
| C38 | 27.6(11) | 24.3(12) | 30.6(13) | -7.4(10) | -11.0(9) | -1.0(9) |
| C39 | 30.5(12) | 23.7(12) | 46.1(15) | -0.7(11) | -19.5(11) | -2.3(9) |
| C40 | 19.8(11) | 22.7(12) | 63.3(18) | -16.2(12) | -9.6(11) | 0.9(9) |
| C41 | 21.7(11) | 34.3(14) | 49.9(16) | -22.1(12) | 6.0(10) | -1.4(10) |
| C42 | 24.1(11) | 28.0(12) | 29.1(13) | -10.7(10) | 2.7(9) | -2.0(9) |
| C43 | 16.5(9) | 17.3(10) | 24.4(11) | -4.4(8) | -2.5(8) | 0.9(8) |
| C44 | 22.0(10) | 25.4(12) | 24.2(12) | -8.8(9) | -3.4(8) | -0.4(8) |
| C45 | 24.2(11) | 30.7(13) | 32.1(13) | -12.1(10) | -8.1(9) | -0.1(9) |
| C46 | 20.3(10) | 27.5(13) | 42.8(15) | -6.7(11) | -6.1(10) | -4.3(9) |
| C47 | 20.4(10) | 30.9(13) | 29.6(13) | -1.2(10) | 1.5(9) | -3.5(9) |
| C48 | 20.3(10) | 27.5(12) | 21.8(11) | -5.2(9) | -1.4(8) | -1.3(8) |
| C49 | 17.9(9) | 26.9(12) | 20.9(11) | -8.0(9) | 0.4(8) | 1.0(8) |
| C50 | 22.6(11) | 40.7(14) | 29.0(13) | -10.1(11) | 5.4(9) | -5.6(10) |
| C51 | 24.4(11) | 33.4(13) | 30.2(13) | -14.1(10) | -2.5(9) | 5.7(9) |
| P31 | 15.1(2) | 20.3(3) | 17.4(3) | -4.8(2) | -1.97(19) | 0.30(19) |
| O61 | 22.3(7) | 31.6(9) | 29.9(9) | -9.3(7) | -5.7(6) | -6.8(7) |
| O62 | 18.6(7) | 38.0(10) | 30.8(9) | -13.3(7) | -3.9(6) | 1.9(7) |
| O63 | 21.0(7) | 25.9(8) | 27.5(9) | -6.0(7) | -7.3(6) | -3.8(6) |
| O64 | 60.8(14) | 69.3(16) | 48.3(14) | -3.3(12) | -7.9(11) | 17.1(12) |
| O65 | 37.5(11) | 60.0(14) | 80.6(17) | -28.9(13) | -24.4(11) | 16.1(9) |
| O66 | 57.2(13) | 39.2(11) | 49.1(13) | -14.2(9) | -15.5(10) | 8.3(9) |
| O67 | 17.1(7) | 39.4(10) | 30.5(9) | -14.9(7) | -4.2(6) | 3.1(7) |

Table S11: Bond Lengths in Å for 2010src0454b.

| Atom | Atom | Length/Å |
| --- | --- | --- |
| B1 | O1 | 1.480(3) |  |
| B1 | O3 | 1.463(3) |  |
| B1 | O4 | 1.482(3) |  |
| B1 | O6 | 1.467(3) |  |
| B2 | O1 | 1.356(3) |  |
| B2 | O2 | 1.374(3) |  |
| B2 | O7 | 1.372(3) |  |
| B3 | O2 | 1.401(3) |  |
| B3 | O3 | 1.357(3) |  |
| B3 | O8 | 1.352(3) |  |
| B4 | O4 | 1.354(3) |  |
| B4 | O5 | 1.393(3) |  |
| B4 | O9 | 1.364(3) |  |
| B5 | O5 | 1.393(3) |  |
| B5 | O6 | 1.344(3) |  |
| B5 | O10 | 1.364(3) |  |
| B11 | O11 | 1.487(3) |  |
| B11 | O13 | 1.461(3) |  |
| B11 | O14 | 1.485(3) |  |
| B11 | O16 | 1.455(3) |  |
| B12 | O11 | 1.354(2) |  |
| B12 | O12 | 1.390(3) |  |
| B12 | O17 | 1.365(3) |  |
| B13 | O12 | 1.395(3) |  |
| B13 | O13 | 1.350(3) |  |
| B13 | O18 | 1.358(3) |  |
| B14 | O14 | 1.358(3) |  |
| B14 | O15 | 1.372(3) |  |
| B14 | O19 | 1.376(3) |  |
| B15 | O15 | 1.395(3) |  |
| B15 | O16 | 1.348(3) |  |
| B15 | O20 | 1.369(3) |  |
| C1 | C2 | 1.396(3) |  |
| C1 | C6 | 1.392(3) |  |
| C1 | P1 | 1.796(2) |  |
| C2 | C3 | 1.388(3) |  |
| C3 | C4 | 1.383(4) |  |
| C4 | C5 | 1.369(4) |  |
| C5 | C6 | 1.386(3) |  |
| C7 | C8 | 1.402(3) |  |
| C7 | C12 | 1.397(3) |  |
| C7 | P1 | 1.802(2) |  |
| C8 | C9 | 1.391(3) |  |
| C9 | C10 | 1.386(3) |  |
| C10 | C11 | 1.387(3) |  |
| C11 | C12 | 1.389(3) |  |
| C13 | C14 | 1.398(3) |  |
| C13 | C18 | 1.402(3) |  |
| C13 | P1 | 1.795(2) |  |
| C14 | C15 | 1.387(3) |  |
| C15 | C16 | 1.387(4) |  |
| C16 | C17 | 1.383(3) |  |
| C17 | C18 | 1.385(3) |  |
| C19 | C20 | 1.534(3) |  |
| C19 | C21 | 1.537(3) |  |
| C19 | P1 | 1.821(2) |  |
| C31 | C32 | 1.394(3) |  |
| C31 | C36 | 1.396(3) |  |
| C31 | P31 | 1.798(2) |  |
| C32 | C33 | 1.388(3) |  |
| C33 | C34 | 1.388(4) |  |
| C34 | C35 | 1.380(4) |  |
| C35 | C36 | 1.387(3) |  |
| C37 | C38 | 1.396(3) |  |
| C37 | C42 | 1.401(3) |  |
| C37 | P31 | 1.795(2) |  |
| C38 | C39 | 1.384(3) |  |
| C39 | C40 | 1.383(4) |  |
| C40 | C41 | 1.381(4) |  |
| C41 | C42 | 1.384(3) |  |
| C43 | C44 | 1.405(3) |  |
| C43 | C48 | 1.395(3) |  |
| C43 | P31 | 1.798(2) |  |
| C44 | C45 | 1.390(3) |  |
| C45 | C46 | 1.384(3) |  |
| C46 | C47 | 1.389(3) |  |
| C47 | C48 | 1.390(3) |  |
| C49 | C50 | 1.535(3) |  |
| C49 | C51 | 1.535(3) |  |
| C49 | P31 | 1.822(2) |  |

Table S12: Bond Angles in ° for 2010src0454b.

| Atom | Atom | Atom | Angle/° |
| --- | --- | --- | --- |
| O1 | B1 | O4 | 107.16(17) |  |
| O3 | B1 | O1 | 110.41(16) |  |
| O3 | B1 | O4 | 109.53(19) |  |
| O3 | B1 | O6 | 109.04(18) |  |
| O6 | B1 | O1 | 109.58(19) |  |
| O6 | B1 | O4 | 111.10(16) |  |
| O1 | B2 | O2 | 122.0(2) |  |
| O1 | B2 | O7 | 121.4(2) |  |
| O7 | B2 | O2 | 116.67(18) |  |
| O3 | B3 | O2 | 120.4(2) |  |
| O8 | B3 | O2 | 120.97(19) |  |
| O8 | B3 | O3 | 118.7(2) |  |
| O4 | B4 | O5 | 120.91(19) |  |
| O4 | B4 | O9 | 120.53(19) |  |
| O9 | B4 | O5 | 118.55(18) |  |
| O6 | B5 | O5 | 121.48(19) |  |
| O6 | B5 | O10 | 120.15(19) |  |
| O10 | B5 | O5 | 118.36(18) |  |
| B2 | O1 | B1 | 122.38(18) |  |
| B2 | O2 | B3 | 118.55(17) |  |
| B3 | O3 | B1 | 123.39(18) |  |
| B4 | O4 | B1 | 123.04(16) |  |
| B4 | O5 | B5 | 118.87(16) |  |
| B5 | O6 | B1 | 122.94(17) |  |
| O13 | B11 | O11 | 111.19(16) |  |
| O13 | B11 | O14 | 108.68(18) |  |
| O14 | B11 | O11 | 106.24(17) |  |
| O16 | B11 | O11 | 109.48(18) |  |
| O16 | B11 | O13 | 110.15(18) |  |
| O16 | B11 | O14 | 111.03(16) |  |
| O11 | B12 | O12 | 121.15(19) |  |
| O11 | B12 | O17 | 120.38(19) |  |
| O17 | B12 | O12 | 118.46(18) |  |
| O13 | B13 | O12 | 121.1(2) |  |
| O13 | B13 | O18 | 119.9(2) |  |
| O18 | B13 | O12 | 118.99(18) |  |
| O14 | B14 | O15 | 121.9(2) |  |
| O14 | B14 | O19 | 120.7(2) |  |
| O15 | B14 | O19 | 117.38(18) |  |
| O16 | B15 | O15 | 121.6(2) |  |
| O16 | B15 | O20 | 122.2(2) |  |
| O20 | B15 | O15 | 116.21(19) |  |
| B12 | O11 | B11 | 122.58(16) |  |
| B12 | O12 | B13 | 119.02(16) |  |
| B13 | O13 | B11 | 122.97(17) |  |
| B14 | O14 | B11 | 122.21(18) |  |
| B14 | O15 | B15 | 118.40(17) |  |
| B15 | O16 | B11 | 123.18(18) |  |
| C2 | C1 | P1 | 120.05(16) |  |
| C6 | C1 | C2 | 120.4(2) |  |
| C6 | C1 | P1 | 119.37(17) |  |
| C3 | C2 | C1 | 119.0(2) |  |
| C4 | C3 | C2 | 120.2(2) |  |
| C5 | C4 | C3 | 120.6(2) |  |
| C4 | C5 | C6 | 120.4(2) |  |
| C5 | C6 | C1 | 119.3(2) |  |
| C8 | C7 | P1 | 120.97(16) |  |
| C12 | C7 | C8 | 120.37(19) |  |
| C12 | C7 | P1 | 118.47(15) |  |
| C9 | C8 | C7 | 119.3(2) |  |
| C10 | C9 | C8 | 120.1(2) |  |
| C9 | C10 | C11 | 120.7(2) |  |
| C10 | C11 | C12 | 120.0(2) |  |
| C11 | C12 | C7 | 119.6(2) |  |
| C14 | C13 | C18 | 119.6(2) |  |
| C14 | C13 | P1 | 120.80(17) |  |
| C18 | C13 | P1 | 119.48(16) |  |
| C15 | C14 | C13 | 119.8(2) |  |
| C16 | C15 | C14 | 120.3(2) |  |
| C17 | C16 | C15 | 120.1(2) |  |
| C16 | C17 | C18 | 120.5(2) |  |
| C17 | C18 | C13 | 119.7(2) |  |
| C20 | C19 | C21 | 110.60(17) |  |
| C20 | C19 | P1 | 110.42(15) |  |
| C21 | C19 | P1 | 110.08(15) |  |
| C1 | P1 | C7 | 108.96(9) |  |
| C1 | P1 | C19 | 109.87(10) |  |
| C7 | P1 | C19 | 111.31(10) |  |
| C13 | P1 | C1 | 109.89(10) |  |
| C13 | P1 | C7 | 108.13(10) |  |
| C13 | P1 | C19 | 108.65(9) |  |
| C32 | C31 | C36 | 119.5(2) |  |
| C32 | C31 | P31 | 120.34(16) |  |
| C36 | C31 | P31 | 120.07(17) |  |
| C33 | C32 | C31 | 119.9(2) |  |
| C32 | C33 | C34 | 120.0(2) |  |
| C35 | C34 | C33 | 120.3(2) |  |
| C34 | C35 | C36 | 120.0(2) |  |
| C35 | C36 | C31 | 120.2(2) |  |
| C38 | C37 | C42 | 120.2(2) |  |
| C38 | C37 | P31 | 120.44(16) |  |
| C42 | C37 | P31 | 119.08(17) |  |
| C39 | C38 | C37 | 119.4(2) |  |
| C40 | C39 | C38 | 120.3(2) |  |
| C41 | C40 | C39 | 120.5(2) |  |
| C40 | C41 | C42 | 120.3(2) |  |
| C41 | C42 | C37 | 119.4(2) |  |
| C44 | C43 | P31 | 121.06(16) |  |
| C48 | C43 | C44 | 120.14(19) |  |
| C48 | C43 | P31 | 118.49(15) |  |
| C45 | C44 | C43 | 119.2(2) |  |
| C46 | C45 | C44 | 120.4(2) |  |
| C45 | C46 | C47 | 120.4(2) |  |
| C46 | C47 | C48 | 120.0(2) |  |
| C47 | C48 | C43 | 119.8(2) |  |
| C50 | C49 | C51 | 110.61(17) |  |
| C50 | C49 | P31 | 110.30(15) |  |
| C51 | C49 | P31 | 110.12(15) |  |
| C31 | P31 | C43 | 109.40(10) |  |
| C31 | P31 | C49 | 108.88(9) |  |
| C37 | P31 | C31 | 108.97(10) |  |
| C37 | P31 | C43 | 107.84(9) |  |
| C37 | P31 | C49 | 110.84(10) |  |
| C43 | P31 | C49 | 110.87(10) |  |

Table S13: Torsion Angles in ° for 2010src0454b.

| Atom | Atom | Atom | Atom | Angle/° |  |
| --- | --- | --- | --- | --- | --- |
| O1 | B1 | O3 | B3 | -19.0(3) |
| O1 | B1 | O4 | B4 | 131.12(19) |
| O1 | B1 | O6 | B5 | -133.5(2) |
| O1 | B2 | O2 | B3 | -8.7(3) |
| O2 | B2 | O1 | B1 | -2.5(3) |
| O2 | B3 | O3 | B1 | 9.7(3) |
| O3 | B1 | O1 | B2 | 15.3(3) |
| O3 | B1 | O4 | B4 | -109.1(2) |
| O3 | B1 | O6 | B5 | 105.5(2) |
| O3 | B3 | O2 | B2 | 5.2(3) |
| O4 | B1 | O1 | B2 | 134.55(19) |
| O4 | B1 | O3 | B3 | -136.7(2) |
| O4 | B1 | O6 | B5 | -15.3(3) |
| O4 | B4 | O5 | B5 | -3.6(3) |
| O5 | B4 | O4 | B1 | -2.8(3) |
| O5 | B5 | O6 | B1 | 10.5(4) |
| O6 | B1 | O1 | B2 | -104.8(2) |
| O6 | B1 | O3 | B3 | 101.5(2) |
| O6 | B1 | O4 | B4 | 11.4(3) |
| O6 | B5 | O5 | B4 | -0.2(3) |
| O7 | B2 | O1 | B1 | 178.25(19) |
| O7 | B2 | O2 | B3 | 170.57(19) |
| O8 | B3 | O2 | B2 | -175.0(2) |
| O8 | B3 | O3 | B1 | -170.1(2) |
| O9 | B4 | O4 | B1 | 177.0(2) |
| O9 | B4 | O5 | B5 | 176.6(2) |
| O10 | B5 | O5 | B4 | 178.8(2) |
| O10 | B5 | O6 | B1 | -168.4(2) |
| O11 | B11 | O13 | B13 | -16.7(3) |
| O11 | B11 | O14 | B14 | 132.28(19) |
| O11 | B11 | O16 | B15 | -131.3(2) |
| O11 | B12 | O12 | B13 | -4.3(3) |
| O12 | B12 | O11 | B11 | -3.0(3) |
| O12 | B13 | O13 | B11 | 11.1(3) |
| O13 | B11 | O11 | B12 | 12.6(3) |
| O13 | B11 | O14 | B14 | -108.0(2) |
| O13 | B11 | O16 | B15 | 106.2(2) |
| O13 | B13 | O12 | B12 | 0.4(3) |
| O14 | B11 | O11 | B12 | 130.70(19) |
| O14 | B11 | O13 | B13 | -133.3(2) |
| O14 | B11 | O16 | B15 | -14.3(3) |
| O14 | B14 | O15 | B15 | -4.6(3) |
| O15 | B14 | O14 | B11 | -4.7(3) |
| O15 | B15 | O16 | B11 | 6.6(3) |
| O16 | B11 | O11 | B12 | -109.3(2) |
| O16 | B11 | O13 | B13 | 104.9(2) |
| O16 | B11 | O14 | B14 | 13.3(3) |
| O16 | B15 | O15 | B14 | 3.8(3) |
| O17 | B12 | O11 | B11 | 176.84(19) |
| O17 | B12 | O12 | B13 | 175.9(2) |
| O18 | B13 | O12 | B12 | 179.2(2) |
| O18 | B13 | O13 | B11 | -167.8(2) |
| O19 | B14 | O14 | B11 | 176.54(19) |
| O19 | B14 | O15 | B15 | 174.25(19) |
| O20 | B15 | O15 | B14 | -175.23(19) |
| O20 | B15 | O16 | B11 | -174.5(2) |
| C1 | C2 | C3 | C4 | 0.7(3) |
| C2 | C1 | C6 | C5 | -1.6(3) |
| C2 | C1 | P1 | C7 | -96.67(18) |
| C2 | C1 | P1 | C13 | 145.03(16) |
| C2 | C1 | P1 | C19 | 25.53(19) |
| C2 | C3 | C4 | C5 | -1.4(3) |
| C3 | C4 | C5 | C6 | 0.7(4) |
| C4 | C5 | C6 | C1 | 0.8(4) |
| C6 | C1 | C2 | C3 | 0.8(3) |
| C6 | C1 | P1 | C7 | 78.79(19) |
| C6 | C1 | P1 | C13 | -39.51(19) |
| C6 | C1 | P1 | C19 | -159.01(17) |
| C7 | C8 | C9 | C10 | -0.1(3) |
| C8 | C7 | C12 | C11 | 0.2(3) |
| C8 | C7 | P1 | C1 | 92.88(19) |
| C8 | C7 | P1 | C13 | -147.71(17) |
| C8 | C7 | P1 | C19 | -28.4(2) |
| C8 | C9 | C10 | C11 | 0.0(3) |
| C9 | C10 | C11 | C12 | 0.2(4) |
| C10 | C11 | C12 | C7 | -0.3(3) |
| C12 | C7 | C8 | C9 | 0.1(3) |
| C12 | C7 | P1 | C1 | -82.12(18) |
| C12 | C7 | P1 | C13 | 37.29(19) |
| C12 | C7 | P1 | C19 | 156.56(16) |
| C13 | C14 | C15 | C16 | 1.2(4) |
| C14 | C13 | C18 | C17 | 0.6(3) |
| C14 | C13 | P1 | C1 | -27.9(2) |
| C14 | C13 | P1 | C7 | -146.74(17) |
| C14 | C13 | P1 | C19 | 92.32(19) |
| C14 | C15 | C16 | C17 | 0.6(4) |
| C15 | C16 | C17 | C18 | -1.8(4) |
| C16 | C17 | C18 | C13 | 1.2(3) |
| C18 | C13 | C14 | C15 | -1.8(3) |
| C18 | C13 | P1 | C1 | 155.49(16) |
| C18 | C13 | P1 | C7 | 36.67(19) |
| C18 | C13 | P1 | C19 | -84.27(18) |
| C20 | C19 | P1 | C1 | -178.79(14) |
| C20 | C19 | P1 | C7 | -58.00(17) |
| C20 | C19 | P1 | C13 | 60.95(17) |
| C21 | C19 | P1 | C1 | 58.82(17) |
| C21 | C19 | P1 | C7 | 179.61(15) |
| C21 | C19 | P1 | C13 | -61.44(18) |
| P1 | C1 | C2 | C3 | 176.25(16) |
| P1 | C1 | C6 | C5 | -177.04(18) |
| P1 | C7 | C8 | C9 | -174.84(16) |
| P1 | C7 | C12 | C11 | 175.19(17) |
| P1 | C13 | C14 | C15 | -178.35(17) |
| P1 | C13 | C18 | C17 | 177.20(16) |
| C31 | C32 | C33 | C34 | -0.5(3) |
| C32 | C31 | C36 | C35 | 1.7(3) |
| C32 | C31 | P31 | C37 | -152.52(17) |
| C32 | C31 | P31 | C43 | -34.8(2) |
| C32 | C31 | P31 | C49 | 86.47(19) |
| C32 | C33 | C34 | C35 | 1.1(4) |
| C33 | C34 | C35 | C36 | -0.4(4) |
| C34 | C35 | C36 | C31 | -1.0(4) |
| C36 | C31 | C32 | C33 | -0.9(3) |
| C36 | C31 | P31 | C37 | 30.7(2) |
| C36 | C31 | P31 | C43 | 148.35(17) |
| C36 | C31 | P31 | C49 | -90.34(19) |
| C37 | C38 | C39 | C40 | -0.5(3) |
| C38 | C37 | C42 | C41 | 1.3(3) |
| C38 | C37 | P31 | C31 | -146.52(16) |
| C38 | C37 | P31 | C43 | 94.81(18) |
| C38 | C37 | P31 | C49 | -26.73(19) |
| C38 | C39 | C40 | C41 | 0.6(3) |
| C39 | C40 | C41 | C42 | 0.3(3) |
| C40 | C41 | C42 | C37 | -1.2(3) |
| C42 | C37 | C38 | C39 | -0.4(3) |
| C42 | C37 | P31 | C31 | 39.81(19) |
| C42 | C37 | P31 | C43 | -78.85(18) |
| C42 | C37 | P31 | C49 | 159.61(16) |
| C43 | C44 | C45 | C46 | 0.0(3) |
| C44 | C43 | C48 | C47 | -0.5(3) |
| C44 | C43 | P31 | C31 | 147.61(17) |
| C44 | C43 | P31 | C37 | -94.01(18) |
| C44 | C43 | P31 | C49 | 27.5(2) |
| C44 | C45 | C46 | C47 | -0.2(3) |
| C45 | C46 | C47 | C48 | 0.0(3) |
| C46 | C47 | C48 | C43 | 0.4(3) |
| C48 | C43 | C44 | C45 | 0.3(3) |
| C48 | C43 | P31 | C31 | -38.77(19) |
| C48 | C43 | P31 | C37 | 79.61(18) |
| C48 | C43 | P31 | C49 | -158.87(16) |
| C50 | C49 | P31 | C31 | 61.19(18) |
| C50 | C49 | P31 | C37 | -58.66(17) |
| C50 | C49 | P31 | C43 | -178.40(15) |
| C51 | C49 | P31 | C31 | -61.17(16) |
| C51 | C49 | P31 | C37 | 178.98(14) |
| C51 | C49 | P31 | C43 | 59.24(16) |
| P31 | C31 | C32 | C33 | -177.73(17) |
| P31 | C31 | C36 | C35 | 178.48(17) |
| P31 | C37 | C38 | C39 | -173.99(16) |
| P31 | C37 | C42 | C41 | 174.97(16) |
| P31 | C43 | C44 | C45 | 173.84(16) |
| P31 | C43 | C48 | C47 | -174.22(17) |

Table S14: Hydrogen Fractional Atomic Coordinates (×104) and Equivalent Isotropic Displacement Parameters (Å2×103) for 2010src0454b. Ueq is defined as 1/3 of the trace of the orthogonalised Uij.

| Atom | x | y | z | Ueq |
| --- | --- | --- | --- | --- |
| H7 | 3372.18 | 3114.26 | 3266.71 | 37 |
| H8A | 2375.5 | 7189.35 | 2463.1 | 55 |
| H9A | -2984.18 | 3371.48 | 1622.79 | 41 |
| H10A | -4543.55 | 4146.1 | 3287.41 | 57 |
| H17A | 1660.29 | 8320.37 | -3390.73 | 40 |
| H18A | 81.14 | 9051.02 | -1716.59 | 59 |
| H19A | 8024.87 | 8132.46 | -1790.71 | 41 |
| H20 | 5600.84 | 12328.89 | -2857.82 | 50 |
| H2 | 2738.33 | 9360.84 | -860.01 | 33 |
| H3 | 2081.06 | 11093.92 | -952.61 | 43 |
| H4 | 1446.83 | 11704.61 | -123.93 | 43 |
| H5 | 1363.42 | 10600.02 | 795.72 | 48 |
| H6 | 1960.55 | 8858.66 | 904.66 | 39 |
| H8 | 992.87 | 6932.03 | -627.07 | 28 |
| H9 | -1329.56 | 6201.38 | -466.56 | 35 |
| H10 | -2576.27 | 5782.91 | 470.24 | 35 |
| H11 | -1534.42 | 6083.27 | 1255.65 | 36 |
| H12 | 775.94 | 6822.07 | 1105.64 | 30 |
| H14 | 4737.07 | 8432.71 | 745.14 | 33 |
| H15 | 6003.32 | 7743.21 | 1555.75 | 40 |
| H16 | 5836.37 | 6003.12 | 2027.46 | 38 |
| H17 | 4442.17 | 4939.83 | 1681.45 | 35 |
| H18 | 3100.32 | 5619.73 | 893.85 | 29 |
| H19 | 3425.61 | 7677.85 | -875.86 | 26 |
| H20A | 4842.69 | 6149.85 | -823.65 | 42 |
| H20B | 3339.38 | 5893.88 | -406.17 | 42 |
| H20C | 4826.38 | 5970.21 | -133.02 | 42 |
| H21A | 5931.96 | 7718.01 | -266.4 | 47 |
| H21B | 5187.74 | 8709.04 | -662.64 | 47 |
| H21C | 6013.32 | 7860.08 | -956.61 | 47 |
| H32 | 1663.65 | 9350.89 | 4036.8 | 33 |
| H33 | 304.34 | 9964.48 | 3244.93 | 42 |
| H34 | -1091.03 | 8840.24 | 2940.19 | 45 |
| H35 | -1195.75 | 7117.6 | 3440.18 | 41 |
| H36 | 115.22 | 6501.46 | 4244.46 | 34 |
| H38 | 2235.98 | 5608.19 | 5797.34 | 32 |
| H39 | 3087.24 | 3922.73 | 5864.31 | 40 |
| H40 | 3814.87 | 3367.9 | 5022.77 | 41 |
| H41 | 3736.13 | 4490.38 | 4107.35 | 41 |
| H42 | 2949.53 | 6193.96 | 4025.34 | 32 |
| H44 | 3770.26 | 8102.59 | 5593.95 | 28 |
| H45 | 6075.21 | 8849.56 | 5446.04 | 33 |
| H46 | 7364.76 | 9267.31 | 4518.11 | 36 |
| H47 | 6377.98 | 8940.05 | 3725.87 | 34 |
| H48 | 4082.54 | 8184.29 | 3863.23 | 28 |
| H49 | 1416.42 | 7301.23 | 5834.17 | 26 |
| H50A | -1143.35 | 7035.75 | 5931.42 | 46 |
| H50B | -284.34 | 6222.16 | 5618.65 | 46 |
| H50C | -1095.84 | 7200.9 | 5240.55 | 46 |
| H51A | -71.85 | 8968.35 | 5096.21 | 43 |
| H51B | 1404.25 | 9085.95 | 5366.37 | 43 |
| H51C | -86.92 | 8793.03 | 5786.52 | 43 |
| H61A | -250.21 | 8265.1 | -2763.56 | 41 |
| H61B | -691.84 | 7464.52 | -2958.77 | 41 |
| H62A | 5960.61 | 8640.33 | -3340.39 | 42 |
| H62B | 7446.29 | 8445.32 | -3337.11 | 42 |
| H63A | 5362.57 | 7546.61 | -2088.5 | 37 |
| H63B | 4889.22 | 6749.75 | -2278.75 | 37 |
| H64A | 518.61 | 8413.44 | 1938.97 | 93 |
| H64B | -441.32 | 9203.34 | 1730.99 | 93 |
| H65A | -1583.22 | 7549.21 | 2154.15 | 85 |
| H65B | -1273.76 | 6519.81 | 2358.7 | 85 |
| H66A | 6526.28 | 6849.47 | 2895.27 | 71 |
| H66B | 5608.65 | 6082.09 | 3211.08 | 71 |
| H67A | 1325.9 | 3594.9 | 1702.74 | 42 |
| H67B | 2818.99 | 3426.48 | 1692.98 | 42 |

Table S15: Hydrogen Bond information for 2010src0454b.

| D | H | A | d(D-H)/Å | d(H-A)/Å | d(D-A)/Å | D-H-A/deg |
| --- | --- | --- | --- | --- | --- | --- |
| O7 | H7 | O621 | 0.84 | 1.87 | 2.695(2) | 168.1 |
| O8 | H8A | O202 | 0.84 | 1.87 | 2.685(2) | 161.9 |
| O9 | H9A | O633 | 0.84 | 2.07 | 2.874(2) | 160.8 |
| O10 | H10A | O74 | 0.84 | 1.90 | 2.734(2) | 170.9 |
| O17 | H17A | O61 | 0.84 | 2.06 | 2.871(2) | 162.3 |
| O18 | H18A | O194 | 0.84 | 1.87 | 2.703(2) | 171.4 |
| O20 | H20 | O662 | 0.84 | 1.88 | 2.703(3) | 166.8 |
| O61 | H61A | O12 | 0.87 | 2.18 | 2.8776(19) | 136.7 |
| O61 | H61B | O13 | 0.87 | 1.85 | 2.711(2) | 170.1 |
| O62 | H62A | O11 | 0.87 | 1.93 | 2.788(2) | 167.8 |
| O62 | H62B | O615 | 0.87 | 2.04 | 2.840(2) | 153.2 |
| O63 | H63A | O14 | 0.87 | 1.84 | 2.698(2) | 169.7 |
| O63 | H63B | O53 | 0.87 | 2.21 | 2.8865(19) | 134.5 |
| O64 | H64A | O8 | 0.87 | 2.15 | 2.829(3) | 134.4 |
| O64 | H64B | O186 | 0.87 | 2.07 | 2.936(3) | 176.2 |
| O65 | H65A | O64 | 0.87 | 1.87 | 2.714(3) | 162.9 |
| O65 | H65B | O3 | 0.87 | 1.94 | 2.797(2) | 170.8 |
| O66 | H66A | O655 | 0.87 | 1.81 | 2.679(3) | 173.0 |
| O66 | H66B | O105 | 0.87 | 2.27 | 3.069(3) | 151.8 |
| O67 | H67A | O4 | 0.87 | 1.91 | 2.777(2) | 171.9 |
| O67 | H67B | O631 | 0.87 | 2.05 | 2.856(2) | 153.6 |

–––– 

11-x,1-y,-z; 21-x,2-y,-z; 3-x,1-y,-z; 4-1+x,+y,+z; 51+x,+y,+z; 6-x,2-y,-z

Citations for 2

COLLECT (Hooft, R.W.W., 1998)

DENZO (Otwinowski & Minor, 1997)

O.V. Dolomanov and L.J. Bourhis and R.J. Gildea and J.A.K. Howard and H. Puschmann, Olex2: A complete structure solution, refinement and analysis program, J. Appl. Cryst., (2009), 42, 339-341.

Sheldrick, G.M., A short history of ShelX, Acta Cryst., (2008), A64, 339-341.

Sheldrick, G.M., Crystal structure refinement with ShelXL, Acta Cryst., (2015), C71, 3-8.

TGA plots for [MePPh3][B5O6(OH)4].2H2O (1) and [iPrPPh3][B5O6(OH)4].3.5H2O (2).

	Chart, line chart

Description automatically generated

Figure  S10. TGA data for the thermal decomposition of tetraalkylphosphonium
pentaborate salts. The plot labelled ‘36’ is [MePPh3][B5O6(OH)4].2H2O (1) that labelled ‘37’ is [iPrPPh3][B5O6(OH)4].3.5H2O (2). For comparison the line labelled ‘34’ is [PPh4][B5O6(OH)4].1.5H2O. (Taken from Ref. 39 in Manuscript).
